# Whole-exome sequencing reveals damaging gene variants associated with hypoalphalipoproteinemia

**DOI:** 10.1016/j.jlr.2022.100209

**Published:** 2022-04-20

**Authors:** Weilai Dong, Karen H.Y. Wong, Youbin Liu, Michal Levy-Sakin, Wei-Chien Hung, Mo Li, Boyang Li, Sheng Chih Jin, Jungmin Choi, Francesc Lopez-Giraldez, Dedeepya Vaka, Annie Poon, Catherine Chu, Richard Lao, Melek Balamir, Irina Movsesyan, Mary J. Malloy, Hongyu Zhao, Pui-Yan Kwok, John P. Kane, Richard P. Lifton, Clive R. Pullinger

**Affiliations:** 1 Department of Genetics, Yale University School of Medicine, New Haven, CT, USA; 2 Cardiovascular Research Institute, University of California, San Francisco, CA, USA; 3 Department of Cardiology, The Guangzhou Eighth People's Hospital, Guangzhou Medical University, Guangzhou, China; 4 Department of Biostatistics, Yale School of Public Health, New Haven, CT, USA; 5 Department of Genetics, Washington University School of Medicine, St. Louis, MO, USA; 6 Department of Biomedical Sciences, Korea University College of Medicine, Seoul, Korea; 7 Institute for Human Genetics, University of California, San Francisco, CA, USA; 8 Department of Internal Medicine, Istanbul University, Istanbul, Turkey; 9 Department of Medicine, University of California, San Francisco, CA, USA; 10 Department of Pediatrics, University of California, San Francisco, CA, USA; 11 Department of Dermatology, University of California, San Francisco, CA, USA; 12 Department of Biochemistry and Biophysics, University of California, San Francisco, CA, USA; 13 Physiological Nursing, University of California, San Francisco, CA, USA

**Keywords:** dyslipidemia, genetics, HDL, LDL, lipoproteins, prebeta-1 HDL, reverse cholesterol transport, triglycerides, CHD, coronary heart disease, CNV, copy number variant, GO, gene ontology, GRA, Genomic Resource in Arteriosclerosis, PLTP, phospholipid transfer protein, RCT, reverse cholesterol transport, SR-B1, scavenger receptor class B, type 1, TC, total cholesterol, TG, triglyceride

## Abstract

Low levels of high density lipoprotein-cholesterol (HDL-C) are associated with an elevated risk of arteriosclerotic coronary heart disease. Heritability of HDL-C levels is high. In this research discovery study, we used whole-exome sequencing to identify damaging gene variants that may play significant roles in determining HDL-C levels. We studied 204 individuals with a mean HDL-C level of 27.8 ± 6.4 mg/dl (range: 4–36 mg/dl). Data were analyzed by statistical gene burden testing and by filtering against candidate gene lists. We found 120 occurrences of probably damaging variants (116 heterozygous; four homozygous) among 45 of 104 recognized HDL candidate genes. Those with the highest prevalence of damaging variants were *ABCA1* (n = 20), *STAB1* (n = 9), *OSBPL1A* (n = 8), *CPS1* (n = 8), *CD36* (n = 7), *LRP1* (n = 6), *ABCA8* (n = 6), *GOT2* (n = 5), *AMPD3* (n = 5), *WWOX* (n = 4), and *IRS1* (n = 4). Binomial analysis for damaging missense or loss-of-function variants identified the *ABCA1* and *LDLR* genes at genome-wide significance. In conclusion, whole-exome sequencing of individuals with low HDL-C showed the burden of damaging rare variants in the *ABCA1* and *LDLR* genes is particularly high and revealed numerous occurrences in HDL candidate genes, including many genes identified in genome-wide association study reports. Many of these genes are involved in cancer biology, which accords with epidemiologic findings of the association of HDL deficiency with increased risk of cancer, thus presenting a new area of interest in HDL genomics.

Clinical arteriosclerotic coronary heart disease (CHD) is a multifactorial disorder. Circulating lipid and lipoprotein levels, notably, triglycerides (TGs), low density lipoproteins (LDLs), and high density lipoproteins (HDLs), are independent risk factors, and other components such as TG-rich remnants and prebeta-1 HDL play important roles too. Here we report the results of a research discovery genomic study designed to understand better the genetic causes of inherited hypoalphalipoproteinemia, low levels of plasma HDL-cholesterol (HDL-C). Over forty years ago, epidemiologic studies established that low levels of HDL-C are independently associated with an elevated risk of CHD and carotid disease (1, 2, 3, 4). Those participants in the Framingham Heart Study with HDL-C levels below 35 mg/dl had eight times the prevalence of CHD compared to those with levels above 65 mg/dl (4). Other studies, including the Lipid Research Clinics Prevalence Mortality Follow-up Study, the Coronary Primary Prevention Trial, and the Multiple Risk Factor Intervention Trial (MRFIT) study, supported these findings (5). The mechanisms underlying the antiatherogenic properties of HDL-C are not fully understood. That reverse cholesterol transport (RCT) (6) is likely a major contributor here is inferred by studies that have shown cholesterol efflux capacity to be associated with atherosclerotic cardiovascular disease risk (7). It is now appreciated that the level of HDL-C itself is not an indication of the precise number of HDL particles, their functionality, the distribution of the numerous HDL subspecies, or the rate of RCT (8). Most cells, macrophages especially, efflux cholesterol to the RCT retrieval pathway to maintain cholesterol homeostasis. This process involves the ATP-binding cassette transporters ABCA1, ABCG1, and the scavenger receptor scavenger receptor class B, type 1 (9).

HDL also has a signaling role that is important to endothelial and platelet function, lymphocyte trafficking, and angiogenesis that involves HDL-bound sphingosine 1-phosphate (10, 11, 12). Other functions of HDL are emerging, including its protective role in inflammation (13) and its antioxidant properties (14). The extent to which a low level of HDL-C, resulting from known genetic causes, is a direct risk factor for atherosclerotic disease has become controversial recently with some authors doubting the genetic link (15, 16), while others have challenged this interpretation (17).

It has been long established that the heritability (H^2^) of HDL-C concentration in plasma is high, at about 0.67 (18, 19). Thus, it was considered that genetic variation that resulted in a decreased level of HDL-C might be a risk factor for CHD. Since that time, much effort has been undertaken to discover the underlying genetic variations that associate with HDL-C levels. Studies in both humans and animals initially revealed several genes that contribute to the variance in levels of HDL-C. These include the apolipoprotein A-1 gene (*APOA1*), *ABCA1*, lecithin cholesterol acyltransferase (*LCAT*), phospholipid transfer protein (*PLTP*), cholesteryl ester transfer protein (*CETP*), and hepatic lipase (*LIPC*) (20). Additional genes more recently found to be associated with HDL-C are procollagen C-endopeptidase enhancer 2 (*PCOLCE2*) (21) and WW domain–containing oxidoreductase (*WWOX*) (22). Genome-wide association studies (GWASs) in large populations have now revealed numerous additional candidates, though it is important to point out that the effect sizes of many individual gene variants are often small (23, 24, 25, 26, 27). Often, mutations in genes such as lipoprotein lipase (*LPL*), for example, that result in a sizable elevation of TGs are associated also with decreased HDL-C. Much of the lowering of HDL-C here is due to the well-known entropically driven transfer of cholesteryl esters, facilitated by CETP, from the core of HDL particles to TG-rich lipoproteins (28). This transfer manifests as a hyperbolic inverse relationship between levels of TG and HDL-C.

We performed whole-exome sequencing on 204 individuals with low levels of HDL-C. These were selected from 21,639 individuals in the UCSF Genomic Resource in Arteriosclerosis (GRA) (29, 30) for whom lipid panel data, clinical and demographic data, and DNA were available. Because, as it has been pointed out (17), a significant number (∼40%) of those with low levels of HDL-C have secondary causes, most notably hypertriglyceridemia, we selected participants taking into account the known inverse hyperbolic relationship between TG and HDL-C (28).

The sequencing data were analyzed by two separate approaches. Firstly, statistical gene burden testing was used to identify potentially causal rare variants, particularly from novel genes. Secondly, after preliminary filtering to identify rare coding variants, we filtered against two lists of candidate genes. The first list consisted of 594 lipid metabolism related genes, and this included all 23 lipid-related genes sequenced by Geller and colleagues in their study of subjects with low HDL-C (17). Our list also included all the gene loci investigated in a similar recent resequencing study (31). The other list was a subset from the first list and comprised 104 genes well established as being involved in HDL metabolism or associated robustly with levels of HDL-C in GWASs. We then used 10 deleteriousness assessment tools and the ClinVar public archive to determine the degree of probability that a particular variant was functionally damaging and therefore likely to be disease causing.

Our aim in these studies was to provide a valuable resource for future cardiovascular research by compiling a list of rare gene variants that have a high likelihood of being functionally damaging and in many cases clearly pathogenic. We believe this study is unique in applying exome sequencing specifically to a cohort with HDL deficiency. Others have used targeted sequenced of limited numbers of candidate genes, and we have compared our findings to these previous studies (17, 31).

## Materials and methods

### Study participants

A total of 204 individuals (70 women and 134 men) who had been recruited into the GRA (29, 30) were included in this study. Because this was a discovery study, no formal power calculation was done. At the time blood samples were collected, none of the individuals analyzed in this study were taking a lipid-altering medication. They were selected based on a plasma level of HDL-C below the 10th percentile of the GRA population, after adjusting for sex, and plasma level of TG based on the known hyperbolic inverse relationship between levels of HDL-C and TG (28). Briefly, using data on a total of 4,140 GRA subjects, we derived a hyperbolic 10th percentile isopleth function that takes the form y = m1 + ((m2∗m3)/(m2 + x)), where y is HDL-C, x is TG, and m1, m2, and m3 are constants. Participants completed a questionnaire to document medical history, clinically important lifestyle factors, and family history. Many of those included in this study attended a tertiary lipid clinic and had other dyslipidemias. Study participants were subdivided into four groups based on subphenotypes of dyslipidemia. We used a cutoff for defining hypertriglyceridemia as recommended by the National Cholesterol Education Program Adult Treatment Panel III (1); TG ≥150 mg/dl. Participants were considered to have high LDL-C if the value was ≥160 mg/dl, except in the case of four participants under 18 years old where the 90th percentile sex- and age-adjusted values were used (32). There were five kinships included among the 204 participants. There were two sib pairs, one mother daughter pair, and two father son pairs. All study participants gave written informed consent prior to their enrollment in the study, which adhered to the World Medical Association Declaration of Helsinki and was approved by the UCSF Institutional Review Board as part of the UCSF Human Research Protection Program. Children were included with parental consent.

### DNA preparation and biochemical analyses

Blood samples were collected, after an overnight fast, in tubes containing 0.1% EDTA. Genomic DNA was extracted using the Wizard purification kit (Qiagen, Germantown, MD). Plasma was obtained after centrifugation at 3,000 rpm for 20 min at 4°C. Levels in plasma of total cholesterol (TC), HDL-C, and TG were measured using an automated chemical analyzer (COBAS Chemistry analyzer) as previously described (29, 33). HDL-C was determined after precipitation of apolipoprotein-B-containing lipoproteins with dextran sulfate and magnesium (34). Levels of LDL-C were calculated using the Friedewald method (35) when TG levels were below 400 mg/dl. For some participants, including five with TG concentrations above 400 mg/dl, LDL-C was determined by sequential ultracentrifugation. Levels in plasma of prebeta HDL, measured as apoA1 protein content, were determined as previously described (36) .

### Whole-exome sequencing

Genomic DNA samples were sequenced at the Yale Center for Genome Analysis (n = 192) or at the UCSF Genomics CORE (n = 12) as previously described (37, 38).

Samples sequenced at Yale were exon-captured using IDt xGen target capture kit followed by 99 base-paired-end sequencing on the Illumina platform.

Samples sequenced at the UCSF were exon-captured by Roche NimbleGen SeqCap EZ library probe, and the captured libraries were sequenced on the HiSeq2500. Processing of image files was performed using a standard protocol. Raw image files were analyzed and converted to base calls by real-time analysis using the recommended default settings. Real-time analysis output base call files (∗.bcl) were converted to FASTQ files with consensus assessment of sequence and variation using bcl2fastq pipeline.

### Quality control, read alignment, and variant calling

Blue Collar Bioinformatics (Bcbio v1.0.0) was used to run the QC pipeline. We used Burrows-Wheeler Aligner (39) (BWA v0.7.15) paired-end mode to map reads to the human reference genome (hg19). BAM files obtained were used in subsequent steps. Picard (http://broadinstitute.github.io/picard/) (v2.5.0) was used to sort mapped reads into coordinate order and to ensure all mate-pair information was properly updated. Picard was also used to mark duplicate and low-quality reads, defined as those with low mapping quality score, that were unpaired or unmapped, or that failed a platform/vendor quality check. Five callers were then used for variant calling: Genome Analysis Tool Kit (40) (GATK v2015.1.1-3.4.46-0-ga8e1d99), GATK Haplotype Caller (v2015.1.1-3.4.46-0-ga8e1d99), Freebayes (41) (v1.0.2.29), Samtools (42) (v1.3.1), and VarDict (43) (v2016.02.10).

For post-variant calling analysis, filtered call sets were concatenated to create a nonredundant variant list. Synonymous, intronic, intergenic, and UTR variants were removed. Variants fulfilling the following criteria were kept: a quality score (QUAL) >40; a read depth (DP) ≥20; a genotype quality (GQ) of ≥60 if called by GATK, GATK haplotype caller, or Freebayes; variants with >1 caller. A random selection of 12 with only 1 caller were all shown to be artifacts by Sanger sequencing. Annovar (44) (version date: 2/1/2016) was then used to functionally annotate variants using the ClinVar (45) database, the Genome Aggregation Database (46) (gnomAD), the Human Non-Synonymous SNPs and Their Functional Predictions Database (47) (dbNSFP; v3.3), the Single Nucleotide Polymorphism Database (dbSNP; v147), the InterVar prediction program (48), the Mendelian Clinically Applicable Pathogenicity Score (49) (M-CAP), and the REVEL method (50). Annovar output was filtered further based on a list of common gene variants provided with the Ingenuity Variant Analysis suite. The top 50th percent of these were removed from our call set. We then filtered by gnomAD minor allele frequency (MAF). MAF cutoff threshold of 0.01 was used to identify heterozygous variants with 0.05 for the homozygous variants. For platform-specific variants, we removed those found in more than 40% of the samples. This remaining variant set was used for the “candidate gene analysis” described in the following.

For binomial analysis, variants were called using GATK Haplotype Caller and annotated using Annovar. High-quality variants which passed GATK variant quality score calibration with a read depth (DP) ≥8, a genotype quality score (GQ) ≥20, a mapping quality (MQ) ≥40, at least three supporting reads, and not falling in the low-complexity regions were kept (51). Rare variants with MAF ≤1E-05, 1E-04 or 1E-03 in ExAC, 1000 Genome, and NHLBI Exome Sequencing Project databases were examined. Damaging variants including the loss-of-function (LoF) variants and damaging missense (D-Mis) variants were considered for the analysis. LoF variants are defined as stop-gain, stop-loss, frameshift insertions/deletions, splice site disruption, and start-loss. D-Mis variants are nonsynonymous variants predicted as deleterious by MetaSVM (52).

### Final variant assessment

#### Candidate gene analysis

For the candidate gene analysis approach, we evaluated the NGS data after the post-variant calling analysis to determine the degree to which individual variants were causative, firstly in 594 candidate genes including those in lipid metabolism pathways plus GWAS hits (supplemental Table S1). This is an expanded list of genes that we previously reported (53). We also filtered using a subset of 104 of these genes that have been associated with plasma levels of HDL-C (24, 25, 26, 54) or that are empirically related (supplemental Table S2). In both cases, we separately evaluated the data for heterozygous variants and for homozygous variants. Rare variants for those with elevated either LDL-C or TG were separately also filtered against lists of genes associated with levels of LDL or TG as appropriate (24, 25).

Because this was a research discovery study, and we were more interested to look at the overall deleterious gene burden in this cohort with low HDL-C, and it was not designed to provide individual clinical assessments, we did not strictly follow the American College of Medical Genetics (ACMG) guidelines (55). Rather, we developed an overall damaging prediction based on 10 separate variant impact prediction tools and used an overall 80th percentile cutoff to retain deleterious variants. Those between the 50th and 80th percentiles were considered to be of undetermined significance and the rest (<50th percentile) likely benign. Of 66 variants with a “pathogenic” entry in ClinVar, by this approach, we classified only six as likely benign. Those variants with a damaging prediction over the 50th percentile and an unequivocal ClinVar pathogenic entry were considered pathogenic. The 10 algorithms we used were SIFT, PolyPhen (HVAR), MutationTaster, MutationAssessor, FATHMM, PROVEAN, METASVM, MetaLR, MCAP, and FATHMM-MKL.

#### Binomial analysis

A one-tailed binomial test was used to examine the enrichment of an observed number of damaging dominant variants in each gene in comparison with expectation as described before (37). The expected number of variants was calculated based on de novo mutability through the following formula:

$$Expected\;Gene_{i}=N\times \frac{Mutability_{i}}{\sum_{Genes}Mutability}$$

where ‘*i*’ denotes the ‘ith’ gene and ‘*N*’ denotes the total number of damaging dominant variants. The de novo mutability for each type of variant in each gene was calculated based on trinucleotide contexts as described by Samocha *et al.* (56) and was adjusted by base-pair coverage of 204 exome sequencing data in our cohort.

#### PANTHER analysis

For genes with recessive genotypes at MAF of ≤0.001, we used PANTHER (Protein Analysis Through Evolutionary Relationships) gene ontology analysis (57) to functionally classify the genes. We used Fisher’s exact test with false discovery rate correction to compare our list with the PANTHER human database.

#### Copy number variant analysis

Copy number variations were called using XHMM (eXome-Hidden Markov Model) software (58). GATK DepthOfCoverage was first used to calculate mean read coverage from the aligned sequencing file. The output data were then normalized by removing the variance component with variance >70%, and a z-score was calculated. Afterward, the hidden Markov-based model called copy number variants (CNVs) and calculated the quality scores. Only high-quality CNVs spanning at least three exons and with a quality score ≥90 were kept and visually inspected. The remaining CNVs were further annotated with frequencies in 1000 Genome and DECIPHER databases. Only rare CNVs with a frequency ≤1 × 10^−3^ in 1000 Genome and DECIPHER as well as an in-cohort frequency ≤10% were kept.

#### Other statistical analysis

Lipid, lipoprotein, demographic, and clinical data were analyzed using PASW Statistics for Apple Macintosh (IBM Corp., New York, NY). Continuous variables were checked for normality, and those with skewed distributions mathematically transformed prior to testing. Body mass index (BMI), TC, and TG were log-transformed and LDL-C square root transformed. *P*-values were calculated using an unpaired *t*-test for parametric variables and using Fisher’s exact test for categorical variables.

## Results

Clinical and other characteristics of the study participants are presented in Table 1. The study subjects were selected because they had plasma HDL-C below the 10th percentile for the GRA population. The mean level of HDL-C was 27.8 ± 6.4 mg/dl (range: 4–36 mg/dl) (Table 1) and is substantially lower than the mean value of 58.9 ± 19.9 mg/dl that we reported recently for a control cohort (59). There were a considerable number of participants (124; 60.8%) who presented with an additional dyslipidemia. Plasma levels of LDL-C and TG were used to allocate each participant to one of four clinically meaningful groups (Table 2) that emphasize these other dyslipidemias. These are: 1 Isolated Low HDL-C; 2 Low HDL-C plus high LDL-C; 3 Low HDL-C plus high TG; 4 Low HDL-C plus combined hyperlipidemia. There were 80 participants (39.2%) in group 1, 23 (11.3%) in group 2, 77 (37.7%) in group 3, and 24 (11.8%) in group 4. With respect to differences with group 1, there were significantly more females in group 4. HDL-C levels were lower in group 3, and TC higher in groups 2, 3, and 4. Also, the mean age in group 2 was significantly lower. Levels of prebeta HDL were significantly higher in the two groups with high TG (Table 2), consistent with our recent study (59) in which the cohort was stratified according to the same dyslipidemia criteria.

**Table 1:** Clinical and demographic characteristics of the study participants

Variable	Study Participants			*P*^b^
	All	Female	Male	
	(n = 204)	(n = 70)	(n = 134)	
Age (years)	50.0 ± 15.7	51.9 ± 15.7	49.1 ± 15.6	0.215
Age range	8–86	8–86	8–80	
Sex, female (%)	34.3			
BMI (kg/m^2^)	28.6 ± 5.8	29.2 ± 7.0	28.3 ± 5.2	
CAD (%)	8.6	2.9	11.6	0.059
MI (%)	5.5	0.0	8.3	0.017
Type 2 diabetes (%)	5.9	8.6	4.5	0.348
Hypertension (%)	28.7	29.4	28.3	0.501
Smoker (current) (%)	8.6	11.9	6.9	0.284
Ethnicity^a^ (%)				0.007
White European	76.5	64.3	82.8	
Mexican	10.8	18.6	6.7	
East Asian	3.9	1.4	5.2	
Indian	2.9	4.3	2.2	
African American	1.5	2.9	0.7	
Mixed, other	4.4	8.6	2.2	
Total cholesterol	192 ± 94	206 ± 55	185 ± 108	0.006
Triglycerides	221 ± 715	192 ± 121	236 ± 879	0.202
LDL-cholesterol	128 ± 55	141 ± 53	121 ± 56	0.015
HDL-cholesterol	27.8 ± 6.4	29.6 ± 5.4	26.9 ± 6.7	0.005
HDL-C median	29.0	31.0	28.0	
HDL-C range	(4–36)	(15–36)	(4–36)	

BMI, body mass index (kg/m^2^); CAD, coronary artery disease; MI, myocardial infarction.

Mean values for age, BMI, and lipids are ± SD.

a Self-defined ethnicity.

b *P*-value between females and males. *P*-values were calculated by *t*-test for parametric variables and by Fisher’s exact test for categorical variables. BMI, total cholesterol, and triglycerides were log-transformed, and LDL-cholesterol square root transformed, prior to testing.

**Table 2:** Patients with low-HDL cholesterol: subphenotype groups

Subphenotype		n	Age	Female %	HDL-C	TC	LDL-C^d^	TG^d^	Prebeta HDL
Group									
1	Isolated Low HDL-C	80	51.8 ± 17.3	27.5	30.3 ± 5.5	150 ± 34	103 ± 31	95 ± 33	3.53 ± 1.27
2	Low HDL-C plus high LDL-C	23	43.4 ± 15.6^a^	39.1	31.0 ± 4.4	259 ± 76^c^	210 ± 76	105 ± 21	3.86 ± 1.09
3	Low HDL-C plus high TG	77	49.1 ± 14.4	31.2	24.3 ± 6.1^c^	194 ± 123^c^	110 ± 26	375 ± 1,148	4.88 ± 1.12^c^
4	Low HDL-C plus combined hyperlipidemia	24	51.4 ± 15.9	62.5^b^	27.7 ± 6.6	264 ± 50^c^	189 ± 45	256 ± 77	5.40 ± 1.20^c^

TC, total cholesterol; TG, triglyceride: all lipid measurements in mg/dl.

Values for age, lipids, and lipoproteins are ± SD. Prebeta HDL values are mg/dl of apoAI. *P* values were calculated by *t*-test with respect to differences with the isolated low HDL-C group. Fisher exact test was used for sex differences. Total cholesterol values were log-transformed prior to testing.

a *P* = 0.038.

b *P* = 0.003.

c *P* < 0.001.

d These variables were not tested as they were used as criteria in group selection.

The participants, as self-assessed, were primarily of white European ancestry (76.5%) but included those of Hispanic, East Asian, South Asian, and African-American descent. The self-assessed ethnicity percentages agree very closely with those determined by principal component analysis (Data not shown). Table 1 shows that there were significant differences in ethnicity between males and females, with more males being European and nearly three times as many females being of Hispanic ancestry. Study participants were generally overweight, and 8.6% had CHD, with more males being affected than females. Few had diabetes though a considerable number suffered from hypertension, and there were few smokers.

### Candidate gene analysis

Among the whole cohort of 204 participants, after filtering against 594 lipid metabolism candidate genes (supplemental Table S1), we detected a total of 460 potentially damaging heterozygous variants (40 of which were considered to be pathogenic) and 10 that were homozygous (one of which was known to be pathogenic; *LPL* p.G215E) (Table 3). There were 591 individual occurrences of these variants distributed among 192 participants. All these variants are listed in supplemental Table S3. The candidate genes with the most frequent occurrences of potentially damaging variants were *ABCA1* (n = 20), *LDLR* (n = 15), *LPA* (n = 14), *CYP1A1* (n = 12), *ABCC1* (n = 11), *PDIA2* (n = 11), *ABCC3* (n = 9), *STAB1* (n = 9), *ABCC2* (n = 8), *ACACB* (n = 8), *APOBEC1* (n = 8), *CPS1* (n = 8), *OSBPL1A* (n = 8), *BRCA2* (n = 7), *CD36* (n = 7), *PCCB* (n = 7), *ABCA8* (n = 6), *AGPAT2* (n = 6), *APOB* (n = 6), *BCMO1* (n = 6), *CREBBP* (n = 6), *LRP1* (n = 6), and *MYL5* (n = 6). A selection of rare variants, 48 in total, were all confirmed to be real by Sanger sequencing.

**Table 3:** Characterization of variants after post-variant calling analysis

Variation	Probably Benign or Undetermined	Probably Damaging	Pathogenic^b^
Heterozygous (all lipid genes)	2,622	**420**	**40**
Heterozygous (only HDL genes)	519	111	9
Homozygous (all lipid genes)	110	**9**	**1**
Homozygous (only HDL genes)	29	4	1
Homozygous (other genes; MAF <0.05)	250	**49**	**6**
Missense	2,193	**378**	**33**
Splice site	384	**18**	**1**
Frameshift/stop gained	-	**80**	**10**
Other insertions/deletions etc^a^	374	**2**	**3**

a Other variants: 5′UTR premature start codon gain; noncoding transcript exon variant; start lost.

b Damaging score and ClinVar pathogenic entry. Probably damaging and Pathogenic variants for all lipid genes are in bold.

We detected a total of 34 rare *ABCA1* variants among 38 (18.6%) of the 204 individuals studied, with 20 of the variants considered to be potentially damaging or pathogenic. These 20 mutations (one person carried two *ABCA1* variants) are listed in Table 4, which includes lipid and lipoprotein measurements. Six of the variants reported here are novel. Two of these were frameshifts, and one was a nonsense mutation. There was a ClinVar entry on seven of the others. Four ClinVar entries stated they were “likely benign,” and one was of “uncertain significance,” despite their high damaging prediction rating. Those participants with deleterious *ABCA1* variants, when compared to noncarriers, had a lower level of LDL-C (103 ± 32 mg/dl vs. 131 ± 57; *P* = 0.040). There were no other significant differences in lipid or lipoprotein measurements, including prebeta HDL (4.20 ± 1.36 mg/dl vs. 4.30 ± 1.39; *P* = 0.802).

**Table 4:** Twenty rare heterozygous missense, nonsense, and frameshift mutations in *ABCA1*

Ethnicity	Sex	Genomic Information								Clinical Lipid Data				
		Chr:Pos:Ref:Alt	AA Change	ABCA1 Domain	gnomAD	ID	ClinVar Significance	DP^a^	Prediction	HDL-C	LDL-C	TG	TC	Prebeta HDL
European	M	9-107665929-T>C	L11P	IH1	4.84E-06	rs777372679	.	10	Probably damaging	7	137	159	165	ND
European	M	9-107646756-C>T	P85L	ECD1	1.40E-03	rs145183203	Likely benign	8.5	Probably damaging	35	103	76	153	4.14
European	F	9-107602623-A>G	K331E	ECD1	-	-	.	7.5	Probably damaging	27	107	209	176	6.14
Indian	M	9-107599797-G>A	R369H	ECD1	2.44E-05	rs370223805	.	9.5	Probably damaging	28	112	96	153	4.65
European	F	9-107599263-C>T	R437W	ECD1	4.87E-05	rs150448790	.	9.5	Probably damaging	35	110	91	156	4.2
European	M	9-107594878-C>T	R496W	ECD1	6.00E-04	rs147675550	Likely benign	7	Probably damaging	23	60	200	113	4.42
		9-107574868-G>A	G1346E	IH3	1.00E-04	rs762770081	Likely pathogenic	9.5	Pathogenic					
European	M	9-107593329-G>A	W590X	ECD1	-	-	.	-	Probably damaging	22	83	187	142	5.75
European	M	9-107593272-C>T	T609M	ECD1	3.25E-05	rs755276277	.	9	Probably damaging	23	61	44	88	1.33
East Asian	M	9-107587972-T>C	V845A	TMD1	3.25E-05	rs541344598	.	8	Probably damaging	36	125	77	177	3.53
European	M	9-107584879-dupTACC	R909fs	NBD1	-	-	.	-	Probably damaging	26	134	84	177	4.95
European	M	9-107583758-C>T	T953I	NBD1	-	-	.	9.5	Probably damaging	23	127	177	185	4.16
European	M	9-107578515-G>T	G1216V	R1	4.47E-05	rs562403512	.	9.5	Probably damaging	25	97	65	132	ND
Hispanic	F	9-107578437-C>T	T1242M	R1	2.03E-05	rs144923927	.	10	Probably damaging	15	36	750	214	ND
European	M	9-107574881-C>T	R1342W	IH3	1.62E-05	rs760786920	Uncertain significance	9.5	Probably damaging	4	136	275	200	ND
Mexican	F	9-107571799-C>T	L1408F	ECD2	4.00E-04	rs201879964	Likely benign	6.5	Probably damaging	35	126	195	200	ND
European	M	9-107568536-insT	L1484fs	ECD2	-	-	.	-	Probably damaging	7	121	260	177	5.79
European	M	9-107566964-C>T	T1501	ECD2	-	-	.	9.5	Probably damaging	28	73	149	130	2.22
European	M	9-107560784-G>A	R1680Q	TMD2	3.00E-04	rs150125857	Likely benign	9.5	Probably damaging	33	58	45	100	2.85
European	F	9-107556776-A>C	N1800H	TMD2	3.00E-04	rs146292819	Pathogenic	9	Pathogenic	27	145	119	196	4.71

ECD1/2, extracellular domains 1 and 2; IH1/3, intracellular helices 1 and 3; NBD1, nucleotide-binding domain 1; R1, regulatory domain 1; TMD1/2, transmembrane domains 1 and 2.

a Damaging prediction: number of damaging predictions out of 10 separate variant impact prediction tools. Lipid values in mg/dl (prebeta HDL measured as apoA1 content).

Participants with deleterious variants among one or more of the *ABCC1*, *ABCC2*, or *ABCC3* transporter genes (supplemental Table S3), when compared to noncarriers, had no statistically significant differences in lipid or lipoprotein measurements, including prebeta HDL (4.19 ± 1.53 mg/dl vs. 4.31 ± 1.37; *P* = 0.689).

**Fig. 1:**
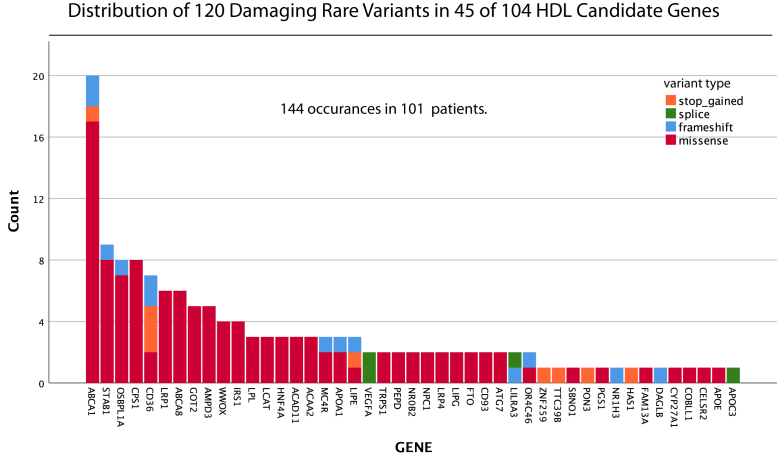
Histogram showing the distribution among participants with low HDL cholesterol of 120 probably damaging rare HDL candidate gene variants. These variants were found within 45 of the 104 candidate genes. HDL, high density lipoprotein.

Among the 104 HDL-C candidate genes (supplemental Table S2), there was a total of 120 probably damaging variants (Fig. 1), 116 of which were heterozygous (eight considered to be pathogenic) and four were homozygous (one pathogenic). There were 110 participants who had at least one potentially damaging HDL candidate gene variant, 31 of whom had 2 and 10 had 3 each. Of these 110 individuals, 46 were among group 1 (Isolated low HDL group; Table 2), eight among group 2 (Low HDL-C plus high LDL-C), 45 among group 3 (Low HDL-C plus high TG), and 11 among group 4 (Low HDL-C plus combined hyperlipidemia). The percentages in these groups were 57.5%, 34.8%, 58.4%, and 45.8%, respectively. Hence, the frequencies were lower in the two groups with elevated levels of LDL-C (groups 2 and 4). This perhaps reflects the impact of the high frequency of damaging *LDLR* mutations on lowering HDL-C among these two groups.

The 11 HDL genes with the highest occurrence of damaging variants were *ABCA1* (n = 20), *STAB1* (n = 9), *OSBPL1A* (n = 8), *CPS1* (n = 8), *CD36* (n = 7), *LRP1* (n = 6), and *ABCA8* (n = 6), *GOT2* (n = 5), *AMPD3* (n = 5), *WWOX* (n = 4), and *IRS1* (n = 4) (Fig. 1). All the damaging variants among HDL candidate genes are listed in supplemental Table S4. Three probably damaging rare variants were found in the *APOA1* gene, which codes for the major protein of HDL, and two in *LCAT* (Table 5). None have been reported in ClinVar. We noticed also that one person was homozygous for a possibly damaging *LCAT* SNP (p.Ser232Thr; no ClinVar entry), but the frequency here (gnomAD MAF 0.0176) is above the inclusion cutoff we used for rare heterozygous variants and the damaging prediction score was borderline at the 80th percentile cutoff. However, the frequency was considerably higher in our cohort (MAF 0.0466), with 17 heterozygotes and 1 homozygote. Data relating to this *LCAT* SNP are included in Table 5 (but excluded from Fig. 1). Two individuals were homozygous for *LPL* mutations, p.Asp36Asn (TG 295 mg/dl) and p.Gly215Glu (TG 406 mg/dl). The first of these has an equivocal entry in ClinVar, and the second is listed as pathogenic.

**Table 5:** Likely causal mutations in *APOA1* and *LCAT*

N	Ethnicity	Genomic Information								Clinical Lipid Data			
		Gene	Chr:Pos:Ref:Alt	AA Change	gnomAD	ClinVar Significance	ClinVar Disease	DP^a^	Prediction	HDL-C	LDL-C	TG	TC
1	Caucasian	*APOA1*	11-116706768-T>G	p.L187R	-	Not reported	.	9.5	Probably damaging	7	208	383	267
1	Caucasian	*APOA1*	11-116707739-T>G	p.S60A	3.00E-04	Not reported	.	7	Probably damaging	23	127	177	185
1	Caucasian	*APOA1*	11-116707831-dupC	p.Q29fs	1.63E-05	Not reported	.	-	Probably damaging	31	348	120	395
1	Caucasian	*LCAT*	16-67976824-C>T	p.R123C	8.13E-06	Not reported	.	10	Probably damaging	31	179	197	230
1	Caucasian	*LCAT*	16-67977851-G>A	p.V52M	2.76E-05	Not reported	.	8.5	Probably damaging	26	163	160	208
18	Caucasian	*LCAT*	16-67976320-T>A	p.S232T	0.0176	Not reported	.	6.5	Probably damaging	26.5^b^	114^b^	144^b^	165^b^

a Damaging prediction: number of damaging predictions out of 10 separate variant impact prediction tools. Lipid values are mg/dl.

b Mean values. The LCAT p.S232T variant was included in the table even though the MAF is above the cutoff.

Two homozygous damaging mutations (frameshift and acceptor splice mutants) were discovered in the *LILRA3* gene (leukocyte immunoglobulin–like receptor A3) in a person of Japanese ancestry. These were p.Leu131fs (rs201804218; gnomAD 0.0059) and c.86-1G>C (rs11574607; gnomAD 0.0208).

**Fig. 2:**
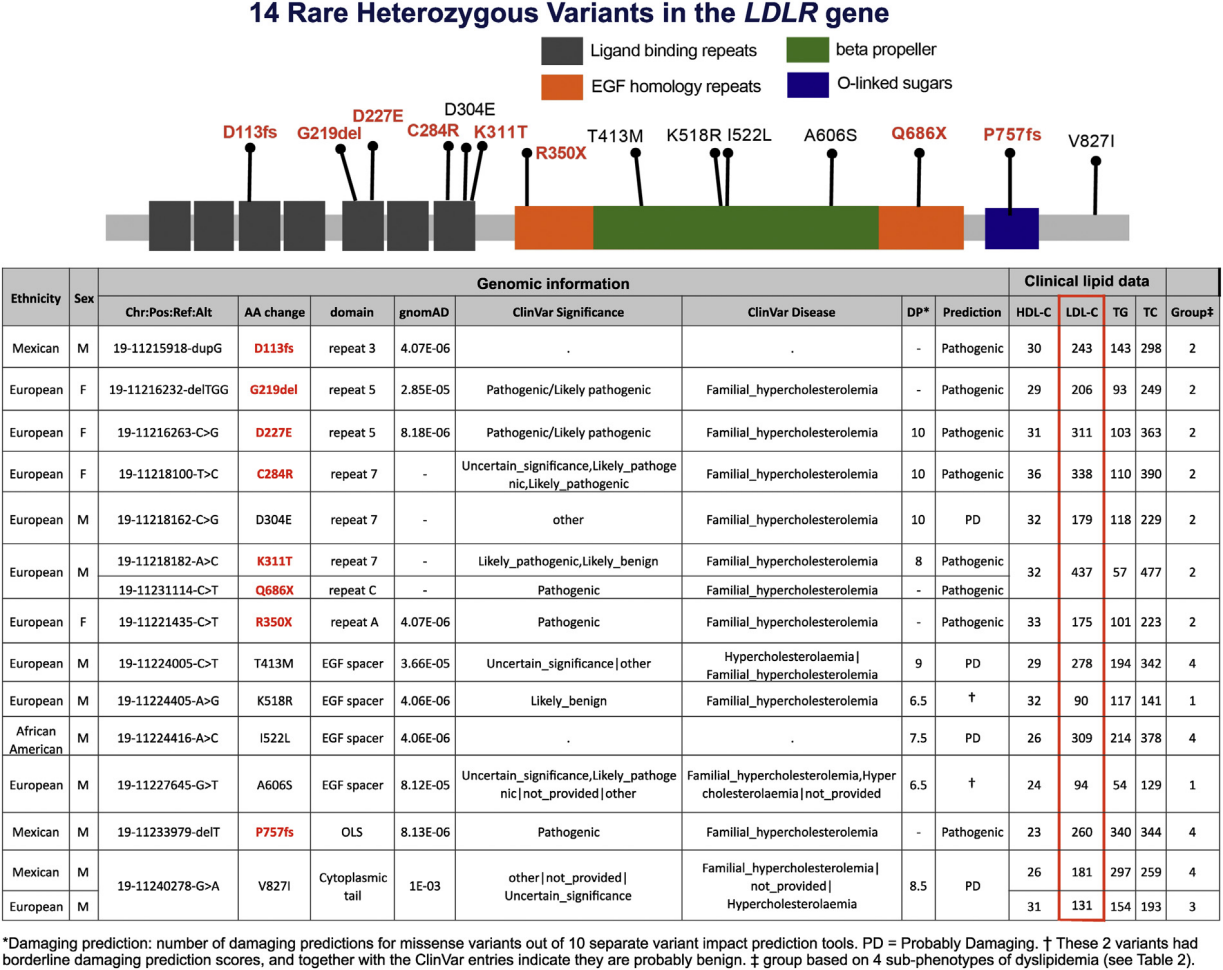
Individuals with low levels of plasma HDL-C who carry rare, potentially damaging, *LDLR* variants. Twelve of these 14 variants are pathogenic, or probably so.

We found a total of 14 different rare variants in the *LDLR* gene among 14 participants. One variant was found in two individuals, and one other carried two variants. The subjects with these variants are listed in Fig. 2 along with lipid levels. Twelve *LDLR* variants are potentially deleterious mutations, with two having borderline damaging prediction evaluations and equivocal ClinVar entries. There were two frameshift, two nonsense, and one deletion mutation. The 12 individuals with *LDLR* mutations unequivocally classified as either pathogenic or probably damaging each have an LDL-C above 160 mg/dl and are among those with subphenotypes 2 or 3 (Table 2). Those with the 12 deleterious *LDLR* variants, when compared to noncarriers, had a higher level of LDL-C (254 ± 86 mg/dl vs. 120 ± 42; *P* < 0.001) and of TC (312 ± 84 mg/dl vs. 185 ± 90; *P* < 0.001). There were no significant differences in other lipid or lipoprotein measurements, including prebeta HDL (4.54 ± 1.49 mg/dl apoA1 vs. 4.28 ± 1.39; *P* = 0.543). Among the 47 individuals with elevated LDL (Table 2; groups 2 and 4), we found when filtering against 67 LDL candidate genes (23, 24, 26) a total of 29 potentially damaging variants (all heterozygous) in 21 of these individuals in 15 genes (supplemental Fig. S1). The *LDLR* mutations were by far the most numerous with 12 occurrences (not including the two with borderline scores, equivocal ClinVar entries and normal levels of LDL-C). There were two mutations in each of *ABCG5*, *APOA1*, *HPR*, and *IRF2BP2*.

For the 101 individuals with elevated levels of TG, we filtered against 61 TG candidate genes (23, 24, 26). We found 32 potentially damaging variants in 21 genes among 31 individuals (supplemental Fig. S2). There were three *LPL* and three *LRP1* mutations and two each in the *LMF1*, *APOA1*, *GCKR*, *PPARA*, *INSR*, *PEPD*, *CYP26A1*, *ATG4C*, and *CAPN3* genes. Of note, in this respect, no damaging variants were seen in three key genes: *APOA5*, *APOC2*, or *GPIHBP1*.

Notable among the damaging mutations found in the other lipid metabolism candidate genes were those in *ABCC1*, *ABCC2*, and *ABCC3* (supplemental Table S3). The total numbers of participants with damaging mutations in these three genes were 11, 8, and 9, respectively. In total, there were 26 who carried one or more of these variants. These 26 were evenly distributed across the four dyslipidemia subphenotypes (Table 2). These variants therefore were not linked with the presence of high TG or LDL-C, only with low HDL.

As part of our post variant calling analysis, we filtered for all genes with MAF <0.05 for homozygosity of damaging variants. Of note, there were two individuals with a damaging homozygous mutation (p.Arg83Gln; rs8140287) in the *ISX* gene, which is highly and exclusively expressed throughout the intestines. The homozygous frequency of this variant is expected to be 5-fold less than seen here in this cohort. *ISX* downregulates intestinal expression of SR-BI (scavenger receptor class B, type I; *SCARB1*) (60), an HDL receptor that mediates the selective uptake of HDL-C. Two brothers were heterozygous carriers of a rare missense mutation (c.3019C>G; p.Pro1007Ala) in the Niemann-Pick type C gene (*NPC1*, an HDL-C candidate gene). In the ClinVar record for this variant (rs80358257), it is described as pathogenic and linked to Niemann-Pick type C disease.

### Binomial and PANTHER analysis results

**Fig. 3:**
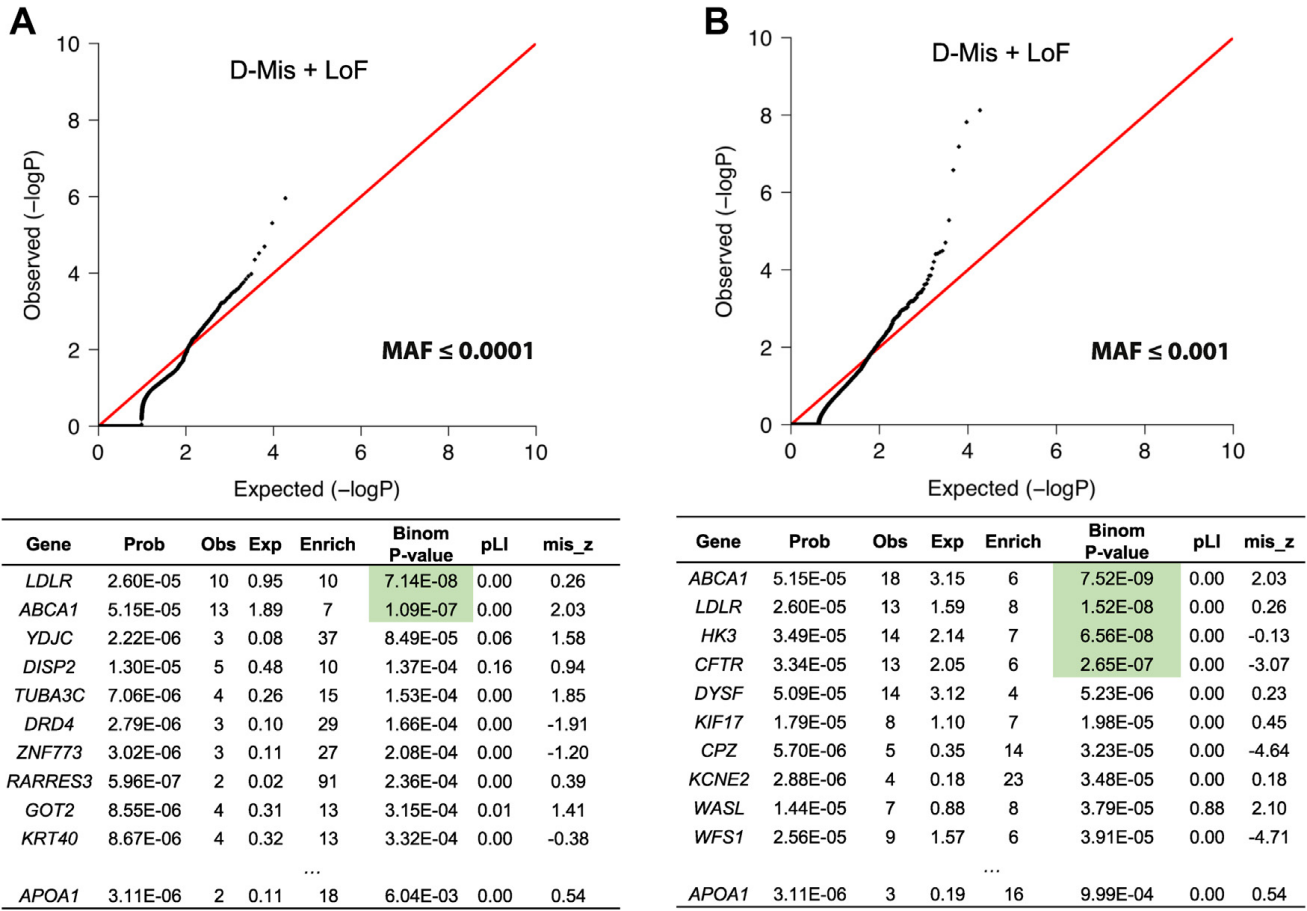
Enrichment for damaging heterozygous gene variants. Q–Q plots comparing observed versus expected *P* values in participants with low HDL-C compared to controls. Binomial analysis for D-Mis and LoF variants at MAF of ≤0.0001 (A) and ≤0.001 (B). The significance of the difference between the observed and expected number of heterozygous variants was calculated using a one-sided binomial test. *ABCA1* and *LDLR* show genome-wide threshold significance of increased burden of damaging heterozygous variants at both MAFs. In addition, *HK3* and *CFTR* are significant at an MAF of ≤0.001. LoF, loss-of-function.

Binomial analysis for rare (MAF ≤ 0.001) heterozygous D-Mis or LoF variants identified the *ABCA1*, *LDLR*, *HK3*, and *CFTR* genes with genome-wide statistical significance (Fig. 3). This was based on gene size, de novo mutability, and case-control analysis, and where there was reliable coverage in control data sets. *ABCA1* and *LDLR* are included in our set of lipid metabolism–related candidate genes (supplemental Table S1). *ABCA1* is included in our set of HDL-C candidate genes (supplemental Table S2).

Hexokinase 3 (HK3) phosphorylates intracellular glucose to produce glucose-6-phosphate, the first step in most glucose metabolism pathways. Cystic fibrosis transmembrane conductance regulator (CFTR) is also known as an ATP-binding cassette, subfamily C, member 7 (ABCC7). The results for these two genes (*HK3* and *CFTR*) are less convincing than those for *ABCA1* and *LDLR* because of the inflation in the Q–Q plot.

The results showing the functional classification of genes by PANTHER analysis of recessive D-Mis + LoF genotypes at MAF of ≤0.001 are presented in Table 6. Several metabolism-related gene ontology (GO) terms show a large magnitude of enrichment with significant *P*-values. Among the top 25 terms are five related to lipid metabolism pathways.

**Table 6:** PANTHER gene ontology analysis for 50 genes with D-Mis + LoF recessive genotypes at MAF ≤0.001

GO Biological Process Complete	# Genes in Pathway	# Genes Hit	Expected	Fold Enrichment	Raw *P*-Value	FDR	Genes
Striated muscle cell development (GO:0055002)	133	5	0.32	15.79	1.88E-05	4.95E-02	*MYO18B*, *RYR1*, *MYH11*, *TTN*, *MYH3*
Cellular response to LDL particle stimulus (GO:0071404)^a^	18^a^	3^a^	0.04^a^	69.99^a^	1.63E-05^a^	5.14E-02^a^	*LPL*, *LDLR*, *ABCA1*^a^
Response to hexose (GO:0009746)^a^	148^a^	5^a^	0.35^a^	14.19^a^	3.10E-05^a^	6.10E-02^a^	*PTK2B*, *GCKR*, *POLG*, *LPL*, *COL4A3*^a^
Response to lipoprotein particle (GO:0055094)^a^	25^a^	3^a^	0.06^a^	50.39^a^	3.96E-05^a^	6.25E-02^a^	*LPL*, *LDLR*, *ABCA1*^a^
Response to monosaccharide (GO:0034284)^a^	153^a^	5^a^	0.36^a^	13.72^a^	3.61E-05^a^	6.33E-02^a^	*PTK2B*, *GCKR*, *POLG*, *LPL*, *COL4A3*^a^
Muscle fiber development (GO:0048747)	60	4	0.14	27.99	1.61E-05	6.36E-02	*MYO18B*, *RYR1*, *MYH11*, *TTN*
Muscle cell development (GO:0055001)	146	5	0.35	14.38	2.91E-05	6.55E-02	*MYO18B*, *RYR1*, *MYH11*, *TTN*, *MYH3*
Cellular response to lipoprotein particle stimulus (GO:0071402)^a^	27^a^	3^a^	0.06^a^	46.66^a^	4.90E-05^a^	7.02E-02^a^	*LPL*, *LDLR*, *ABCA1*^a^
Small-molecule metabolic process (GO:0044281)^a^	1816^a^	15^a^	4.32^a^	3.47^a^	1.40E-05^a^	7.38E-02^a^	*ABHD1*, *IDUA*, *GCKR*, *HMGCLL1*, *LPL*, *LDLR*, *SCLY*, *PNKD*, *TM7SF2*, *ABCA1*, *MYH3*, *ENTPD2*, *SULT1A2*, *APOBEC3B*, *SLC7A4*^a^
Response to carbohydrate (GO:0009743)^a^	179^a^	5^a^	0.43^a^	11.73^a^	7.45E-05^a^	9.80E-02^a^	*PTK2B*, *GCKR*, *POLG*, *LPL*, *COL4A3*^a^
Small-molecule catabolic process (GO:0044282)^a^	419^a^	8^a^	1^a^	8.02^a^	6.84E-06^a^	1.08E-01^a^	*ABHD1*, *IDUA*, *HMGCLL1*, *SCLY*, *PNKD*, *ENTPD2*, *SULT1A2*, *APOBEC3B*^a^
Cardiac muscle fiber development (GO:0048739)	17	3	0.04	74.1	1.40E-05	1.10E-01	*MYO18B*, *MYH11*, *TTN*
Striated muscle cell differentiation (GO:0051146)	197	5	0.47	10.66	1.16E-04	1.40E-01	*MYO18B*, *RYR1*, *MYH11*, *TTN*, *MYH3*
Actomyosin structure organization (GO:0031032)	105	4	0.25	16	1.30E-04	1.47E-01	*PTK2B*, *MYH11*, *TTN*, *MYH3*
Muscle cell differentiation (GO:0042692)	239	5	0.57	8.78	2.78E-04	2.74E-01	*MYO18B*, *RYR1*, *MYH11*, *TTN*, *MYH3*
Lipid homeostasis (GO:0055088)^a^	127^a^	4^a^	0.3^a^	13.23^a^	2.64E-04^a^	2.78E-01^a^	*GCKR*, *LPL*, *LDLR*, *ABCA1*^a^
Mitochondrial DNA replication (GO:0006264)	9	2	0.02	93.32	3.00E-04	2.78E-01	*POLG*, *PRIMPOL*
Muscle contraction (GO:0006936)	247	5	0.59	8.5	3.23E-04	2.83E-01	*RYR1*, *MYH11*, *TTN*, *MYH3*, *CLCN1*
Myosin filament organization (GO:0031033)	12	2	0.03	69.99	4.94E-04	3.12E-01	*MYH11*, *TTN*
System process (GO:0003008)	1927	13	4.59	2.83	4.56E-04	3.13E-01	*RP1L1*, *SCN11A*, *RYR1*, *LDLR*, *PNKD*, *MYO7A*, *MYH11*, *COL4A3*, *CACNA2D4*, *TTN*, *OTOF*, *MYH3*, *CLCN1*
Myofibril assembly (GO:0030239)	63	3	0.15	20	5.20E-04	3.15E-01	*MYH11*, *TTN*, *MYH3*
Skeletal muscle myosin thick filament assembly (GO:0030241)	11	2	0.03	76.35	4.24E-04	3.19E-01	*MYH11*, *TTN*
Organic acid metabolic process (GO:0006082)	1,005	9	2.39	3.76	5.68E-04	3.20E-01	*ABHD1*, *IDUA*, *GCKR*, *HMGCLL1*, *LPL*, *SCLY*, *PNKD*, *SULT1A2*, *SLC7A4*
Myosin filament assembly (GO:0031034)	12	2	0.03	69.99	4.94E-04	3.25E-01	*MYH11*, *TTN*
Cardiac muscle cell development (GO:0055013)	60	3	0.14	21	4.53E-04	3.25E-01	*MYO18B*, *MYH11*, *TTN*
Cardiac cell development (GO:0055006)	65	3	0.15	19.38	5.67E-04	3.31E-01	*MYO18B*, *MYH11*, *TTN*
Striated muscle myosin thick filament assembly (GO:0071688)	11	2	0.03	76.35	4.24E-04	3.35E-01	*MYH11*, *TTN*
Response to glucose (GO:0009749)	143	4	0.34	11.75	4.10E-04	3.40E-01	*PTK2B*, *POLG*, *LPL*, *COL4A3*
Mitochondrial DNA metabolic process (GO:0032042)^a^	14^a^	2^a^	0.03^a^	59.99^a^	6.50E-04^a^	3.53E-01^a^	*POLG*, *PRIMPOL*^a^
Regulation of plasma lipoprotein particle levels (GO:0097006)^a^	72^a^	3^a^	0.17^a^	17.5^a^	7.55E-04^a^	3.61E-01^a^	*LPL*, *ABCA1*^a^

Fisher’s test was used.

a Metabolism-related terms.

### Copy number variants

Rare copy number gains and losses were examined using eXome-Hidden Markov Model (XHMM; https://atgu.mgh.harvard.edu/xhmm/) software. A total of 49 occurrences of duplications (supplemental Table S5) and 20 occurrences of deletions (supplemental Table S6) were found in the cohort. Among them, two duplications and two deletions were recurrent in two individuals. Pathway analysis using the genes that are altered in copy number did not yield any significantly over-represented GO terms (data not shown). No CNVs were found among our list of 104 HDL candidate genes (supplemental Table S2). A large, homozygous, 11,534-bp deletion, which includes the whole of exon 9, in the *MTTP* gene (microsomal triglyceride transfer protein) is a very likely cause of low HDL-C in one subject. This person has the phenotype of abetalipoproteinemia (TC 28 mg/dl; TG 6 mg/dl; LDL-C 6 mg/dl; HDL-C 22 mg/dl), a disorder caused by defects in this gene (61).

## Discussion

In this research discovery study, we aimed to gain a better understanding of the genetic basis of low levels of plasma HDL-C. Binomial analysis revealed four genes to carry potentially damaging rare variants (MAF ≤ 0.001) with genome-wide statistical significance among our cohort with low levels of plasma HDL-C. These were *ABCA1*, *LDLR*, *HK3*, and *CFTR*. PANTHER functional classification analysis revealed significant enrichment of lipid metabolism–related GO pathways. Although no CNVs were found for any genes on our candidate gene lists, a large deletion was found in the *MTTP* gene in a person with the phenotype of abetalipoproteinemia, and this is certainly the cause of his low HDL-C (22 mg/dl).

For the potentially damaging variants we discovered that were on our list of lipid metabolism–related candidate genes, the most prevalent were among *ABCA1* and *LDLR*. A total of 38 among the 204 studied carried rare *ABCA1* variants in our study (18.6%). This is somewhat lower than the 26.9% found in a recent report (17), though that cohort (n = 202) had a lower mean level of HDL-C (18 mg/dl) than our present study (27.8 ± 6.4 mg/dl; range 4–36 mg/dl). When we employed stringent cutoffs using 10 variant impact prediction tools, 19 of the 38 individuals with rare *ABCA1* variants carried potentially damaging mutations (one carried 2), that is, 9.3% of the cohort of 204.

We detected a higher prevalence of damaging, rare *LDLR* variants (12 of 204 participants) than a recent similar study of subjects with low HDL-C (4 of 202) (17). However, in that study, the mean plasma level of LDL-C was somewhat lower than here (107 mg/dl vs. 128 mg/dl). A significant percentage (23%; 47) of our cohort had elevated LDL-C (≥160 mg/dl), and all 12 with damaging *LDLR* mutations fell within this group. These 12 are characterized as having a diagnosis of familial hypercholesterolemia (FH). Among 18 kindred with FH and known *LDLR* gene deleterious mutations in our GRA collection, we have analyzed the baseline levels of LDL-C and HDL-C for a total of 128 genotyped individuals. Among these were 54 wild type, 69 heterozygotes, and five homozygotes. LDL-C values were, respectively, 127 ± 30 mg/dl (range: 63–203), 295 ± 77 mg/dl (range: 168–509), and 817 ± 102 mg/dl (range: 719–925) (ANOVA *P* < 0.001). Corresponding values for HDL-C were 54.6 ± 16.7 mg/dl (range: 25–107), 47.6 ± 15.4 mg/dl (range: 20–99), and 34.4 ± 8.0 mg/dl (range: 25–45) (ANOVA *P* = 0.005). The HDL-C values are presented as box plots in supplemental Fig. S3. These values are similar to those reported previously (62). It is clear that patients with FH tend to have a decreased level of HDL-C, but the reason for this is not clear (62), though it has been suggested recently that it may be connected to impaired RCT (63, 64).

In addition to those in *ABCA1*, there were numerous other probably damaging or pathogenic variants found among other HDL candidate genes. Notable were the nine occurrences in *STAB1*, eight in each of *OSBPL1A* and *CPS1*, and six in each of *LRP1* and *ABCA8*. *STAB1* codes for stabilin 1, a multifunctional scavenger receptor (65), and associates with HDL levels in GWASs (24, 25). A nonsense mutation in the *OSBPL1A* gene has previously been reported to be causal for low HDL-C and shown to decrease cellular cholesterol efflux capacity (66). Although its function in lipid metabolism is unclear, it is thought that the OSBPL proteins are phospholipid/sterol transporters with OSBPL1A, regulating interactions between the endoplasmic reticulum and the late endocytic compartment (66). *CPS1*, which codes for carbamoyl phosphate synthetase I, associates with HDL levels in GWASs (24, 25). It is responsible for converting ammonia to carbamyl phosphate in the liver. It is unclear how heterozygous LoF mutations can affect HDL-C levels. In GWASs, *LRP1* has been reported to associate with plasma levels of HDL-C and TG (24, 26). This gene codes for the large LDL receptor–related protein 1, which has homology to the LDLR. It is also referred to as the α2-macroglobulin receptor. *LRP1* is widely expressed, notably in the liver, adipocytes, ovary, mammary gland, fibroblasts, and central nervous system (CNS). It is a multifunctional endocytic receptor thought, pertinently here, to be involved in the clearance of chylomicron remnants by the liver (67). *ABCA8* (ATP-binding cassette, subfamily A, member 8) is widely expressed, notably in the heart, skeletal muscle, and liver and codes for a lipophilic xenobiotic transporter. It has recently been shown in mice to be responsible for the efflux of cholesterol and taurocholate across the hepatic sinusoidal membrane (68). Others have reported that ABCA8 colocalizes with ABCA1 and that mutations in *ABCA8* are associated with lower levels of plasma HDL-C (69). In addition to these findings was the presence of the *LCAT* SNP, rs4986970 (p.S232T) in 18 subjects at a frequency (MAF 0.047) here higher than in GnomAD (0.017), ExAC (0.018), or TOPMED (0.017). This SNP has been previously reported to associate with HDL levels in two Danish studies (16). The prediction tools we used did not indicate that this *LCAT* SNP was especially damaging, though five of the 10 returned a “damaging” score. It is not reported in ClinVar. One participant, of Japanese ancestry, was homozygous for two damaging mutations in the *LILRA3* gene. This gene was strongly associated with levels of HDL-C in GWASs (24, 25, 26, 54). However, results of genetic studies among a Japanese population cast doubt on the extent to which these particular *LILRA3* mutations here can be classed as causal (70).

One notable finding in these studies was the high prevalence of potentially damaging rare variants among three lipid metabolism candidate genes, all members of the ATP-binding cassette subfamily C. There were 11 occurrences with *ABCC1*, eight with *ABCC2*, and nine with *ABCC3*. In total, there were 26 participants who carried one or more of these variants. These 26 were evenly distributed across the four dyslipidemia subphenotypes, that is, they were not associated with the presence of high TG or LDL-C. These genes code for proteins previously known as multidrug resistance proteins, MRP1, MRP2, and MRP3. ABCC2 and ABCC3 proteins are also referred to as canalicular multispecific organic anion transporters, CMOAT and CMOAT2. As with *ABCC1*, these genes are expressed mainly in the liver apical canalicular membrane and act to transport conjugated anionic compounds, including conjugates of bile salts into bile. It is of further interest in this respect that there were 13 occurrences of damaging variants in *CFTR* among our study group with low HDL-C. This gene was not on our list of lipid candidate genes, and the variants were revealed by binomial analysis. However, it is also a member of ATP-binding cassette subfamily C and is also referred to as ABCC7.

Apart from the *LDLR* gene, the significance of the potentially damaging variants found among other non-HDL candidate genes is unclear. The potentially damaging variants in the *LPA*, *PDIA2*, *APOBEC1*, *BRCA2*, *PCCB*, *AGPAT2*, *CREBBP*, and *MYL5* genes are in each case fairly evenly distributed between the four different dyslipidemia subphenotypes. The numerous *CYP1A1* variants are, except for one person with high TG, found among those with isolated low HDL-C. The six participants with *APOB* mutants are among the isolated low HDL-C group, except for 1 with high LDL-C. The *ACACB* and *BCMO1* mutants are distributed among the high-TG and isolated low-HDL groups.

We tested whether the plasma levels of prebeta HDL were associated with genes with the most prevalent numbers of variants, restricting our analysis to *ABCA1*, *LDLR*, and those individuals carrying one or more of the *ABCC1*, *ABCC2*, or *ABCC3* transporters. In no case was there a difference in the level of prebeta HDL between carriers and noncarriers. Here all the deleterious *ABCA1* rare variants were heterozygous. With homozygous cases of ABCA1 deficiency (Tangier disease), almost all the apoA1in plasma is found in the form of small prebeta HDL particles (71).

Because of the ubiquity of lipid metabolism in biology and the many roles of HDL beyond lipid transport, per se, it is likely that alterations in genes with roles in HDL metabolism will have impacts broadly in human biology. A large population study revealed significant increases in cancer associated with low levels of HDL, specifically, myeloma, myeloproliferative tumors, breast, lung, and nervous system cancers (72). A critical structural or functional role for the *WWOX* gene in the CNS would account for the observation that mutations at that locus are associated with neurodevelopmental and neurodegenerative disorders, including Alzheimer disease (73). A number of the gene loci identified with low HDL levels in this study are associated with cancer. The IRS1 (insulin receptor substrate 1) protein has a role in DNA repair and has been implicated in medulloblastoma, breast cancer, and osteosarcoma (74). There are many metabolic activities associated with LRP-1. It is also involved in tumorogenesis and tumor progression (75, 76). *STAB1* has an association with bladder cancer and acute myelogenous leukemia (77). Because *CPS1* has a reputed antioncogenic role, a damaging mutation might promote oncogenesis (78). *CD36* is upregulated in many cancer cell types (79, 80). ABCA8 inhibits proliferation and metastasis of hepatocellular carcinoma (81). *AMPD3* is highly expressed in gastrointestinal stromal tumors (82) and is upregulated in lung cancers (83). *ABCA1* mutations are associated with chronic myelogenous leukemia (84). *WWOX* has been identified as a tumor-suppressive gene. WWOX deficiency has a role in the expression of the estrogen receptor and is associated with triple-negative breast cancer (85). *WWOX* contains fragile elements that are susceptible to translocations and deletions (86). Clearly, LoF mutations in genes with oncoprotective roles would be expected to increase the risk of malignancy. However, gain of function in tumor promotor genes could also be oncogenic. *WWOX*, as a fragile gene, is likely to undergo deletions and rearrangements that make it a prominent candidate for diverse cancers. The demonstration of a significant relationship of diminished HDL levels to cancer in a population study suggests many of the functional roles of HDL in cell replication remain to be discovered. Their discovery holds promise of new venues of treatment for cancer. The prominent roles of lipid metabolism in the CNS indicate that there will be important impact on neurocognitive and neurodegenerative disorders.

In conclusion, we wished to establish a better understanding of the causes of inherited low levels of plasma HDL-C. Our aim was to provide a valuable resource for compiling a catalog of gene variants that are deemed potentially damaging or pathogenic. We detected at least one damaging mutation in an HDL candidate gene in 110 participants, a little over half of the study cohort. Clearly there must be either other genes involved or variation in promoters, enhancer, silencer elements, etc., not detectable in our study, that play a role. A limitation of this study with regard to the candidate gene approach is that because our goal was to determine the relative burden of deleterious variants in a panel of HDL candidate genes among individuals with low HDL-C and we only undertook exome sequencing of such individuals, we were not able to compare the prevalence of mutations among a cohort considered to have normal levels. It must also be noted that while the whole-exome sequencing methodology employed here has some power to detect structural variants (CNVs), it often lacks sensitivity. More advanced techniques such as Linked-Read whole-genome sequencing allow for much higher success in structural variant detection as we have recently found in other studies (38, 87). A number of the gene loci identified here have significant roles in cell biology and are also associated with cancer and neurodegenerative diseases, providing promising venues for the molecular understanding of these disorders and possible roles for HDL in their etiology.

## Data availability

All relevant data are contained within the manuscript.

## Supplemental data

This article contains supplemental data (23, 24, 26, 27).

## Conflict of interest

The authors declare that they have no conflicts of interest with the contents of this article.

## References

[1] National Cholesterol Education Program (NCEP) Expert Panel on Detection, Evaluation, and Treatment of High Blood Cholesterol in Adults (Adult Treatment Panel III) (2002). Third report of the National Cholesterol Education Program (NCEP) Expert Panel on Detection, Evaluation, and Treatment of High Blood Cholesterol in Adults (Adult Treatment Panel III) final report. Circulation.

[2] Miller N.E., Thelle D.S., Forde O.H., Mjos O.D. (1977). The Tromsø heart-study. High-density lipoprotein and coronary heart-disease: a prospective case-control study. Lancet.

[3] Rhoads G.G., Gulbrandsen C.L., Kagan A. (1976). Serum lipoproteins and coronary heart disease in a population study of Hawaii Japanese men. N. Engl. J. Med..

[4] Gordon T., Castelli W.P., Hjortland M.C., Kannel W.B., Dawber T.R. (1977). High density lipoprotein as a protective factor against coronary heart disease. The Framingham study. Am. J. Med..

[5] Gordon D.J., Probstfield J.L., Garrison R.J., Neaton J.D., Castelli W.P., Knoke J.D., Jacobs D.R., Bangdiwala S., Tyroler H.A. (1989). High-density lipoprotein cholesterol and cardiovascular disease. Four prospective American studies. Circulation.

[6] Rosenson R.S., Brewer H.B., Davidson W.S., Fayad Z.A., Fuster V., Goldstein J., Hellerstein M., Jiang X.-C., Phillips M.C., Rader D.J., Remaley A.T., Rothblat G.H., Tall A.R., Yvan-Charvet L. (2012). Cholesterol efflux and atheroprotection: advancing the concept of reverse cholesterol transport. Circulation.

[7] Rohatgi A., Khera A., Berry J.D., Givens E.G., Ayers C.R., Wedin K.E., Neeland I.J., Yuhanna I.S., Rader D.R., de Lemos J.A., Shaul P.W. (2014). HDL cholesterol efflux capacity and incident cardiovascular events. N. Engl. J. Med..

[8] Ouimet M., Barrett T.J., Fisher E.A. (2019). HDL and reverse cholesterol transport. Circ. Res..

[9] Phillips M.C. (2014). Molecular mechanisms of cellular cholesterol efflux. J. Biol. Chem..

[10] Blaho V.A., Galvani S., Engelbrecht E., Liu C., Swendeman S.L., Kono M., Proia R.L., Steinman L., Han M.H., Hla T. (2015). HDL-bound sphingosine-1-phosphate restrains lymphopoiesis and neuroinflammation. Nature.

[11] Borup A., Christensen P.M., Nielsen L.B., Christoffersen C. (2015). Apolipoprotein M in lipid metabolism and cardiometabolic diseases. Curr. Opin. Lipidol..

[12] Christensen P.M., Bosteen M.H., Hajny S., Nielsen L.B., Christoffersen C. (2017). Apolipoprotein M mediates sphingosine-1-phosphate efflux from erythrocytes. Sci. Rep..

[13] Ronsein G.E., Vaisar T. (2017). Inflammation, remodeling, and other factors affecting HDL cholesterol efflux. Curr. Opin. Lipidol..

[14] Soran H., Schofield J.D., Durrington P.N. (2015). Antioxidant properties of HDL. Front. Pharmacol..

[15] Voight B.F., Peloso G.M., Orho-Melander M., Frikke-Schmidt R., Barbalic M., Jensen M.K., Hindy G., Holm H., Ding E.L., Johnson T., Schunkert H., Samani N.J., Clarke R., Hopewell J.C., Thompson J.F. (2012). Plasma HDL cholesterol and risk of myocardial infarction: a Mendelian randomisation study. Lancet.

[16] Haase C.L., Tybjaerg-Hansen A., Ali Qayyum A., Schou J., Nordestgaard B.G., Frikke-Schmidt R. (2012). LCAT, HDL cholesterol and ischemic cardiovascular disease: a Mendelian randomization study of HDL cholesterol in 54,500 individuals. J. Clin. Endocrinol. Metab..

[17] Geller A.S., Polisecki E.Y., Diffenderfer M.R., Asztalos B.F., Karathanasis S.K., Hegele R.A., Schaefer E.J. (2018). Genetic and secondary causes of severe HDL deficiency and cardiovascular disease. J. Lipid Res..

[18] Robertson F.W., Cumming A.M., Douglas A.S., Smith E.B., Kenmure A.C. (1980). Coronary heart disease in North-East Scotland a study of genetic and environmental variation in serum lipoproteins and other variables. Scott. Med. J..

[19] Austin M.A., King M.C., Bawol R.D., Hulley S.B., Friedman G.D. (1987). Risk factors for coronary heart disease in adult female twins. Genetic heritability and shared environmental influences. Am. J. Epidemiol..

[20] Tall A.R., Breslow J.L., Rubin E.M., Valle D.L., Antonarakis S., Ballabio A., Beaudet A.L., Mitchell G.A. (2007). Metabolic & Molecular Bases of Inherited Disease.

[21] Francone O.L., Ishida B.Y., de la Llera-Moya M., Royer L., Happe C., Zhu J., Chalkey R.J., Schaefer P., Cox C., Burlingame A., Kane J.P., Rothblat G.H. (2011). Disruption of the murine procollagen C-proteinase enhancer 2 gene causes accumulation of pro-apoA-I and increased HDL levels. J. Lipid Res..

[22] Lee J.C., Weissglas-Volkov D., Kyttälä M., Dastani Z., Cantor R.M., Sobel E.M., Plaisier C.L., Engert J.C., van Greevenbroek M.M.J., Kane J.P., Malloy M.J., Pullinger C.R., Huertas-Vazquez A., Aguilar-Salinas C.A., Tusie-Luna T. (2008). WW-domain-containing oxidoreductase is associated with low plasma HDL-C levels. Am. J. Hum. Genet..

[23] Kathiresan S., Melander O., Guiducci C., Surti A., Burtt N.P., Rieder M.J., Cooper G.M., Roos C., Voight B.F., Havulinna A.S., Wahlstrand B., Hedner T., Corella D., Tai E.S., Ordovas J.M. (2008). Six new loci associated with blood low-density lipoprotein cholesterol, high-density lipoprotein cholesterol or triglycerides in humans. Nat. Genet..

[24] Lange L.A., Willer C.J., Rich S.S. (2015). Recent developments in genome and exome-wide analyses of plasma lipids. Curr. Opin. Lipidol..

[25] Willer C.J., Schmidt E.M., Sengupta S., Peloso G.M., Gustafsson S., Kanoni S., Ganna A., Chen J., Buchkovich M.L., Mora S., Beckmann J.S., Bragg-Gresham J.L., Chang H.-Y., Demirkan A.S.E., Den Hertog H.M. (2013). Discovery and refinement of loci associated with lipid levels. Nat. Genet..

[26] Teslovich T.M., Musunuru K., Smith A.V., Edmondson A.C., Stylianou I.M., Koseki M., Pirruccello J.P., Ripatti S., Chasman D.I., Willer C.J., Johansen C.T., Fouchier S.W., Isaacs A., Peloso G.M., Barbalic M. (2010). Biological, clinical and population relevance of 95 loci for blood lipids. Nature.

[27] Willer C.J., Sanna S., Jackson A.U., Scuteri A., Bonnycastle L.L., Clarke R., Heath S.C., Timpson N.J., Najjar S.S., Stringham H.M., Strait J., Duren W.L., Maschio A., Busonero F., Mulas A. (2008). Newly identified loci that influence lipid concentrations and risk of coronary artery disease. Nat. Genet..

[28] Myers L.H., Phillips N.R., Havel R.J. (1976). Mathematical evaluation of methods for estimation of the concentration of the major lipid components of human serum lipoproteins. J. Lab. Clin. Med..

[29] Pullinger C.R., Aouizerat B.E., Movsesyan I., Durlach V., Sijbrands E.J., Nakajima K., Poon A., Dallinga-Thie G.M., Hattori H., Green L.L., Kwok P.Y., Havel R.J., Frost P.H., Malloy M.J., Kane J.P. (2008). An apolipoprotein A-V gene SNP is associated with marked hypertriglyceridemia among Asian-American patients. J. Lipid Res..

[30] Shiffman D., Ellis S.G., Rowland C.M., Malloy M.J., Luke M.M., Iakoubova O.A., Pullinger C.R., Cassano J., Aouizerat B.E., Fenwick R.G., Reitz R.E., Catanese J.J., Leong D.U., Zellner C., Sninsky J.J. (2005). Identification of four gene variants associated with myocardial infarction. Am. J. Hum. Genet..

[31] Khetarpal S.A., Babb P.L., Zhao W., Hancock-Cerutti W.F., Brown C.D., Rader D.J., Voight B.F. (2018). Multiplexed targeted resequencing identifies coding and regulatory variation underlying phenotypic extremes of high-density lipoprotein cholesterol in humans. Circ. Genom. Precis. Med..

[32] (1980). Population Studies Data Book: Vol. I, The Prevalence Study.

[33] Pullinger C.R., Hennessy L.K., Chatterton J.E., Liu W.Q., Love J.A., Mendel C.M., Frost P.H., Malloy M.J., Schumaker V.N., Kane J.P. (1995). Familial ligand-defective apolipoprotein B - identification of a new mutation that decreases LDL receptor binding affinity. J. Clin. Invest..

[34] Warnick G.R., Benderson J., Albers J.J. (1982). Dextran sulfate-Mg2+ precipitation procedure for quantitation of high-density-lipoprotein cholesterol. Clin. Chem..

[35] Friedewald W.T., Levy R.I., Fredrickson D.S. (1972). Estimation of the concentration of low-density lipoprotein cholesterol in plasma, without use of the preparative ultracentrifuge. Clin. Chem..

[36] Quinn A.G., Schwemberger R., Stock E.O., Movsesyan I., Axtell A., Chang S., Ishida B.Y., Malloy M.J., Kane J.P., Pullinger C.R. (2017). Moderate statin treatment reduces prebeta-1 high-density lipoprotein levels in dyslipidemic patients. J. Clin. Lipidol..

[37] Jin S.C., Homsy J., Zaidi S., Lu Q., Morton S., DePalma S.R., Zeng X., Qi H., Chang W., Sierant M.C., Hung W.-C., Haider S., Zhang J., Knight J., Bjornson R.D. (2017). Contribution of rare inherited and de novo variants in 2,871 congenital heart disease probands. Nat. Genet..

[38] Wong K.H.Y., Levy-Sakin M., Ma W., Gonzaludo N., Mak A.C.Y., Vaka D., Poon A., Chu C., Lao R., Balamir M., Grenville Z., Wong N., Kane J.P., Kwok P.-Y., Malloy M.J. (2019). Three patients with homozygous familial hypercholesterolemia: genomic sequencing and kindred analysis. Mol. Genet. Genomic Med..

[39] Li H., Durbin R. (2009). Fast and accurate short read alignment with Burrows-Wheeler transform. Bioinformatics.

[40] Mckenna A., Hanna M., Banks E., Sivachenko A., Cibulskis K., Kernytsky A., Garimella K., Altshuler D., Gabriel S., Daly M., DePristo M.A. (2010). The genome analysis toolkit: a MapReduce framework for analyzing next-generation DNA sequencing data. Genome Res..

[41] Garrison E., Marth E. (2012). Haplotype-based variant detection from short-read sequencing. arXiv.

[42] Li H., Handsaker B., Wysoker A., Fennell T., Ruan J., Homer N., Marth G., Abecasis G., Durbin R., 1000 Genome Project Data Processing Subgroup (2009). The sequence alignment/map format and SAMtools. Bioinformatics.

[43] Lai Z., Markovets A., Ahdesmaki M., Chapman B., Hofmann O., McEwen R., Johnson J., Dougherty B., Barrett J.C., Dry J.R. (2016). VarDict: a novel and versatile variant caller for next-generation sequencing in cancer research. Nucleic Acids Res..

[44] Wang K., Li M., Hakonarson H. (2010). ANNOVAR: functional annotation of genetic variants from high-throughput sequencing data. Nucleic Acids Res..

[45] Landrum M.J., Lee J.M., Benson M., Brown G., Chao C., Chitipiralla S., Gu B., Hart J., Hoffman D., Hoover J., Jang W., Katz K., Ovetsky M., Riley G., Sethi A. (2016). ClinVar: public archive of interpretations of clinically relevant variants. Nucleic Acids Res..

[46] Lek M., Karczewski K.J., Minikel E.V., Samocha K.E., Banks E., Fennell T., O'Donnell-Luria A.H., Ware J.S., Hill A.J., Cummings B.B., Tukiainen T., Birnbaum D.P., Kosmicki J.A., Duncan L.E., Estrada K. (2016). Analysis of protein-coding genetic variation in 60,706 humans. Nature.

[47] Liu X., Jian X., Boerwinkle E. (2011). dbNSFP: a lightweight database of human nonsynonymous SNPs and their functional predictions. Hum. Mutat..

[48] Li Q., Wang K. (2017). InterVar: clinical interpretation of genetic variants by the 2015 ACMG-AMP guidelines. Am. J. Hum. Genet..

[49] Jagadeesh K.A., Wenger A.M., Berger M.J., Guturu H., Stenson P.D., Cooper D.N., Bernstein J.A., Bejerano G. (2016). M-CAP eliminates a majority of variants of uncertain significance in clinical exomes at high sensitivity. Nat. Genet..

[50] Ioannidis N.M., Rothstein J.H., Pejaver V., Middha S., McDonnell S.K., Baheti S., Musolf A., Li Q., Holzinger E., Karyadi D., Cannon-Albright L.A., Teerlink C.C., Stanford J.L., Isaacs W.B., Xu J. (2016). REVEL: an ensemble method for predicting the pathogenicity of rare missense variants. Am. J. Hum. Genet..

[51] Li H. (2014). Toward better understanding of artifacts in variant calling from high-coverage samples. Bioinformatics.

[52] Dong C., Wei P., Jian X., Gibbs R., Boerwinkle E., Wang K., Liu X. (2015). Comparison and integration of deleteriousness prediction methods for nonsynonymous SNVs in whole exome sequencing studies. Hum. Mol. Genet..

[53] Mak A.C.Y., Pullinger C.R., Tang L.F., Wong J.S., Deo R.C., Schwarz J.-M., Gugliucci A., Movsesyan I., Ishida B.Y., Chu C., Poon A., Kim P., Stock E.O., Schaefer E.J., Asztalos B.F. (2014). Effects of the absence of apolipoprotein E on lipoproteins, neurocognitive function, and retinal function. JAMA Neurol..

[54] Edmondson A.C., Braund P.S., Stylianou I.M., Khera A.V., Nelson C.P., Wolfe M.L., Derohannessian S.L., Keating B.J., Qu L., He J., Tobin M.D., Tomaszewski M., Baumert J., Klopp N., Döring A. (2011). Dense genotyping of candidate gene loci identifies variants associated with high-density lipoprotein cholesterol. Circ. Cardiovasc. Genet..

[55] Richards S., Aziz N., Bale S., Bick D., Das S., Gastier-Foster J., Grody W.W., Hegde M., Lyon E., Spector E., Voelkerding K., Rehm H.L., ACMG Laboratory Quality Assurance Committee (2015). Standards and guidelines for the interpretation of sequence variants: a joint consensus recommendation of the American College of Medical Genetics and Genomics and the Association for Molecular Pathology. Genet. Med..

[56] Samocha K.E., Robinson E.B., Sanders S.J., Stevens C., Sabo A., McGrath L.M., Kosmicki J.A., Rehnstrom K., Mallick S., Kirby A., Wall D.P., MacArthur D.G., Gabriel S.B., DePristo M., Purcell S.M. (2014). A framework for the interpretation of de novo mutation in human disease. Nat. Genet..

[57] Mi H., Muruganujan A., Ebert D., Huang X., Thomas P.D. (2019). PANTHER version 14: more genomes, a new PANTHER GO-slim and improvements in enrichment analysis tools. Nucleic Acids Res..

[58] Fromer M., Purcell S.M. (2014). Using XHMM software to detect copy number variation in whole-exome sequencing data. Curr. Protoc. Hum. Genet..

[59] Stock E.O., Ferrara C.T., O'Connor P.M., Naya-Vigne J.M., Frost P.H., Malloy M.J., Kane J.P., Pullinger C.R. (2018). Levels of prebeta-1 high-density lipoprotein are elevated in 3 phenotypes of dyslipidemia. J. Clin. Lipidol..

[60] Seino Y., Miki T., Kiyonari H., Abe T., Fujimoto W., Kimura K., Takeuchi A., Takahashi Y., Oiso Y., Iwanaga T., Seino S. (2008). Isx participates in the maintenance of vitamin A metabolism by regulation of beta-carotene 15,15'-monooxygenase (Bcmo1) expression. J. Biol. Chem..

[61] Gregg R.E., Wetterau J.R. (1994). The molecular basis of abetalipoproteinemia. Curr. Opin. Lipidol..

[62] Brown M.S., Hobbs H.H., Goldstein J.L., Valle D.L., Antonarakis S., Ballabio A., Beaudet A.L., Mitchell G.A. (2007). Metabolic & Molecular Bases of Inherited Disease.

[63] Guerin M. (2012). Reverse cholesterol transport in familial hypercholesterolemia. Curr. Opin. Lipidol..

[64] Escola-Gil J.C., Rotllan N., Julve J., Blanco-Vaca F. (2021). Reverse cholesterol transport dysfunction is a feature of familial hypercholesterolemia. Curr. Atheroscler. Rep..

[65] Kzhyshkowska J., Gratchev A., Goerdt S. (2006). Stabilin-1, a homeostatic scavenger receptor with multiple functions. J. Cell. Mol. Med..

[66] Motazacker M.M., Pirhonen J., van Capelleveen J.C., Weber-Boyvat M., Kuivenhoven J.-A., Shah S., Hovingh G.K., Metso J., Li S., Ikonen E., Jauhiainen M., Dallinga-Thie G.M., Olkkonen V.M. (2016). A loss-of-function variant in OSBPL1A predisposes to low plasma HDL cholesterol levels and impaired cholesterol efflux capacity. Atherosclerosis.

[67] Au D.T., Strickland D.K., Muratoglu S.C. (2017). The LDL receptor-related protein 1: at the crossroads of lipoprotein metabolism and insulin signaling. J. Diabetes Res..

[68] Sasaki K., Tachikawa M., Uchida Y., Hirano S., Kadowaki F., Watanabe M., Ohtsuki S., Terasaki T. (2018). ATP-binding cassette transporter A subfamily 8 is a sinusoidal efflux transporter for cholesterol and taurocholate in mouse and human liver. Mol. Pharm..

[69] Trigueros-Motos L., van Capelleveen J.C., Torta F., Castaño D., Zhang L.-H., Chai E.C., Kang M., Dimova L.G., Schimmel A.W.M., Tietjen I., Radomski C., Tan L.J., Thiam C.H., Narayanaswamy P., Wu D.H. (2017). ABCA8 regulates cholesterol efflux and high-density lipoprotein cholesterol levels. Arterioscler. Thromb. Vasc. Biol..

[70] Hirayasu K., Ohashi J., Kashiwase K., Takanashi M., Satake M., Tokunaga K., Yabe T. (2006). Long-term persistence of both functional and non-functional alleles at the leukocyte immunoglobulin-like receptor A3 (LILRA3) locus suggests balancing selection. Hum. Genet..

[71] Schaefer E.J., Brousseau M.E., Diffenderfer M.R., Cohn J.S., Welty F.K., O'Connor J., Dolnikowski G.G., Wang J., Hegele R.A., Jones P.J. (2001). Cholesterol and apolipoprotein B metabolism in Tangier disease. Atherosclerosis.

[72] Pedersen K.M., Colak Y., Bojesen S.E., Nordestgaard B.G. (2020). Low high-density lipoprotein and increased risk of several cancers: 2 population-based cohort studies including 116,728 individuals. J. Hematol. Oncol..

[73] Aldaz C.M., Hussain T. (2020). WWOX loss of function in neurodevelopmental and neurodegenerative disorders. Int. J. Mol. Sci..

[74] Reiss K., Del Valle L., Lassak A., Trojanek J. (2012). Nuclear IRS-1 and cancer. J. Cell. Physiol..

[75] Langlois B., Perrot G., Schneider C., Henriet P., Emonard H., Martiny L., Dedieu S. (2010). LRP-1 promotes cancer cell invasion by supporting ERK and inhibiting JNK signaling pathways. PLoS One.

[76] Xing P., Liao Z., Ren Z., Zhao J., Song F., Wang G., Chen K., Yang J. (2016). Roles of low-density lipoprotein receptor-related protein 1 in tumors. Chin. J. Cancer.

[77] Hollmen M., Figueiredo C.R., Jalkanen S. (2020). New tools to prevent cancer growth and spread: a 'Clever' approach. Br. J. Cancer.

[78] Cancer Genome Atlas Research Network (2017). Comprehensive and integrative genomic characterization of hepatocellular carcinoma. Cell.

[79] Ladanyi A., Mukherjee A., Kenny H.A., Johnson A., Mitra A.K., Sundaresan S., Nieman K.M., Pascual G., Benitah S.A., Montag A., Yamada S.D., Abumrad N.A., Lengyel E. (2018). Adipocyte-induced CD36 expression drives ovarian cancer progression and metastasis. Oncogene.

[80] Wang J., Li Y. (2019). CD36 tango in cancer: signaling pathways and functions. Theranostics.

[81] Cui Y., Liang S., Zhang S., Zhang C., Zhao Y., Wu D., Wang J., Song R., Wang J., Yin D., Liu Y., Pan S., Liu X., Wang Y., Han J. (2020). ABCA8 is regulated by miR-374b-5p and inhibits proliferation and metastasis of hepatocellular carcinoma through the ERK/ZEB1 pathway. J. Exp. Clin. Cancer Res..

[82] Wong M., Funasaka K., Obayashi T., Miyahara R., Hirooka Y., Hamaguchi M., Goto H., Senga T. (2017). AMPD3 is associated with the malignant characteristics of gastrointestinal stromal tumors. Oncol. Lett..

[83] Fernandez P., Carretero J., Medina P.P., Jimenez A.I., Rodriguez-Perales S., Paz M.F., Cigudosa J.C., Esteller M., Lombardia L., Morente M., Sanchez-Verde L., Sotelo T., Sanchez-Cespedes M. (2004). Distinctive gene expression of human lung adenocarcinomas carrying LKB1 mutations. Oncogene.

[84] Viaud M., Abdel-Wahab O., Gall J., Ivanov S., Guinamard R., Sore S., Merlin J., Ayrault M., Guilbaud E., Jacquel A., Auberger P., Wang N., Levine R.L., Tall A.R., Yvan-Charvet L. (2020). ABCA1 exerts tumor-suppressor function in myeloproliferative neoplasms. Cell Rep..

[85] Abdeen S.K., Aqeilan R.I. (2019). Decoding the link between WWOX and p53 in aggressive breast cancer. Cell Cycle.

[86] Iliopoulos D., Guler G., Han S.Y., Druck T., Ottey M., McCorkell K.A., Huebner K. (2006). Roles of FHIT and WWOX fragile genes in cancer. Cancer Lett..

[87] Wong K.H.Y., Levy-Sakin M., Kwok P.Y. (2018). De novo human genome assemblies reveal spectrum of alternative haplotypes in diverse populations. Nat. Commun..

