# Supplementary material for: Whole-exome sequencing reveals damaging gene variants associated with hypoalphalipoproteinemia
Source: J Lipid Res. 2022 Apr 20;63(6):100209. doi: 10.1016/j.jlr.2022.100209 (PMC9126845; doi:10.1016/j.jlr.2022.100209)
Supplement: Supplemental Figures S1–S3 and Tables S1–S6 [file mmc1.pdf]

Supplemental Figure S1

Distribution of 29 Damaging Rare Variants in 15 of 67 LDL Candidate Genes

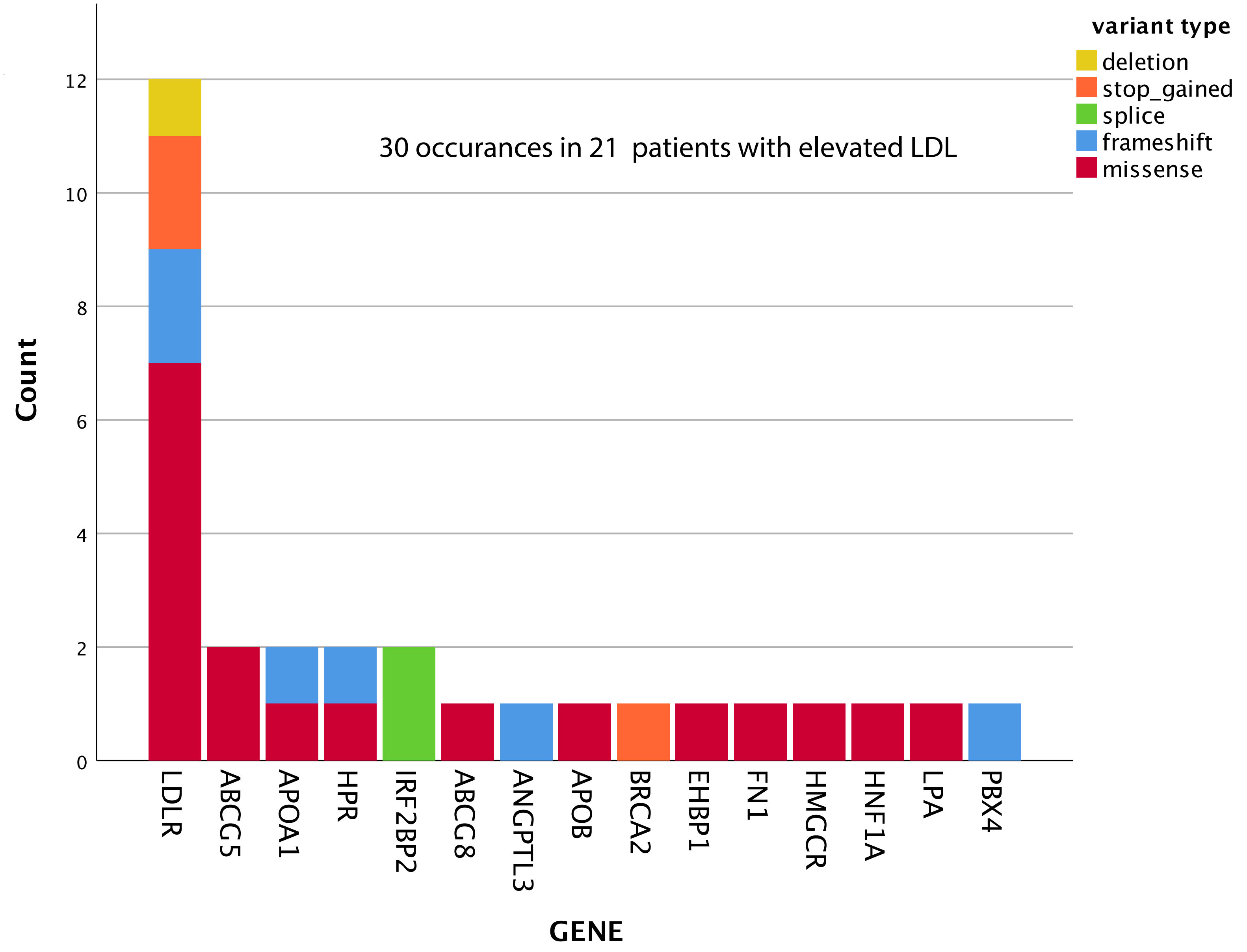

Supplemental Figure S2

Damaging Variants in TG Candidate Genes among patients with elevated TG

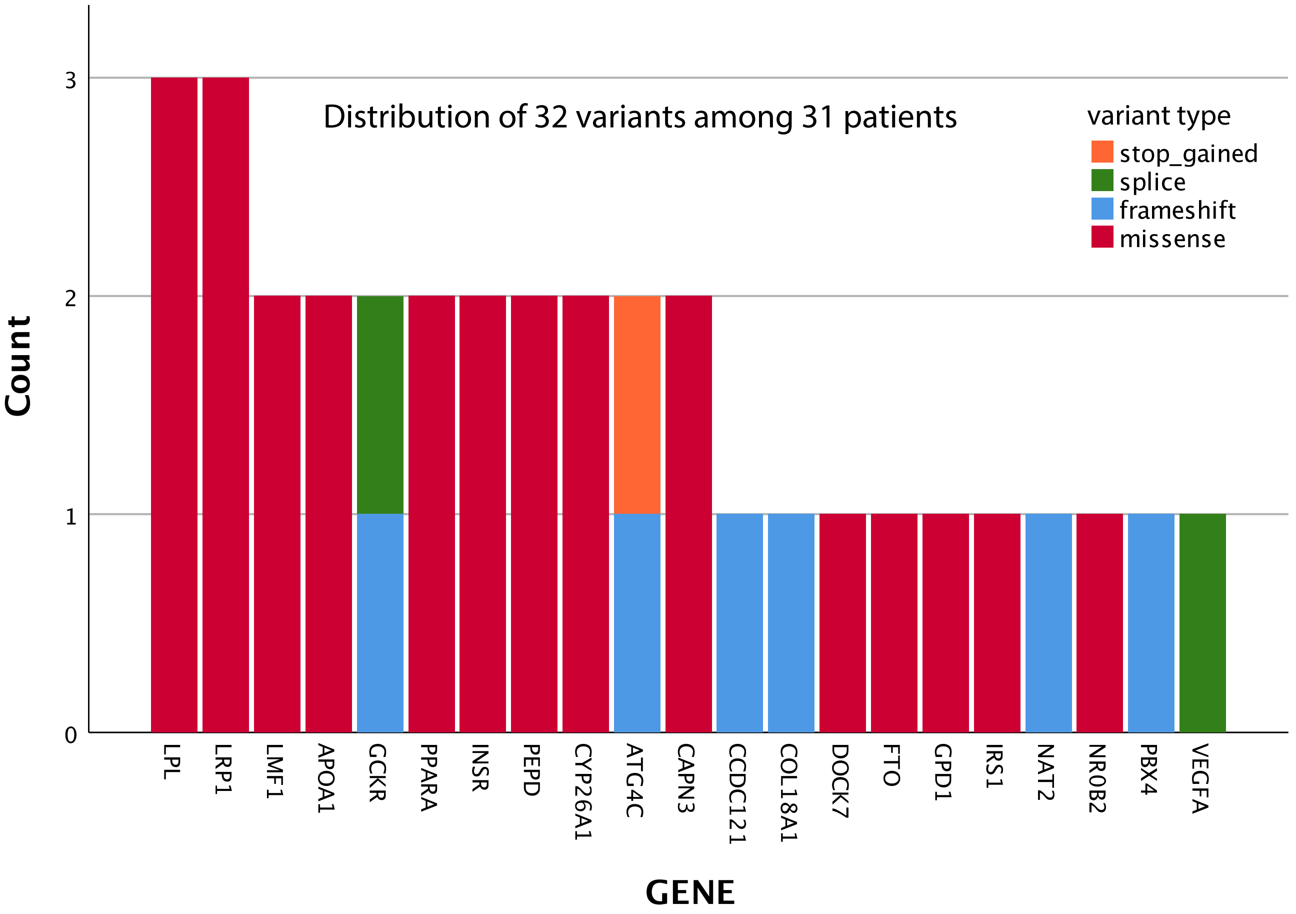

Supplemental Figure S3

Box plot showing the distribution of levels of HDL cholesterol among 128 individuals from 18 families with Familial Hypercholesterolemia due to deleterious *LDLR* gene mutations.

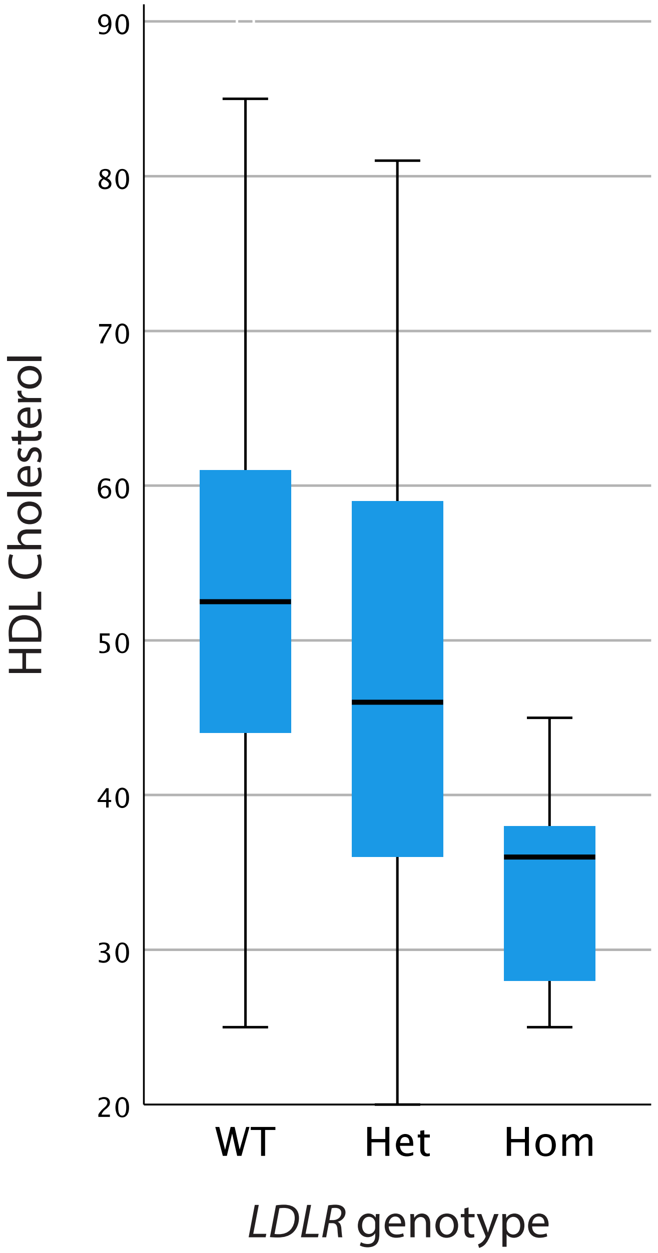

Supplemental Table S1. A list of 594 lipid metabolism-related candidate genes/loci.

Gene	NCBI ID	Chr	Start	Stop	Kathiresan 2008	Willer 2008	Teslovich 2010	Small 2011	Brouwers 2012	Lange 2015	NCBI*	Other
A2M	2	12	9220304	9268558							+	
A2ML1	144568	12	8822472	8887202						+		
ABCA1	19	9	107543283	107690436	+	+					+	
ABCA7	10347	19	1040102	1065571				+				
ABCA8	10351	17	66863430	66951533			+					
ABCB1	5243	7	87132948	87342564							+	
ABCB11	8647	2	169779449	169887833							+	
ABCB4	5244	7	87031361	87105019							+	
ABCC1	4363	16	16043434	16236931							+	
ABCC2	1244	10	101542463	101611662							+	
ABCC3	8714	17	48712218	48769063							+	
ABCD1	215	X	152990323	153010216							+	
ABCD2	225	12	39945022	40013843							+	
ABCD3	5825	1	94883933	94984219							+	
ABCG1	9619	21	43619799	43717354							+	
ABCG5	64240	2	44039611	44065958							+	
ABCG8	64241	2	44066103	44105605							+	
ABHD5	51099	3	43732375	43764217							+	
ABO	28	9	136130563	136150630								+
ACAA1	30	3	38164201	38178733							+	
ACAA2	10449	18	47309874	47340251	+							
ACACA	31	17	35441927	35766902							+	
ACACB	32	12	109577202	109706031							+	
ACAD11	84129	3	132558138	132660131,						+		
ACADL	33	2	211052714	211090215							+	
ACADM	34	1	76190043	76229355							+	
ACADS	35	12	121163571	121177811							+	
ACADVL	37	17	7123153	7128585							+	

ACAT1	38	11	107992258	108018895				+
ACAT2	39	6	160182989	160200087				+
ACER1	125981	19	6306510	6333640				+
ACER2	340485	9	19408925	19452500				+
ACER3	55331	11	76571917	76734850				+
ACLY	47	17	40023179	40075272				+
ACOT8	10005	20	44470361	44486035				+
ACOX1	51	17	73937588	73975515				+
ACOX2	8309	3	58490863	58522929				+
ACOX3	8310	4	8368009	8442452				+
ACSL1	2180	4	185676749	185747215				+
ACSL3	2181	2	223725732	223808119				+
ACSL4	2182	X	108884564	108976621				+
ACSL5	51703	10	114133916	114188138				+
ACSL6	23305	5	131289152	131347349				+
ADAM28	10863	8	24151580	24212726	+			
ADD1	118	4	2843727	2930076		+		
ADH5	128	4	99070978	99088788			+	
ADIPOR2	79602	12	1800247	1897845	+			
AGPAT1	10554	6	32135983	32145888				+
AGPAT2	10555	9	139567595	139581911				+
AGPAT3	56894	21	45285116	45407475				+
AGPAT4	56895	6	161551057	161695107				+
AGPAT5	55326	8	6565878	6619024				+
AGPAT6	137964	8	41435707	41482520				+
AGPAT9	84803	4	84457653	84527026				+
AGPS	8540	2	178257471	178408564				+
AGT	183	1	230838269	230850336				+
AKR1B1	231	7	134127107	134143888				+
AKR1C4	1109	10	5238798	5260912				+
AKR1D1	6718	7	137761178	137803050				+
AKT1	207	14	104769349	104795743			+	

ALB	213	4	74269972	74287129							+	
ALOX5	240	10	45869629	45941565							+	
AMACR	23600	5	33987091	34008220							+	
AMPD3	272	11	10471868	10529126						+		
ANGPTL1	9068	1	178849535	178871353						+		
ANGPTL3	27329	1	63063187	63071180	+	+						
ANGPTL4	51129	19	8429011	8439257							+	
ANGPTL8	55908	19	11239619	11241943			+			+		
ANKRD1	27063	10	92671857	92681032							+	
ANXA9	8416	1	150982023	150995638						+		
APH1B	83464	15	63569749	63601325				+				
APOA1	335	11	116706469	116708338	+	+					+	
APOA2	336	1	161192083	161193418							+	
APOA4	337	11	116691418	116694011		+					+	
APOA5	116519	11	116660086	116663136		+					+	
APOB	338	2	21224301	21266945	+	+					+	
APOBEC1	339	12	7649400	7670599					+			
APOBR	55911	16	28505993	28510282								+
APOC1	341	19	45417921	45422606		+					+	
APOC2	344	19	45449243	45452818		+					+	
APOC3	345	11	116700624	116703787		+					+	
APOC4	346	19	45445495	45448751		+						
APOD	347	3	195295573	195311076								+
APOE	348	19	45409039	45412650		+					+	
APOF	319	12	56754355	56756583				+				
APOH	350	17	64208147	64225556								+
APOM	55937	6	31623671	31625987				+				
ARL15	54622	5	53180614	53606403			+			+		
ARSD	414	X	2822011	2847392				+				
ASAP3	55616	1	23428563	23484631						+		
ATF6	22926	1	161766244	161964070					+			
ATG4C	84938	1	63249803	63330050	+							

ATG5	9474	6	106632352	106773695						+	
ATG7	10533	3	11272324	11564704						+	
B3GALT4	8705	6	33244917	33246602		+					
BAAT	570	9	104122699	104147287							+
BCL7B	9275	7	72950686	72972024	+						
BCMO1	53630	16	81272296	81324747							+
BDH1	622	3	197236654	197300194							+
BMP1	649	8	22022653	22069839							+
BRAP	8315	12	112079950	112123790			+				
BRCA2	675	13	32315480	32399672						+	
BUD13	84811	11	116618886	116643714	+						
C5AR2	27202	19	47332147	47342015					+		
C6orf106	64771	6	34555065	34664625			+				
C8orf82	414919	8	145751603	145754458				+			
CACNG5	27091	17	64873451	64881357				+			
CAPN3	825	15	42651698	42704515			+				
CARM1	10498	19	10982253	11033448							+
CAV1	857	7	116164839	116201239							+
CAV2	858	7	116139444	116148595				+			
CD36	948	7	80231504	80308593							+
CD93	22918	20	23059993	23066977				+			
CEL	1056	9	135937365	135947248							+
CELSR2	1952	1	109792641	109818378	+	+					
CERS1	10715	19	18979361	19006953							+
CERS2	29956	1	150965173	150975003						+	
CERS3	204219	15	100940600	101084925							+
CERS4	79603	19	8274217	8327305					+		
CERS5	91012	12	50523581	50561097							+
CERS6	253782	2	169312835	169631152							+
CETP	1071	16	56995835	57017756	+	+					+
CH25H	9023	10	90965694	90967071							+
CHD9	80205	16	53088945	53361414							+

CILP2	148113	19	19649074	19657468	+	+					
CITED2	10370	6	139693392	139695787			+		+		
CLPS	1208	6	35762760	35765102						+	
CLU	1191	8	27454434	27472328							+
CMIP	80790	16	81478775	81745367			+				
CMTM5	116173	14	23376439	23379772					+		
COBLL1	22837	2	165541256	165697928			+		+		
COL18A1	80781	21	45405137	45513720					+		
COL4A3BP	10087	5	74666928	74807806						+	
CPS1	1373	2	210477682	210679107					+		
CPT1A	1374	11	68522088	68609399						+	
CPT1B	1375	22	51007290	51017096						+	
CPT2	1376	1	53662101	53679869						+	
CRABP2	1382	1	156699606	156713174				+			
CRAT	1384	9	131857073	131873070						+	
CREB3L3	84699	19	4153598	4173051							
CREBBP	1387	16	3775055	3930121						+	
CROT	54677	7	86974951	87029112						+	
CSNK1G2	1455	19	1941161	1981337						+	
CSNK1G3	1456	5	123512099	123617045					+		
CTF1	1489	16	30907928	30914881			+				
CTGF	1490	6	132269316	132272518						+	
CUBN	8029	10	16865965	17171816						+	
CYP11A1	1583	15	74630103	74660081						+	
CYP11B1	1584	8	143953773	143961236						+	
CYP11B2	1585	8	143991975	143999259						+	
CYP17A1	1586	10	104590288	104597290						+	
CYP19A1	1588	15	51500254	51630795						+	
CYP1A1	1543	15	75011883	75017877						+	
CYP1A2	1544	15	75041184	75048941						+	
CYP21A2	1589	6	32006082	32009419						+	
CYP24A1	1591	20	52769988	52790516						+	

CYP26A1	1592	10	94833232	94837641				+
CYP27A1	1593	2	219646472	219680016				+
CYP27B1	1594	12	58156117	58160976				+
CYP2B6	1555	19	41497204	41524301				+
CYP2C9	1559	10	96698415	96749148				+
CYP2E1	1571	10	135340867	135352620				+
CYP2R1	120227	11	14899555	14913751				+
CYP39A1	51302	6	46517445	46620523				+
CYP3A4	1576	7	99354604	99381808				+
CYP46A1	10858	14	100150755	100193638				+
CYP4A11	1579	1	47394846	47407156				+
CYP4B1	1580	1	47264670	47285021				+
CYP51A1	1595	7	91741463	91764059				+
CYP7A1	1581	8	59402737	59412720				+
CYP7B1	9420	8	65508529	65711348				+
CYP8B1	1582	3	42913684	42917633				+
DAGLB	221955	7	6409116	6448012			+	
ECI1	1632	16	2289873	2301602				+
DECR1	1666	8	91013580	91064227				+
DEGS1	8560	1	224370928	224381143				+
DEGS2	123099	14	100612753	100626012				+
DGAT1	8694	8	145538247	145550567				+
DGAT2	84649	11	75479778	75512579				+
DHCR24	1718	1	55315300	55352921				+
DHCR7	1717	11	71145457	71159477				+
DLG4	1742	17	7189890	7220050			+	
DNAH11	8701	7	21582833	21941457		+		
DOCK7	85440	1	62920397	63153969	+			
DPEP2	64174	16	68021293	68033364				+
GLB1	2720	3	33038100	33138694				+
ECHS1	1892	10	135175987	135186908				+
EHBP1	23301	2	62673851	63046487			+	

ELOVL1	64834	1	43829072	43833699					+	
ELOVL2	54898	6	10980992	11044624					+	
ELOVL3	83401	10	103986143	103989346					+	
ELOVL4	6785	6	80624529	80657315					+	
ELOVL5	60481	6	53132196	53213942					+	
ELOVL6	79071	4	110970229	111119820					+	
ELOVL7	79993	5	60047616	60140101					+	
ERGIC3	51614	20	34129778	34145405	+					
EVI5	7813	1	92974253	93257961	+					
FABP1	2168	2	88422510	88427578					+	
FABP2	2169	4	120238405	120243316					+	
FABP4	2167	8	82390732	82395473					+	
FABP6	2172	5	159614374	159665729					+	
FADS1	3992	11	61567097	61584529					+	
FADS2	9415	11	61595713	61634825	+					
FADS3	3995	11	61640998	61659006	+					
FAM117B	150864	2	202635178	202769757				+		
FAM13A	10144	4	88725954	89111398				+		
FAR1	84188	11	13690206	13753893					+	
FAR2	55711	12	29376598	29487006					+	
FASN	2194	17	80036214	80056106					+	
FDFT1	2222	8	11660190	11696818					+	
FDPS	2224	1	155278539	155290457					+	
FFAR4	338557	10	93566204	93590072						+
FGF21	26291	19	49259344	49261582		+				
FHL2	2274	2	105977283	106055230					+	
FN1	2335	2	215360440	215436167				+		
FOXC2	2303	16	86567251	86568933			+			
FRK	2444	6	116262693	116381921	+					
FRMD5	84978	15	44165730	44487429	+					
FTO	79068	16	53703963	54114467				+		
G0S2	50486	1	209675325	209676390						+

GAL	51083	11	68684515	68691175					+		
GALNT2	2590	1	230202956	230417875	+	+					
GC	2638	4	72607410	72649888							+
GCKR	2646	2	27719706	27746551	+	+					
GGPS1	9453	1	235491753	235507847							+
GGT5	2687	22	24615622	24641110							+
GK	2710	X	30671476	30748725							+
GLIPR1	11010	12	75874513	75895716							+
GNB1	2782	1	1716729	1822495				+			
GNPAT	8443	1	231376919	231413719							+
GOT2	2806	16	58741035	58768246							+
GPAM	57678	10	113909622	113943525							+
GPAT2	150763	2	96687694	96700727							+
GPD1	2819	12	50497801	50505096							+
GPD2	2820	2	157291965	157442915							+
GPIHBP1	338328	8	144295068	144299044				+			
HCAR2	338442	12	123185840	123187904				+			
HCAR3	8843	12	123199303	123201439				+			
GPR146	115330	7	1044573	1059269						+	
GRHL1	29841	2	10091792	10142412							+
GRIN3A	116443	9	104331634	104500862		+					
GSK3B	2932	3	119821321	120094417						+	
HACL1	26061	3	15602239	15643130							+
HADH	3033	4	108910870	108956331							+
HADHA	3030	2	26413504	26467594							+
HADHB	3032	2	26467616	26513333							+
HAS1	3036	19	51713112	51723992						+	
HBS1L	10767	6	134960378	135054898						+	
HDAC3	8841	5	141000443	141016423							+
HDGF	3068	1	156742107	156752448						+	
HDLBP	3069	2	242166679	242255254				+			
HFE	3077	6	26087509	26095469			+				

HMGCL	3155	1	24128367	24151949					+	
HMGCR	3156	5	74632993	74657926	+				+	
HMGCS1	3157	5	43289493	43313595					+	
HMGCS2	3158	1	120290619	120311555					+	
HNF1A	6927	12	121416549	121440315		+				
HNF4A	3172	20	42984441	43061485		+				
HPR	3250	16	72097125	72111145		+				
HSD11B1	3290	1	209859550	209908295					+	
HSD17B1	3292	17	40703984	40707232					+	
HSD17B12	51144	11	43702143	43878169					+	
HSD17B3	3293	9	98997589	99064434					+	
HSD17B4	3295	5	118788148	118878027					+	
HSD17B7	51478	1	162760496	162782608					+	
HSD3B1	3283	1	120049826	120057681					+	
HSD3B2	3284	1	119957554	119965662					+	
HSD3B7	80270	16	30996519	31000473					+	
HSPG2	3339	1	22148737	22222804					+	
IDH1	3417	2	209100953	209119806					+	
IDI1	3422	10	1085963	1095061					+	
IDI2	91734	10	1064847	1071799					+	
IKZF1	10320	7	50303453	50405101				+		
INSIG1	3638	7	155089486	155101945						+
INSIG2	51141	2	118846050	118867597						+
INSR	3643	19	7112255	7294405,				+		
IRF2BP2	359948	1	234740015	234745271		+				
IRS1	3667	2	227596033	227663506		+				
JMJD1C	221037	10	64926985	65225722		+				
JMJD7-PLA2G4B	8681	15	42120283	42140346					+	
KAT5	10524	11	65711996	65719606				+		
KCNK17	89822	6	39299001	39314461				+		
KDSR	2531	18	60994971	61034506					+	
KLF13	51621	15	31619083	31670102			+			

KLF14	136259	7	130417396	130418888				+				
KLHL12	59349	1	202860230	202896371								+
KLHL8	57563	4	88082214	88141674			+					
LACTB	114294	15	63413999	63434260			+					
LBR	3930	1	225589204	225616519							+	
LCAT	3931	16	67973787	67978015		+					+	
LDLR	3949	19	11200057	11244506	+	+					+	
LDLRAP1	26119	1	25870076	25895377							+	
LEPR	3953	1	65420652	65637493					+			
LGMN	5641	14	93170152	93215047							+	
LILRA3	11026	19	54799854	54804265			+					
LINC01101	84931	2	120464335	120466349						+		
LIPA	3988	10	90973326	91011660								+
LIPC	3990	15	58724175	58861073	+	+					+	
LIPE	3991	19	42905666	42931578							+	
LIPF	8513	10	90424094	90438572							+	
LIPG	9388	18	47088427	47119278	+	+						
LMF1	64788	16	903634	1031318								+
LMF2	91289	22	50941376	50946135								+
LPA	4018	6	160952515	161087407							+	
LPCAT3	10162	12	7085347	7125842								+
LPIN1	23175	2	11886740	11967535							+	
LPIN2	9663	18	2916992	3011945							+	
LPIN3	64900	20	39969560	39989222							+	
LPL	4023	8	19796582	19824770	+	+					+	
LRAT	9227	4	155665163	155674270							+	
LRP1	4035	12	57522282	57607142							+	
LRP2	4036	2	169983619	170219122							+	
LRP4	4038	11	46878268	46940173			+			+		
LRP8	7804	1	53711212	53793821								+
LSS	4047	21	47608360	47648738							+	
LTA4H	4048	12	96394611	96429365							+	

LTC4S	4056	5	179220986	179223513					+	
MAFB	9935	20	39314515	39317876			+			
MAMSTR	284358	19	49216255	49222976			+			
MAP3K1	4214	5	56110900	56191979			+			
MAPKAPK2	9261	1	206858289	206907626					+	
MARC1	64757	1	220960039	220987741			+			
MARCH8	220972	10	45454585	45594907				+		
MBTPS1	8720	16	84087368	84150517						+
MBTPS2	51360	X	21857656	21903541						+
MC4R	4160	18	58038564	58040001						+
MCEE	84693	2	71336806	71357394					+	
ME1	4199	6	83920108	84140938					+	
MED1	5469	17	37560538	37607527					+	
MET	4233	7	116672359	116798386				+		
MGLL	11343	3	127407909	127542051					+	
MIR122	406906	18	56118306	56118390						+
MIR148A	406940	7	25949919	25949986				+		
MIR33A	407039	22	42296948	42297016						+
MIR370	442915	14	81431308	81431382						+
MLXIPL	51085	7	73007524	73038870	+	+				
MMAB	326625	12	109991520	110011358		+				
MOGAT2	80168	11	75428934	75442331					+	
MOGAT3	346606	7	100839010	100844302					+	
MPP3	4356	17	43800799	43833192				+		
MSL2	55167	3	135867760	135914688			+			
MSR1	4481	8	15965387	16050300						+
MTMR3	8897	22	29883165	30030868				+		
MTOR	2475	1	11166588	11322608			+			
MTTP	4547	4	100485240	100545154					+	
MUT	4594	6	49398073	49431041					+	
MVD	4597	16	88718348	88729495					+	
MVK	4598	12	110011500	110035071		+			+	

MYBPHL	343263	1	109834987	109849663	+							
MYL5	4636	4	671711	675817				+				
MYLIP	29116	6	16129317	16148479								
NAT2	10	8	18248755	18258723			+					
NCAN	1463	19	19322782	19363061		+						
NCOA1	8648	2	24807346	24993571							+	
NCOA2	10499	8	71024267	71316020							+	
NCOA3	8202	20	46130601	46285621							+	
NCOA6	23054	20	33302578	33413433							+	
NCOR1	9611	17	15933408	16118874							+	
NCOR2	9612	12	124808961	125052010							+	
NDST1	3340	5	150485823	150558211								+
NINJ2	4815	12	673462	772755				+				
NPC1	4864	18	21111463	21166581								+
NPC1L1	29881	7	44552134	44580914							+	
NR0B2	8431	1	26911484	26914076						+		
NR1H3	10062	11	47270449	47290401							+	
NR1H4	9971	12	100867679	100957643							+	
NR1I2	8856	3	119499331	119537332							+	
NR1I3	9970	1	161199456	161208000							+	
NR2F1	7025	5	92919043	92929788								+
NR2F2	7026	15	96869157	96883492								+
NR5A2	2494	1	200027602	200177424								+
NSDHL	50814	X	151999511	152037907							+	
NYNRIN	57523	14	24867992	24888494			+					
OLR1	4973	12	10310899	10324790								+
OR4C46	119749	11	54603069	54603998						+		
OSBP	5007	11	59341871	59383617							+	
OSBPL10	114884	3	31660825	31981850					+			
OSBPL1A	114876	18	21742009	21977790								+
OSBPL3	26031	7	24796537	24980212								+
OSBPL6	114880	2	179059208	179264160								+

OSBPL7	114881	17	45884733	45899147			+				
OXCT1	5019	5	41730167	41870791						+	
P4HB	5034	17	79801034	79818544						+	
PABPC4	8761	1	40026485	40042521			+		+		
PAFAH1B2	5049	11	117144284	117178173					+		
PBX4	80714	19	19672522	19729439	+						
PCCA	5095	13	100741269	101182691						+	
PCCB	5096	3	135969167	136049013						+	
PCDH15	65217	10	53802771	54801291				+			
PCOLCE2	26577	3	142536702	142608045							+
PCSK7	9159	11	117075788	117102811							+
PCSK9	255738	1	55505149	55530526	+	+					
PCTP	58488	17	53828356	53854748							+
PCYT1A	5130	3	195965253	196014584							+
PCYT1B	9468	X	24576204	24690979							+
PDE3A	5139	12	20522179	20837041			+				
PDIA2	64714	16	333118	337209						+	
PDXDC1	23042	16	14974591	15139410					+		
PDZK1	5174	1	145727666	145764207							+
PEMT	10400	17	17408877	17494994							+
PEPD	5184	19	33386949	33521893					+		
PEX11A	8800	15	90226284	90233958						+	
PGLYRP2	114770	19	15579456	15590315							+
PGS1	9489	17	76374735	76420640			+				
PHC1	1911	12	8914664	8941467					+		
PHLDB1	23187	11	118606296	118658038					+		
PHYH	5264	10	13319796	13342130						+	
PIGC	5279	1	172441457	172444090					+		
PINX1	54984	8	10622884	10697299			+				
PKD1L3	342372	16	71929538	71999978							+
PLA2G10	8399	16	14766405	14788526						+	
PLA2G12A	81579	4	110631145	110651242						+	

PLA2G12B	84647	10	74694938	74714510				+	
PLA2G1B	5319	12	120759914	120765592				+	
PLA2G2A	5320	1	20301924	20306932				+	
PLA2G2C	391013	1	20490484	20501687				+	
PLA2G2D	26279	1	20438432	20446008				+	
PLA2G2E	30814	1	20246800	20250110				+	
PLA2G2F	64600	1	20465823	20476879				+	
PLA2G3	50487	22	31530793	31536469				+	
PLA2G4A	5321	1	186798032	186958113				+	
PLA2G4B	100137049	15	42131011	42140346				+	
PLA2G4E	123745	15	42275952	42302445				+	
PLA2G5	5322	1	20396701	20418394				+	
PLA2G6	8398	22	38507502	38577761				+	
PLB1	151056	2	28718938	28866654				+	
PLD2	5338	17	4807101	4823432			+		
PLEC	5339	8	144989321	145050913	+				
PLIN1	5346	15	90207598	90222648				+	
PLIN2	123	9	19115759	19127573				+	
PLTP	5360	20	44527397	44540786				+	
PMVK	10654	1	154897208	154909484				+	
PNLIP	5406	10	118305428	118327367				+	
PNLIPRP1	5407	10	118350490	118368686				+	
PNLIPRP2	5408	10	118380465	118404654				+	
PNPLA2	57104	11	818901	825573					+
PNPLA3	80339	22	43923739	43947568					+
PNPLA5	150379	22	44275558	44287893					+
PON1	5444	7	95298357	95324572		+			
PON3	5446	7	94989181	95025702		+			
PPAP2A	8611	5	54720682	54830873				+	
PPAP2B	8613	1	56960433	57045257				+	
PPAP2C	8612	19	281043	291435				+	
PPARA	5465	22	46546499	46639653				+	

PPARD	5467	6	35310335	35395968						+	
PPARG	5468	3	12329349	12475855						+	
PPARGC1A	10891	4	23793644	23891700							+
PPARGC1B	133522	5	149109815	149234585							+
PPM1L	151742	3	160473996	160788817						+	
PPP1CA	5499	11	67165652	67169376						+	
PPP1CB	5500	2	28974614	29025806						+	
PPP1CC	5501	12	111157615	111180757						+	
PPP1R3B	79660	8	8993764	9009152			+				
PRIC285	85441	20	62189439	62205592						+	
PRKAA2	5563	1	57110990	57181008						+	
PRKAB2	5565	1	146626685	146644129						+	
PRKACA	5566	19	14202500	14228559						+	
PRKACB	5567	1	84543745	84704181						+	
PRKACG	5568	9	71627445	71629039						+	
PRKAG2	51422	7	151253200	151574316						+	
PRKCA	5578	17	66302640	66810744					+		
PRKD1	5587	14	30045685	30396899						+	
PRMT2	3275	21	48055527	48084863				+			
PSRC1	84722	1	109822176	109825790	+	+					
PTGES3	10728	12	57057125	57082078						+	
PTGIS	5740	20	48120411	48184707						+	
PTGS1	5742	9	125133229	125157981						+	
PTGS2	5743	1	186640944	186649559						+	
PXN	5829	12	120210439	120265771					+		
RAB3GAP1	22930	2	135809835	135928280			+				
RAF1	5894	3	12625100	12705700			+				
RARA	5914	17	38465423	38513895						+	
RARB	5915	3	25469754	25639423						+	
RARG	5916	12	53604353	53626036						+	
RBKS	64080	2	28004266	28113223		+					
RBM5	10181	3	50088908	50118964					+		

RBP2	5948	3	139171726	139195352			+	
RDH11	51109	14	68143517	68162510			+	
RGL1	23179	1	183605208	183897666			+	
RHOA	387	3	49396578	49449526				+
RORA	6095	15	60780483	61521502				+
RORB	6096	9	77112252	77302117				+
RORC	6097	1	151778547	151804348				+
RSPO3	84870	6	127118671	127199481		+		
RXRA	6256	9	137218316	137332431			+	
RXRG	6258	1	165370159	165414592				+
SAR1B	51128	5	133936839	133968533			+	
SBNO1	55206	12	123773656	123834988	+			
MSMO1	6307	4	166248818	166264225			+	
SC5D	6309	11	121163388	121184119			+	
SCAP	22937	3	47455184	47517445				+
SCARB1	949	12	125262174	125348519			+	
SCP2	6342	1	53392901	53517289			+	
SDC1	6382	2	20400558	20425194			+	
SETD2	29072	3	47016408	47164109		+		
SETDB2	83852	13	49444293	49495003				+
SGMS1	259230	10	52065345	52383737			+	
SGMS2	166929	4	108745721	108836203			+	
SGPL1	8879	10	72575704	72640946			+	
SGPP1	81537	14	64150934	64194756			+	
SGPP2	130367	2	223289322	223423617			+	
SIN3A	25942	15	75661720	75748124			+	
SIN3B	23309	19	16940218	16991164			+	
SLC10A1	6554	14	70242552	70264006			+	
SLC10A2	6555	13	103696348	103719196			+	
SLC22A5	6584	5	132369704	132395614				+
SLC25A1	6576	22	19163094	19166301			+	
SLC25A17	10478	22	41165636	41215392			+	

SLC25A20	788	3	48894356	48936402						+	
SLC25A40	55972	7	87834499	87876377					+		
SLC27A2	11001	15	50474393	50528589						+	
SLC27A4	10999	9	131102839	131123749						+	
SLC27A5	10998	19	59009700	59023432						+	
SLC2A2	6514	3	170714137	170744768						+	
SLC2A4	6517	17	7281735	7288048							+
SLC39A8	64116	4	103172198	103266655			+		+		
SLC7A10	56301	19	33699570	33716756				+			
SLCO1A2	6579	12	21417534	21548371						+	
SLCO1B1	10599	12	21284128	21392730						+	
SLCO1B3	28234	12	20963638	21069658						+	
SMARCD3	6604	7	150936059	150974231						+	
SMIM20	389203	4	25914192	25929879					+		
SNX13	23161	7	17790761	17940518					+		
SNX5	27131	20	17941596	17968991					+		
SOAT1	6646	1	179263017	179324453						+	
SORT1	6272	1	109852192	109940563	+	+					
SOX17	64321	8	54457935	54460896					+		
SPHK1	8877	17	74380690	74383941						+	
SPHK2	56848	19	49122548	49133662						+	
SPTLC1	10558	9	94793427	94877690						+	
SPTLC2	9517	14	77973270	78083110						+	
SPTLC3	55304	20	12989627	13147411						+	
SPTY2D1	144108	11	18627948	18656020			+				
SQLE	6713	8	126010720	126034525						+	
SRD5A1	6715	5	6633500	6669675						+	
SRD5A2	6716	2	31749656	31806040						+	
SRD5A3	79644	4	56212409	56237866						+	
SREBF1	6720	17	17714663	17740325							+
SREBF2	6721	22	42229106	42302375							+
ST3GAL4	6484	11	126225540	126284536			+				

STAB1	23166	3	52495338	52524496						+		
STAP1	26228	4	68424446	68473055								
STAR	6770	8	38000218	38008600							+	
STARD3	10948	17	37793333	37820454								+
STARD4	134429	5	110834022	110848157							+	
STARD5	80765	15	81605007	81616524							+	
STARD6	147323	18	51851062	51880943							+	
SUGP1	57794	19	19387322	19431307		+						
SULT2A1	6822	19	48373723	48389654							+	
TBL1X	6907	X	9431335	9687780							+	
TBL1XR1	79718	3	176738542	176915048							+	
TBL2	26608	7	72983274	72993013	+							
TBXAS1	6916	7	139478047	139720125							+	
TCF7L2	6934	10	112950219	113167678					+			
TEAD2	8463	19	49340595	49362457								+
TECR	9524	19	14640382	14676792							+	
TGS1	96764	8	56685791	56738005							+	
TIAM2	26230	6	155411423	155578857							+	
TIMD4	91937	5	156346293	156390266			+					
TM6SF2	53345	19	19264365	19273265								+
TM7SF2	7108	11	64879341	64883707							+	
TMEM176 A	55365	7	150800543	150805120						+		
TMEM97	27346	17	26646121	26655711								+
TNFRSF1B	7133	1	12167003	12209220)					+			
TNFRSF21	27242	6	47199268	47277680							+	
TOM1	10043	22	35299275	35347994						+		
TOP1	7150	20	39657462	39753127			+					
TPMT	7172	6	18128542	18155374				+				
TRIB1	10221	8	126442563	126450645	+	+						
TRIB3	57761	20	361308	378203							+	
TRPS1	7227	8	116420724	116681228			+			+		
TTC39B	158219	9	15170842	15307358			+					

TXNRD1	7296	12	104609559	104744062					+	
TYW1B	441250	7	72023729	72298813		+				
UBASH3B	84959	11	122526398	122685187		+				
UBE2L3	7332	22	21903736	21978323		+				
UCP1	7350	4	141481050	141489959					+	
UGT1A1	54658	2	233760273	233773299				+		
UGT1A9	54600	2	234580544	234681951					+	
USF1	7391	1	161039251	161045979			+			
USP3	9960	15	63796810	63883663						+
VAPA	9218	18	9913955	9960018					+	
VAPB	9217	20	56964245	57021963					+	
VDR	7421	12	48235320	48298814					+	
VEGFA	7422	6	43770209	43786487				+		
VIM	7431	10	17227935	17237593				+		
VLDLR	7436	9	2621793	2654485						+
WWOX	51741	16	78133551	79246564						+
ZBTB42	100128927	14	104800597	104804712				+		
ZHX2	22882	8	122781349	122974515						+
ZNF259	8882	11	116649276	116658739	+					
ZNF648	127665	1	182023705	182030847		+		+		
ZNF664	144348	12	124457762	124499986		+				

Chr, chromosome; Start/Stop, coordinate of chromosome start and stop of the genes/loci. This gene list includes genes associated with blood lipid concentrations (Kathiresan *et al.*, 2008; Willer *et al.*, 2008; Teslovich *et al* 2010; Brouwers *et al* 2012; Lange *et al* 2015), genes in various lipid metabolism related pathways (*NCBI Biosystems BSID 106158, 106156, 106111, 198852, 194385, 198887, 160976, 160977, 106157, 106159 and 119544), genes affected by KLF4, a master regulator of gene expression in adipose tissue (Small *et al.*, 2011), and other potential candidates from personal communications.

References:

Kathiresan S, Melander O, Guiducci C, Surti A, Burtt NP, Rieder MJ, et al. Six new loci associated with blood low-density lipoprotein cholesterol, high-density lipoprotein cholesterol or triglycerides in humans. Nature Genetics. 2008;40(2):189-97.

Willer CJ, Sanna S, Jackson AU, Scuteri A, Bonnycastle LL, Clarke R, et al. Newly identified loci that influence lipid concentrations and risk of coronary artery disease. Nature Genetics. 2008;40(2):161-9.

Teslovich TM, Musunuru K, Smith AV, Edmondson AC, Stylianou IM, Koseki M, et al. Biological, clinical and population relevance of 95 loci for blood lipids. Nature. 2010;466(7307):707-13.

Small KS, Hedman AK, Grundberg E, Nica AC, Thorleifsson G, et al. Identification of an imprinted master trans regulator at the KLF14 locus related to multiple metabolic phenotypes. Nature Genetics. 2011.

Brouwers MCGJ, van Greevenbroek MMJ, Stehouwer CDA, de Graaf J, Stalenhoef AFH. The genetics of familial combined hyperlipidaemia. Nature reviews Endocrinology. 2012;8(6):352-62.

Lange LA, Willer CJ, Rich SS. Recent developments in genome and exome-wide analyses of plasma lipids. Current opinion in lipidology. 2015;26(2):96-102.

Supplemental Table S2. A list of 104 key GWAS loci associated with plasma levels of HDL-C, or well-established candidate genes involved in HDL metabolism.

Gene	NCBI ID	Chr	Start	Stop	Kathiresan 2008	Willer 2008	Teslovich 2010	Small 2011	Brouwers 2012	Lange 2015	NCBI*	Other
ABCA1	19	9	107543283	107690436	+	+					+	
ABCA8	10351	17	66863430	66951533			+					
ABCG1	9619	21	43619799	43717354							+	
ACAA2	10449	18	47309874	47340251	+							
ADH5	128	4	99070978	99088788						+		
AKT1	207	14	104769349	104795743						+		
ALOX5	240	10	45869629	45941565							+	
AMPD3	272	11	10471868	10529126						+		
ANGPTL1	9068	1	178849535	178871353						+		
ANGPTL4	51129	19	8429011	8439257							+	
ANGPTL7	10218	1	11249346	11256038							+	
ANGPTL8	55908	19	11239619	11241943			+			+		
APOA1	335	11	116706469	116708338	+	+					+	
APOA4	337	11	116691418	116694011		+					+	
APOA5	116519	11	116660086	116663136		+					+	
APOC2	344	19	45449243	45452818		+						+
APOC3	345	11	116700624	116703787		+					+	
APOC4	346	19	45445495	45448751		+						+
APOE	348	19	45409039	45412650		+					+	
ARL15	54622	5	53180614	53606403			+			+		
ATG7	10533	3	11272324	11564704						+		
BUD13	84811	11	116618886	116643714	+							
C6orf106	64771	6	34555065	34664625			+					
CD36	948	7	80231504	80308593								*
CD93	22918	20	23059993	23066977				+				
CETP	1071	16	56995835	57017756	+	+					+	
CITED2	10370	6	139693392	139695787			+			+		
CELSR2	1952	1	109792641	109818378	+	+						

CMIP	80790	16	81478775	81745367			+			
COBLL1	22837	2	165541256	165697928			+		+	
CPS1	1373	2	210477682	210679107					+	
DAGLB	221955	7	6409116	6448012					+	
DGAT2	84649	11	75479778	75512579						+
DPEP2	64174	16	68021293	68033364						+
FADS1	3992	11	61567097	61584529						+
FADS2	9415	11	61595713	61634825			+			
FADS3	3995	11	61640998	61659006			+			
FAM13A	10144	4	88725954	89111398					+	
FTO	79068	16	53703963	54114467					+	
GALNT2	2590	1	230202956	230417875	+	+				
GOT2	2806	16	58741035	58768246						+
GSK3B	2932	3	119821321	120094417					+	
HAS1	3036	19	51713112	51723992					+	
HCAR2	338442	12	123185840	123194392						+
HDGF	3068	1	156742107	156752448					+	
HNF4A	3172	20	42984441	43061485			+			
IKZF1	10320	7	50303453	50405101					+	
IRS1	3667	2	227596033	227663506			+			
KAT5	10524	11	65711996	65719606					+	
KLF14	136259	7	130417396	130418888				+		
LACTB	114294	15	63413999	63434260			+			
LCAT	3931	16	67973787	67978015		+				+
LILRA3	11026	19	54799854	54804265			+			
LIPC	3990	15	58724175	58861073	+	+				+
LIPE	3991	19	42905666	42931578						+
LIPG	9388	18	47088427	47119278	+	+				
LPL	4023	8	19796582	19824770	+	+				+
LRP1	4035	12	57522282	57607142						+
LRP4	4038	11	46878268	46940173			+		+	
MARCH8	220972	10	45454585	45594907					+	

MC4R	4160	18	58038564	58040001						+
MLXIPL	51085	7	73007524	73038870	+	+				
MMAB	326625	12	109991520	110011358		+				
MOGAT2	80168	11	75428934	75442331					+	
MVK	4598	12	110011500	110035071		+			+	
NPC1	4864	18	21111463	21166581						+
NR1H3	10062	11	47270449	47290401					+	+
NR0B2	8431	1	27237975	27240567						+
OR4C46	119749	11	54603069	54603998				+		
OSBP	5007	11	59341871	59383617					+	
OSBPL1A	114876	18	21742009	21977790						+
PABPC4	8761	1	40026485	40042521			+	+		
PCOLCE2	26577	3	142536702	142608045						+
PDE3A	5139	12	20522179	20837041			+			
PDZK1	5174	1	145727666	145764207						+
PEPD	5184	19	33386949	33521893				+		
PGS1	9489	17	76374735	76420640			+			
PIGC	5279	1	172441457	172444090				+		
PLTP	5360	20	44527397	44540786					+	
PPP1R3B	79660	8	8993764	9009152			+			
PSRC1	84722	1	109822176	109825790	+	+				
RBM5	10181	3	50088908	50118964				+		
RSPO3	84870	6	127118671	127199481				+		
SBNO1	55206	12	123773656	123834988			+			
SCARB1	949	12	125262174	125348519					+	
SETD2	29072	3	47016408	47164109				+		
SLC39A8	64116	4	103172198	103266655			+	+		
SMIM20	389203	4	25914192	25929879				+		
SNX13	23161	7	17790761	17940518				+		
STAB1	23166	3	52495338	52524496				+		
STARD3	10948	17	37793333	37820454						+
TBL2	26608	7	72983274	72993013	+					+

TMEM176A	55365	7	150800543	150805120				+	
TRIB1	10221	8	126442563	126450645	+	+			
TRPS1	7227	8	116420724	116681228			+	+	
TTC39B	158219	9	15170842	15307358			+		
UBASH3B	84959	11	122526398	122685187			+		
UBE2L3	7332	22	21903736	21978323			+		
VEGFA	7422	6	43770209	43786487				+	
WWOX	51741	16	78133551	79246564					+
ZBTB42	100128927	14	104800597	104804712				+	
ZNF259	8882	11	116649276	116658739	+				
ZNF648	127665	1	182023705	182030847			+	+	
ZNF664	144348	12	124457762	124499986			+		

Chr, chromosome; Start/Stop, coordinate of chromosome start and stop of the genes/loci. This gene list includes genes associated with levels of HDL-C and/or HDL metabolism (Kathiresan *et al.*, 2008; Willer *et al.*, 2008; Teslovich *et al* 2010; Brouwers *et al* 2012; Lange *et al* 2015), genes in various lipid metabolism related pathways (*NCBI Biosystems BSID 106158, 106156, 106111, 198852, 194385, 198887, 160976, 160977, 106157, 106159 and 119544), genes affected by KLF4, a master regulator of gene expression in adipose tissue (Small *et al.*, 2011), and other potential candidates from personal communications.

References:

Kathiresan S, Melander O, Guiducci C, Surti A, Burtt NP, Rieder MJ, et al. Six new loci associated with blood low-density lipoprotein cholesterol, high-density lipoprotein cholesterol or triglycerides in humans. Nature Genetics. 2008;40(2):189-97.

Willer CJ, Sanna S, Jackson AU, Scuteri A, Bonnycastle LL, Clarke R, et al. Newly identified loci that influence lipid concentrations and risk of coronary artery disease. Nature Genetics. 2008;40(2):161-9.

Teslovich TM, Musunuru K, Smith AV, Edmondson AC, Stylianou IM, Koseki M, et al. Biological, clinical and population relevance of 95 loci for blood lipids. Nature. 2010;466(7307):707-13.

Small KS, Hedman AK, Grundberg E, Nica AC, Thorleifsson G, et al. Identification of an imprinted master trans regulator at the KLF14 locus related to multiple metabolic phenotypes. Nature Genetics. 2011.

Brouwers MCGJ, van Greevenbroek MMJ, Stehouwer CDA, de Graaf J, Stalenhoef AFH. The genetics of familial combined hyperlipidaemia. Nature reviews Endocrinology. 2012;8(6):352-62.

Lange LA, Willer CJ, Rich SS. Recent developments in genome and exome-wide analyses of plasma lipids. Current opinion in lipidology. 2015;26(2):96-102.

Supplemental Table S3. Rare heterozygous damaging variants among all lipid metabolism candidate genes.

Gene	Chr:Pos:Ref:Alt	AA change	gnomAD	ID	ClinVar Significance	ClinVar Disease	DP*	Prediction	Number of Patients
A2M	12-9262616-G>T	p.Arg174Ser	0.00E+00	.	.	.	9	PD	1
A2ML1	12-9009835-T>C	p.Met975Thr	6.00E-04	rs202179061	.	.	8	PD	1
A2ML1	12-9020954-G>A	splice variant	1.00E-03	rs202067416	.	.	10	PD	3
ABCA1	9-107556776-T>G	p.Asn1800His	3.00E-04	rs146292819	Pathogenic	ABCA1-Related Disorders	9	Pathogenic	1
ABCA1	9-107560784-C>T	p.Arg1680Gln	3.00E-04	rs150125857	Likely benign|Likely benign	Tangier disease|Familial High Density Lipoprotein Deficiency	9.5	PD	1
ABCA1	9-107566964-G>A	p.Thr1501Ile	0.00E+00	.	.	.	9.5	PD	1
ABCA1	9-107568536-G>GC	p.Leu1484fs	0.00E+00	.	.	.	10	PD	1
ABCA1	9-107571799-G>A	p.Leu1408Phe	4.00E-04	rs201879964	Likely benign|Likely benign	Tangier disease|Familial High Density Lipoprotein Deficiency	6.5	PD	1
ABCA1	9-107574868-C>T	p.Gly1346Glu	1.00E-04	rs762770081	Likely pathogenic	Inborn genetic diseases	9.5	PD	1
ABCA1	9-107574881-G>A	p.Arg1342Trp	1.62E-05	rs760786920	Uncertain significance|Uncertain significance	Tangier disease|Familial High Density Lipoprotein Deficiency	9.5	PD	1
ABCA1	9-107578437-G>A	p.Thr1242Met	2.03E-05	rs144923927	.	.	10	PD	1
ABCA1	9-107578515-C>A	p.Gly1216Val	4.47E-05	rs562403512	.	.	9.5	PD	1
ABCA1	9-107583758-G>A	p.Thr953Ile	0.00E+00	.	.	.	9.5	PD	1
ABCA1	9-107584879-C>CGGTA	p.Arg909fs	0.00E+00	.	.	.	10	PD	1
ABCA1	9-107587972-A>G	p.Val845Ala	3.25E-05	rs541344598	.	.	8	PD	1
ABCA1	9-107593272-G>A	p.Thr609Met	3.25E-05	rs755276277	.	.	9	PD	1
ABCA1	9-107593329-C>T	p.Trp590*	0.00E+00	.	.	.	10	PD	1
ABCA1	9-107594878-G>A	p.Arg496Trp	6.00E-04	rs147675550	Likely benign|Likely benign	Familial High Density Lipoprotein Deficiency|Tangier disease	7	PD	1
ABCA1	9-107599263-G>A	p.Arg437Trp	4.87E-05	rs150448790	.	.	9.5	PD	1
ABCA1	9-107599797-C>T	p.Arg369His	2.44E-05	rs370223805	.	.	9.5	PD	1
ABCA1	9-107602623-T>C	p.Lys331Glu	0.00E+00	.	.	.	7.5	PD	1
ABCA1	9-107646756-G>A	p.Pro85Leu	1.40E-03	rs145183203	Likely benign|Likely benign	Tangier disease|Familial High Density Lipoprotein Deficiency	8.5	PD	1
ABCA1	9-107665929-A>G	p.Leu11Pro	4.84E-06	rs777372679	.	.	10	PD	1

ABCA8	17-66872640-G>A	p.Pro1444Leu	5.69E-05	rs372083205	.	.	7	PD	1
ABCA8	17-66878099-C>T	p.Cys1284Tyr	8.70E-03	rs34987539	.	.	9.4	PD	3
ABCA8	17-66902302-G>T	p.Leu761Ile	1.80E-03	rs140010342	.	.	9	PD	1
ABCA8	17-66915549-C>T	p.Gly561Arg	5.00E-04	rs552291569	.	.	7.5	PD	1
ABCB11	2-169783728-C>T	p.Glu1186Lys	2.10E-03	rs1521808	other|Benign	not provided|not specified	8.3	PD	1
ABCB11	2-169787251-A>C	p.Val1112Gly	0.00E+00	.	.	.	10	PD	1
ABCB11	2-169828509-C>A	p.Asp496Tyr	0.00E+00	.	.	.	8.5	PD	1
ABCB4	7-87031582-G>A	p.Arg1231Cys	1.63E-05	rs375484907	.	.	9	PD	1
ABCB4	7-87049345-C>T	p.Arg788Gln	5.90E-03	rs8187801	Benign	not specified	7.8	PD	1
ABCB4	7-87060844-C>T	p.Arg590Gln	4.50E-03	rs45575636	Pathogenic|Pathogenic|other	Cholestasis, intrahepatic, of pregnancy 3|Cholecystitis|not specified	9.4	Pathogenic	3
ABCC1	16-16126970-C>G	p.Pro207Arg	0.00E+00	.	.	.	9.5	PD	1
ABCC1	16-16139716-A>C	p.Asp355Ala	0.00E+00	.	.	.	7.5	PD	1
ABCC1	16-16142079-G>T	p.Arg433Ser	8.80E-03	rs60782127	.	.	8.9	PD	5
ABCC1	16-16200716-G>C	p.Ala963Pro	1.00E-03	rs201798499	.	.	8.5	PD	2
ABCC1	16-16208889-G>A	p.Ala1126Thr	1.50E-03	rs200039403	.	.	6.5	PD	1
ABCC1	16-16218656-G>A	p.Val1211Met	2.03E-05	rs560033743	.	.	7.5	PD	1
ABCC2	10-101544535-GC>G	p.Gln69fs	0.00E+00	.	.	.	10	PD	1
ABCC2	10-101565157-A>G	p.Lys495Glu	4.10E-03	rs17222561	Benign	not specified	10	PD	1
ABCC2	10-101591737-T>C	p.Ile1036Thr	5.00E-03	rs45441199	Benign	not specified	6.5	PD	2
ABCC2	10-101594176-C>A	p.Arg1100Ser	1.00E-04	rs142715085	.	.	8	PD	1
ABCC2	10-101595975-G>T	p.Arg1181Leu	7.40E-03	rs8187692	Benign|Likely benign	not specified|Dubin-Johnson syndrome	7.2	PD	1
ABCC2	10-101604107-C>T	p.Pro1291Leu	2.80E-03	rs17216317	Benign	not specified	9.4	PD	1
ABCC2	10-101610448-A>T	p.Asp1468Val	0.00E+00	.	.	.	10	PD	1
ABCC3	17-48741440-C>A	p.Gln436Lys	9.00E-04	rs151079073	.	.	9.5	PD	1
ABCC3	17-48746550-G>A	p.Val663Met	6.21E-05	rs142343463	.	.	9.5	PD	1
ABCC3	17-48750403-T>G	p.Ile771Met	1.00E-04	rs373386354	.	.	7	PD	1
ABCC3	17-48753127-C>CATTGG	p.Thr953fs	0.00E+00	.	.	.	10	PD	1
ABCC3	17-48755127-G>A	p.Arg1134Gln	2.00E-04	rs200903266	.	.	10	PD	1
ABCC3	17-48761062-C>T	p.Pro1300Leu	4.00E-04	rs41280128	.	.	8	PD	1

ABCC3	17-48762223-G>A	p.Gly1423Arg	1.40E-03	rs148982238	.	.	10	PD	1
ABCC3	17-48764898-G>A	p.Val1428Met	3.00E-04	rs532140025	.	.	8	PD	1
ABCC3	17-48764950-G>A	p.Arg1445His	9.35E-05	rs143817593	.	.	9.5	PD	1
ABCG5	2-44055163-C>T	p.Arg198Gln	1.20E-03	rs141828689	Uncertain significance	not specified	9	PD	1
ABCG5	2-44059195-G>C	p.Ala98Gly	2.40E-03	rs145164937	Benign	not specified	9.5	PD	1
ABCG5	2-44065003-C>T	p.Gly79Arg	4.00E-04	rs142125966	.	.	7.5	PD	1
ABCG8	2-44079765-C>T	p.Ser241Phe	3.00E-04	rs547583131	.	.	9	PD	1
ABCG8	2-44099233-G>A	p.Trp361*	9.00E-04	rs137852987	Pathogenic|Pathogenic	Sitosterolemia|not provided	10	Pathogenic	1
ABHD5	3-43743914-G>T	p.Arg114Leu	7.80E-03	rs148743497	.	.	8.3	PD	2
ACAA2	18-47317912-G>A	p.His271Tyr	8.00E-04	rs148304029	.	.	7	PD	1
ACAA2	18-47323888-G>T	p.Thr87Lys	3.25E-05	rs531271159	.	.	10	PD	1
ACAA2	18-47329071-C>G	p.Gly57Arg	9.35E-05	rs141307346	.	.	10	PD	1
ACACA	17-35454892-C>T	p.Arg2198Gln	2.00E-04	rs149967550	.	.	8	PD	1
ACACB	12-109609695-C>G	p.Asp337Glu	1.62E-05	rs779523944	.	.	8	PD	2
ACACB	12-109623477-C>A	p.Pro638Thr	0.00E+00	.	.	.	7.5	PD	1
ACACB	12-109654433-G>A	p.Gly1121Arg	2.03E-05	rs765703397	.	.	9.5	PD	1
ACACB	12-109683531-A>G	p.Asn1760Ser	1.30E-03	rs147443159	.	.	8	PD	1
ACACB	12-109684039-G>A	p.Arg1786Gln	4.30E-03	rs144673785	.	.	10	PD	3
ACAD11	3-132280031-G>A	p.Arg678Cys	1.63E-05	rs143271405	.	.	10	PD	1
ACAD11	3-132294680-G>C	p.Ala646Gly	6.00E-04	rs36121581	.	.	8	PD	1
ACAD11	3-132322103-C>A	p.Val531Leu	2.00E-04	rs767280144	.	.	7.5	PD	1
ACADL	2-211081090-T>A	p.Thr173Ser	2.85E-05	rs747282926	.	.	8	PD	1
ACADM	1-76198337-G>A	p.Glu43Lys	2.10E-03	rs147559466	Pathogenic|other|Benign	not provided|Medium-chain acyl-coenzyme A dehydrogenase deficiency|not specified	8.5	Pathogenic	1
ACADM	1-76198409-T>C	p.Tyr67His	5.00E-04	rs121434280	Pathogenic|Pathogenic	Medium-chain acyl-coenzyme A dehydrogenase deficiency|not provided	6	Pathogenic	1
ACADM	1-76226846-A>G	p.Lys362Glu	3.30E-03	rs77931234	Pathogenic,Pathogenic|Pathogenic	Medium-chain acyl-coenzyme A dehydrogenase deficiency,Medium-chain acyl-coenzyme A dehydrogenase deficiency|not provided	6	Pathogenic	1
ACADS	12-121174897-C>T	p.Arg107Cys	1.00E-03	rs61732144	Pathogenic|Pathogenic	Deficiency of butyryl-CoA dehydrogenase|not provided	10	Pathogenic	1

ACADS	12-121176140-G>A	p.Glu228Lys	2.44E-05	rs755247580	.	.	9.5	PD	1
ACLY	17-40028410-T>A	p.Ile890Phe	1.00E-03	rs41275673	.	.	7	PD	1
ACLY	17-40049386-C>T	p.Val501Met	1.00E-04	rs151030814	.	.	8	PD	1
ACOT8	20-44473029-CA>C (1 homozygote)	p.Leu173fs	9.00E-04	rs751916570	.	.	10	PD	2
ACOT8	20-44485952-C>CATCT (1 homozygote)	p.Met1fs	1.56E-02	rs201025211	.	.	10	PD	3
ACOX2	3-58516326-G>A	p.Gln287*	8.13E-06	rs765042842	.	.	10	PD	1
ACOX2	3-58517450-G>A	p.Arg225Trp	3.00E-04	rs150832314	.	.	9	PD	1
ACOX2	3-58519731-CTCTG>C	p.Thr154fs	2.30E-03	rs34391522	.	.	10	PD	1
ACOX3	4-8391455-C>T	p.Arg436Gln	2.00E-04	rs199993833	.	.	7	PD	1
ACSL6	5-131308521-G>A	p.Arg412Cys	4.00E-04	rs140341663	.	.	8	PD	1
ADAM28	8-24157563-AAGAG>A	p.Glu43fs	1.22E-05	rs778896961	.	.	10	PD	1
AGPAT1	6-32137118-G>A	p.Arg263Trp	2.08E-05	rs139093368	.	.	7	PD	1
AGPAT2	9-139571430-G>A	p.Arg159Cys	5.20E-03	rs142993240	Likely benign	not specified	7.5	PD	6
AGPAT3	21-45390673-GC>G	p.Leu218fs	0.00E+00	.	.	.	10	PD	1
AGPAT4	6-161587296-C>T	p.Arg111His	0.00E+00	.	.	.	9.5	PD	1
AGPAT5	8-6588258-G>T	p.Ala106Ser	1.10E-03	rs145228795	.	.	9	PD	1
AGPAT6	8-41469728-G>C	p.Cys244Ser	0.00E+00	.	.	.	9	PD	1
AGT	1-230840034-G>T	p.Leu392Met	1.30E-03	rs1805090	.	.	7.5	PD	1
AGT	1-230846190-G>A	p.Pro136Leu	4.06E-06	.	.	.	9.5	PD	1
AKR1B1	7-134130068-AAGACCT>A	p.Val276fs	4.13E-06	rs758050561	.	.	10	PD	1
AMPD3	11-10508903-G>T	p.Val320Leu	6.80E-03	rs117706710	Benign	not specified	7.8	PD	1
AMPD3	11-10515022-C>T	p.Arg365Trp	3.00E-04	rs149809940	.	.	8.5	PD	1
AMPD3	11-10515088-G>A	p.Ala387Thr	4.11E-05	rs767255627	.	.	9.5	PD	1
AMPD3	11-10517141-A>G	p.Ser440Gly	1.22E-05	rs763919044	.	.	7.5	PD	1
AMPD3	11-10517252-C>T	p.Arg477Cys	6.09E-05	rs147091692	.	.	9.5	PD	1
ANGPTL3	1-63063592-GAACTC>G	p.Asn121fs	3.00E-04	rs569107562	Pathogenic	Hypobetalipoproteinemia, familial, 2	10	Pathogenic	1
ANGPTL3	1-63069818-CA>C	p.Asn371fs	0.00E+00	.	.	.	10	PD	1
ANGPTL7	1-11253684-G>T	p.Gln175His	3.40E-03	rs28991009	.	.	9.5	PD	1
ANGPTL7	1-11253817-C>T	p.Arg220Cys	4.50E-03	rs147660927	.	.	8.3	PD	1
ANKRD1	10-92678626-T>A	p.Asp150Val	7.35E-05	rs150266349	Uncertain significance	not specified	7.5	PD	1

APOA1	11-116706768-A>C	p.Leu187Arg	0.00E+00	.	.	.	9.5	PD	1
APOA1	11-116707739-A>C	p.Ser60Ala	3.00E-04	rs199759119	.	.	7	PD	1
APOA1	11-116707831-T>TG	p.Gln29fs	1.63E-05	rs753348565	.	.	10	PD	1
APOB	2-21225264-CAT>C	p.Tyr4343fs	1.68E-05	rs760832994	.	.	10	PD	1
APOB	2-21229068-G>A	p.Arg3558Cys	3.00E-04	rs12713559	Pathogenic|Uncertain significance|Uncertain significance|Uncertain significance|Pathogenic	Familial hypercholesterolemia|not specified|Hypercholesterolemia, autosomal dominant, type B|Hypobetalipoproteinemia, familial, 1|Hypercholesterolemia, autosomal dominant, type B	8.5	Pathogenic	1
APOB	2-21229160-C>T	p.Arg3527Gln	3.00E-04	rs5742904	other|Pathogenic|Uncertain significance|Pathogenic	Familial hypercholesterolemia|not provided|Familial hypobetalipoproteinemia|Hypercholesterolemia, autosomal dominant, type B	8.5	Pathogenic	1
APOB	2-21233902-G>T	p.Tyr1946*	0.00E+00	.	.	.	10	PD	1
APOB	2-21235089-G>A	p.Gln1551*	0.00E+00	rs142017360	.	.	10	PD	1
APOB	2-21260829-C>A	splice variant	0.00E+00	.	.	.	10	PD	1
APOBEC1	12-7803617-A>C	splice variant	4.12E-06	rs373316326	.	.	10	PD	1
APOBEC1	12-7805414-C>T	p.Trp21*	5.90E-03	rs34275479	.	.	10	PD	3
APOBEC1	12-7818468-T>C	p.Met1?	1.60E-03	rs139646668	.	.	10	PD	4
APOC3	11-116701354-G>A	splice variant	1.40E-03	rs138326449	other|Pathogenic	Coronary heart disease|Hyperalphalipoproteinemia 2	10	Pathogenic	1
APOD	3-195300740-C>T	p.Val104Met	9.70E-03	rs76929107	.	.	7.8	PD	1
APOE	19-45412358-C>G	p.Arg269Gly	4.00E-04	rs267606661	Pathogenic	Familial type 3 hyperlipoproteinemia	5.5	PD	1
ATG4C	1-63299729-TTG>T	p.Val320fs	1.90E-03	rs551767003	.	.	10	PD	1
ATG4C	1-63329789-C>T	p.Gln446*	2.00E-04	rs200697610	.	.	10	PD	2
ATG7	3-11389502-C>T	p.Pro426Leu	1.20E-03	rs143545741	.	.	9	PD	2
BCMO1	16-81298282-C>T	p.Thr170Met	1.40E-03	rs119478057	Pathogenic	Hypercarotenemia and vitamin a deficiency, autosomal dominant	9.5	Pathogenic	1
BCMO1	16-81301689-C>T	p.Arg266Trp	1.00E-04	rs148510879	.	.	8.5	PD	1
BCMO1	16-81323953-G>C	p.Gly472Ala	6.00E-03	rs143238312	.	.	10	PD	4
BDH1	3-197241251-G>A	p.Thr149Met	8.19E-05	rs199764274	.	.	8	PD	2
BMP1	8-22054765-G>A	p.Asp647Asn	8.14E-06	.	.	.	7	PD	1

BRCA2	13-32914437-GT>G	p.Ser1982fs	3.00E-04	rs80359550	Pathogenic|other|Pathogenic|Pathogenic|Pathogenic|Pathogenic|Pathogenic|Pathogenic|Pathogenic	Breast-ovarian cancer, familial 2|Pancreatic cancer 2|Fanconi anemia, complementation group D1|Hereditary breast and ovarian cancer syndrome|Familial cancer of breast|Hereditary cancer-predisposing syndrome|not provided|BRCA2-Related Disorders|Breast cancer	10	Pathogenic	1
BRCA2	13-32915190-C>A	p.Ala2233Asp	8.13E-06	rs41293501	Uncertain significance|Uncertain significance|Uncertain significance|Uncertain significance	Hereditary breast and ovarian cancer syndrome|Breast-ovarian cancer, familial 2|Hereditary cancer-predisposing syndrome|not specified	8.5	PD	1
BRCA2	13-32930613-T>C	p.Ile2495Thr	8.12E-06	rs80358974	not provided|other|Uncertain significance|Uncertain significance	Familial cancer of breast|Breast-ovarian cancer, familial 2|Hereditary cancer-predisposing syndrome|Hereditary breast and ovarian cancer syndrome	8.5	PD	1
BRCA2	13-32954037-A>C	p.Tyr3035Ser	5.32E-05	rs80359165	other|Likely benign|Likely benign|Likely benign,Uncertain significance|Uncertain significance|Uncertain significance|Uncertain significance,Uncertain significance	Breast-ovarian cancer, familial 2|Hereditary breast and ovarian cancer syndrome|not specified|Hereditary cancer-predisposing syndrome,not specified|Breast-ovarian cancer, familial 2|Hereditary breast and ovarian cancer syndrome|Hereditary cancer-predisposing syndrome,Hereditary cancer-predisposing syndrome	7.5	PD	1
BRCA2	13-32972626-A>T (1 homozygote)	p.Lys3326*	6.60E-03	rs11571833	Benign|Benign|other|Benign|Benign|Benign	Breast-ovarian cancer, familial 2|not provided|Hereditary breast and ovarian cancer syndrome|not specified|Hereditary cancer-predisposing syndrome|Familial cancer of breast	10	PD	3
CACNG5	17-64873530-C>T	p.Ala27Val	3.66E-05	rs772824396	.	.	8.5	PD	1
CACNG5	17-64876814-G>A	p.Gly142Ser	1.63E-05	rs755762556	.	.	9.4	PD	1
CAPN3	15-42681126-G>C	p.Lys211Asn	0.00E+00	.	Uncertain significance	not specified	10	PD	1
CAPN3	15-42691740-A>G	p.Asn415Ser	2.44E-05	rs769367343	.	.	8.5	PD	1
CAPN3	15-42693952-C>T	p.Arg490Trp	9.35E-05	rs141656719	Pathogenic|Pathogenic|Pathogenic	not provided|Limb-girdle muscular dystrophy, type 2A|not provided	9	Pathogenic	1
CAV2	7-116140446-C>A	p.Pro95Thr	2.03E-05	rs754623381	.	.	9.5	PD	1

CCDC121	2-27849928-GTC>G	p.Arg408fs	9.70E-03	rs199568016	.	.	10	PD	5
CD36	7-80285989-T>G	p.Val85Gly	0.00E+00	.	.	.	8.5	PD	1
CD36	7-80286003-C>T	p.Pro90Ser	1.10E-03	rs75326924	Pathogenic	Platelet glycoprotein IV deficiency	10	Pathogenic	1
CD36	7-80300449-T>G	p.Tyr325*	6.00E-03	rs3211938	.	.	10	PD	3
CD36	7-80302113-AAACGGCTGCAG>A	p.Lys385fs	8.17E-06	.	.	.	10	PD	1
CD36	7-80302672-GTATT>G	p.Val401fs	1.00E-04	rs769354931	.	.	10	PD	1
CD93	20-23065998-C>T	p.Gly278Arg	2.00E-04	rs150125306	.	.	8	PD	1
CD93	20-23066727-C>A	p.Ala35Ser	1.33E-05	rs765091675	.	.	7	PD	1
CELSR2	1-109808789-G>A	p.Gly1992Arg	7.00E-04	rs12567377	.	.	8.5	PD	1
CERS1	19-18990163-C>T	p.Val263Ile	5.00E-04	rs200539084	.	.	7	PD	1
CERS5	12-50561023-G>A	p.Arg20Cys	5.40E-03	rs143484198	.	.	8.9	PD	1
CHD9	16-53301919-G>A	p.Arg1533Gln	4.07E-06	rs764877001	.	.	9.5	PD	1
CHD9	16-53302040-T>C	splice variant	9.35E-06	rs772704591	.	.	10	PD	1
CHD9	16-53321892-A>G	p.Lys1738Arg	2.70E-03	rs61754093	.	.	7.5	PD	2
CMTM5	14-23847975-C>CCG	p.Trp128fs	3.20E-03	rs367613599	.	.	10	PD	1
COBLL1	2-165578958-G>T	p.Arg312Ser	5.45E-06	rs553945564	.	.	7.5	PD	1
COL18A1	21-46925126-C>T	p.Arg1398Cys	4.00E-04	rs372973695	.	.	6.5	PD	1
COL18A1	21-46925127-G>A	p.Arg1398His	1.00E-03	rs200299380	.	.	6.5	PD	1
COL18A1	21-46925285-CGGCCCTCCGGGCCCCCCTG>C	p.Pro1427fs	1.26E-05	.	.	.	10	PD	1
CPS1	2-211421462-C>T	p.Thr8Met	1.00E-04	rs150314086	Uncertain significance	Congenital hyperammonemia, type I	7	PD	1
CPS1	2-211442212-G>A	p.Gly156Glu	6.40E-03	rs114819130	Uncertain significance	Congenital hyperammonemia, type I	8.3	PD	4
CPS1	2-211454831-G>A	p.Arg244Gln	3.00E-04	rs147294932	.	.	8	PD	1
CPS1	2-211466932-G>A	p.Asp578Asn	1.10E-03	rs142916171	.	.	8.5	PD	1
CPS1	2-211473128-C>A	p.Pro752Thr	3.26E-05	rs139740322	.	.	8	PD	1
CPT1A	11-68552391-C>T	p.Arg352Gln	2.60E-03	rs374383052	.	.	7.8	PD	1
CPT1A	11-68562288-C>T	p.Arg288Gln	5.30E-03	rs140958507	Uncertain significance	not specified	8.5	PD	2
CPT1B	22-51008055-C>T	p.Glu729Lys	2.00E-04	rs114814733	.	.	8.5	PD	1
CPT1B	22-51011322-T>A	p.His445Leu	4.11E-05	rs144799109	.	.	9	PD	1
CPT1B	22-51011373-T>C	p.Tyr428Cys	9.00E-04	rs141820211	.	.	9.5	PD	1

CPT1B	22-51014671-G>T	p.Leu219Met	1.63E-05	rs373379091	.	.	9	PD	1
CPT2	1-53668099-C>T	p.Ser113Leu	1.40E-03	rs74315294	Pathogenic|Pathogenic|Pathogenic|Pathogenic	CARNITINE PALMITOYLTRANSFERASE II DEFICIENCY, MYOPATHIC, STRESS-INDUCED|not provided|Carnitine palmitoyltransferase II deficiency, lethal neonatal|Carnitine palmitoyltransferase II deficiency	9.5	Pathogenic	1
CPT2	1-53675699-A>G	p.Asp118Gly	2.10E-03	rs148035648	Uncertain significance	not specified	10	PD	1
CPT2	1-53675846-G>A	p.Arg167Gln	2.00E-04	rs144760921	.	.	6.5	PD	1
CREBBP	16-3781845-G>T	p.Pro1608Thr	4.00E-04	rs73491901	Benign	not specified	7.5	PD	1
CREBBP	16-3786158-T>C	p.Glu1536Gly	4.06E-06	rs776332449	.	.	9.5	PD	1
CREBBP	16-3788632-C>T	p.Arg1441Gln	2.44E-05	rs756756076	.	.	7	PD	1
CREBBP	16-3900713-G>C	p.Ser128Cys	7.00E-04	rs55790011	Likely benign	not specified	7	PD	2
CREBBP	16-3900803-C>A	p.Gly98Val	5.00E-04	rs141982003	not provided	not specified	8.9	PD	1
CROT	7-86978458-T>C	p.Val25Ala	5.30E-03	rs143103247	.	.	8.9	PD	1
CROT	7-86986846-AG>A	p.Gly48fs	6.00E-04	rs756304697	.	.	10	PD	1
CROT	7-87005208-G>A	p.Ser300Asn	4.00E-04	rs138191847	.	.	10	PD	1
CUBN	10-16911749-C>T	p.Gly3114Ser	6.70E-03	rs117035284	.	.	7.2	PD	2
CUBN	10-16918923-C>T	p.Gly3027Arg	2.00E-04	rs150202444	.	.	7	PD	1
CUBN	10-17171762-C>A	p.Met1?	2.00E-04	rs11254385	Likely pathogenic	not provided	10	Pathogenic	1
CYP11A1	15-74640307-CG>C	p.Arg120fs	4.06E-06	.	.	.	10	PD	1
CYP19A1	15-51503214-G>A	p.Arg435Cys	1.22E-05	rs121434534	Pathogenic	Aromatase deficiency	9.5	Pathogenic	1
CYP1A1	15-75012979-G>T	p.Arg464Ser	4.30E-03	rs41279188	.	.	7.5	PD	3
CYP1A1	15-75012997-TA>T	p.Cys457fs	9.00E-04	rs561096394	.	.	10	PD	3
CYP1A1	15-75014027-A>G	p.Ile286Thr	1.50E-03	rs4987133	.	.	8.5	PD	1
CYP1A1	15-75014718-G>A	p.Arg241Cys	7.72E-05	rs149687459	.	.	8.5	PD	1
CYP1A1	15-75014727-G>A	p.Pro238Ser	1.40E-03	rs61747605	.	.	8.5	PD	1
CYP1A1	15-75015122-CG>C	p.Arg106fs	5.00E-04	rs779460432	.	.	10	PD	2
CYP1A1	15-75015162-G>A	p.Arg93Trp	7.00E-04	rs2229150	.	.	8	PD	1
CYP1A2	15-75042296-G>A	p.Gly73Arg	3.00E-04	rs45565238	.	.	9	PD	1
CYP1A2	15-75044552-G>A	p.Arg377Gln	0.00E+00	rs72547515	.	.	9	PD	1
CYP26A1	10-94834140-G>T	p.Val89Leu	0.00E+00	.	.	.	7	PD	1

CYP26A1	10-94836739-G>A	p.Gly391Asp	4.00E-04	rs547906104	.	.	8.5	PD	1
CYP27A1	2-219679419-G>C	p.Gly472Ala	9.35E-05	rs200883871	Pathogenic	Cholestanol storage disease	10	Pathogenic	1
CYP27B1	12-58159789-C>T	splice variant	5.89E-06	rs770204470	.	.	10	PD	1
CYP2B6	19-41510297-G>T	p.Glu144*	5.69E-05	rs117323987	.	.	10	PD	1
CYP39A1	6-46554899-G>A	p.Arg389Cys	1.30E-03	rs141809814	.	.	9.5	PD	1
CYP4A11	1-47395863-A>C	p.Val496Gly	4.88E-05	rs562189386	.	.	9	PD	1
CYP4A11	1-47399944-G>A	p.Ser332Phe	8.00E-04	rs141672858	.	.	9	PD	1
CYP4B1	1-47280785-G>A	p.Ala308Thr	1.80E-03	rs144531409	.	.	7.5	PD	1
DAGLB	7-6449535-AC>A	p.Val651fs	0.00E+00	.	.	.	10	PD	1
DEGS1	1-224377810-G>T	p.Gly205Val	0.00E+00	.	.	.	7	PD	1
DHCR24	1-55337283-C>T	p.Glu206Lys	6.00E-04	rs150688144	.	.	9	PD	2
DHCR7	11-71146639-G>A	p.Arg404Cys	4.21E-05	rs61757582	Pathogenic	Smith-Lemli-Opitz syndrome	10	Pathogenic	1
DHCR7	11-71146861-C>T	p.Val330Met	4.00E-04	rs139724817	.	.	8.5	PD	1
DHCR7	11-71148980-C>T	p.Val281Met	1.00E-04	rs398123607	Pathogenic|Pathogenic	not provided|Smith-Lemli-Opitz syndrome	10	Pathogenic	1
DHCR7	11-71152447-C>T	p.Trp151*	8.00E-04	rs11555217	Pathogenic|other|Pathogenic|Pathogenic|Pathogenic|Pathogenic	Smith-Lemli-Opitz syndrome|not provided|2-3 toe syndactyly|Congenital microcephaly|Elevated 7-dehydrocholesterol|Small for gestational age	10	Pathogenic	1
DOCK7	1-63005305-T>C	p.Asn1071Asp	9.38E-05	rs371894094	.	.	8.5	PD	1
ECHS1	10-135184105-A>G	p.Val82Ala	4.19E-06	.	.	.	8	PD	1
ECI1	16-2293428-C>T	p.Ala152Thr	4.07E-06	.	.	.	9.5	PD	1
ECI1	16-2296877-C>T	p.Gly93Ser	8.80E-03	rs141206306	.	.	9.4	PD	1
EHBP1	2-63101587-C>G	p.Pro404Ala	2.70E-03	rs150973298	.	.	7.5	PD	1
EHBP1	2-63175645-A>T	p.Asp590Val	4.30E-03	rs77187983	.	.	8.5	PD	3
ELOVL2	6-11000362-TAA>T	p.Leu97fs	0.00E+00	.	.	.	10	PD	1
ELOVL5	6-53152683-G>A	p.Gln102*	6.30E-03	rs150583340	.	.	10	PD	2
ELOVL7	5-60063730-A>AAT	splice variant	0.00E+00	.	.	.	10	PD	1
FAM13A	4-89660218-T>A	p.Lys842Ile	0.00E+00	.	.	.	9.5	PD	1
FASN	17-80046132-C>T	p.Gly882Asp	2.00E-04	rs371010865	.	.	10	PD	1
FHL2	2-105979834-C>T	p.Arg315His	8.14E-06	rs756076159	.	.	8.5	PD	1

FHL2	2-105984190-C>T	p.Arg229His	4.90E-05	rs752531815	.	.	9.5	PD	1	
FN1	2-216286888-C>A	p.Gly491Val	4.06E-06	rs374421953	.	.	7.5	PD	1	
FN1	2-216289015-C>T	p.Gly357Glu	2.40E-03	rs140926439	.	.	9.5	PD	1	
FRK	6-116265493-G>T	p.His352Asn	1.00E-04	rs201586564	.	.	10	PD	1	
FTO	16-53859930-C>G	p.Pro93Arg	1.00E-04	rs151263395	.	.	7.5	PD	2	
GC	4-72631182-G>A	p.Ala166Val	1.00E-04	rs116134435	.	.	8	PD	1	
GCKR	2-27722311-ACT>A	p.Ser183fs	3.00E-04	rs768048286	.	.	10	PD	1	
GCKR	2-27730169-C>CA	p.Thr379fs	1.20E-03	rs573498430	.	.	10	PD	1	
GCKR	2-27731033-A>C	splice variant	8.13E-06	rs199605744	.	.	10	PD	1	
GCKR	2-27741787-G>A	p.Ala519Thr	3.00E-04	rs144127332	.	.	7.5	PD	1	
GLB1	3-33060001-G>A	p.Pro477Leu	2.00E-04	rs201148460	.	.	8.5	PD	1	
GOT2	16-58742107-G>C	p.Leu421Val	0.00E+00	.	.	.	7	PD	1	
GOT2	16-58743431-T>G	p.Met354Leu	0.00E+00	.	.	.	7.5	PD	1	
GOT2	16-58743442-C>T	p.Arg350His	2.44E-05	rs148828634	.	.	9	PD	1	
GOT2	16-58752439-C>T	p.Asp197Asn	8.00E-04	rs149988435	.	.	9	PD	1	
GOT2	16-58753152-G>A	p.Thr128Ile	1.62E-05	rs746079905	.	.	7.5	PD	1	
GPD1	12-50501413-G>A	p.Ala226Thr	3.25E-05	rs751127919	.	.	7	PD	1	
GPD2	2-157369961-C>T	p.Pro205Leu	1.30E-03	rs142821701	.	.	10	PD	2	
GPD2	2-157407168-C>G	p.Thr294Arg	0.00E+00	.	.	.	9.5	PD	1	
GRHL1	2-10101170-TAAAG>T	p.Arg93 Asn94delinsSer	0.00E+00	.	.	.	10	PD	1	
GRIN3A	9-104433256-G>C	p.Arg480Gly	1.40E-03	rs149729514	.	.	6.7	PD	1	
HACL1	3-15609939-C>CA	p.Arg417fs	1.33E-05	rs769201368	.	.	10	PD	1	
HADH	4-108935600-T>G	p.Phe151Cys	5.50E-03	rs61735992	Uncertain significance|other	not specified|not provided	8.5	PD	2	
HADHA	2-26418053-C>G	p.Glu510Gln	1.30E-03	rs137852769	Pathogenic|Pathogenic|Pathogenic|Pathogenic	Mitochondrial trifunctional protein deficiency|Lchad deficiency with maternal acute fatty liver of pregnancy|Long-chain 3-hydroxyacyl-CoA dehydrogenase deficiency|not provided	10	Pathogenic	1	
HADHA	2-26453084-C>G	p.Val218Leu	9.00E-04	rs71441018	.	.	8	PD	1	
HAS1	19-52216738-C>T	p.Trp567*	2.00E-04	rs200444967	.	.	10	PD	1	

HMGCR	5-74640082-T>A	p.Leu97His	4.54E-05	rs758983271	.	.	9	PD	1
HMGCR	5-74645921-A>G	p.Asn204Ser	4.00E-04	rs148335635	.	.	9.5	PD	1
HMGCS1	5-43298200-A>G	p.Val162Ala	2.85E-05	rs770255909	.	.	9.5	PD	1
HMGCS2	1-120295237-C>T	p.Arg452Gln	4.06E-06	rs770800317	.	.	10	PD	1
HNF1A	12-121426776-C>T	p.Thr156Met	6.91E-05	rs150513055	Likely pathogenic	Maturity-onset diabetes of the young, type 3	9.5	Pathogenic	1
HNF1A	12-121426790-G>A	p.Ala161Thr	9.34E-05	rs201095611	.	.	9.5	PD	1
HNF1A	12-121434488-G>A	p.Glu418Lys	0.00E+00	.	.	.	7	PD	1
HNF1A	12-121437410-G>A	p.Arg614Gln	5.00E-04	rs137853242	Pathogenic	Maturity-onset diabetes of the young, type 3	6	Pathogenic	1
HNF4A	20-43042354-C>T	p.Arg136Trp	7.73E-05	rs137853336	Pathogenic	Maturity-onset diabetes of the young, type 1	9.5	Pathogenic	1
HNF4A	20-43043289-C>T	p.Pro212Leu	2.00E-04	rs201749293	.	.	7.5	PD	1
HNF4A	20-43052763-G>A	p.Arg333His	4.10E-06	rs1375557127	.	.	9	PD	1
HPR	16-72107692-CGTGT>C	p.Val11fs	7.80E-03	rs756232432	.	.	10	PD	1
HPR	16-72110808-C>G	p.Thr329Ser	2.84E-05	rs557750628	.	.	7	PD	1
HSD17B4	5-118829592-G>T	p.Trp298Cys	4.07E-06	rs368744809	Likely pathogenic	Bifunctional peroxisomal enzyme deficiency	10	Pathogenic	1
HSD3B1	1-120054192-G>T	p.Arg73Ile	4.10E-03	rs4986952	.	.	7.8	PD	3
HSD3B2	1-119958047-G>T	p.Gly2Val	4.00E-04	rs116449508	.	.	9.5	PD	1
IDH1	2-209108301-T>C	p.Tyr183Cys	9.90E-03	rs34599179	not provided	not specified	8.9	PD	2
IDI2	10-1066786-AG>A	p.Leu96fs	1.00E-03	rs55836842	.	.	10	PD	1
INSR	19-7125518-C>T	p.Val1012Met	8.50E-03	rs1799816	Uncertain significance|other|Likely benign	Diabetes mellitus type 2|not specified|Insulin-resistant diabetes mellitus AND acanthosis nigricans	7.8	PD	1
INSR	19-7172362-G>C	p.Leu403Val	0.00E+00	.	.	.	8.5	PD	1
IRF2BP2	1-234745166-G>A (1 homozygote)		3.90E-03	rs76362154	.	.	10	PD	3
IRS1	2-227660169-G>A	p.Arg1096Trp	0.00E+00	rs199554914	.	.	9.5	PD	1
IRS1	2-227660781-T>C	p.Ser892Gly	2.70E-03	rs1801277	.	.	7.2	PD	3
KCNK17	6-39281918-C>T	p.Trp60*	7.58E-05	rs781115980	.	.	10	PD	1
LBR	1-225591091-C>T	p.Glu588Lys	8.12E-06	rs138067182	.	.	9	PD	1
LBR	1-225605780-G>A	p.Pro248Leu	1.00E-04	rs140008883	.	.	8	PD	1
LCAT	16-67976824-G>A	p.Arg123Cys	8.13E-06	rs140068549	.	.	10	PD	1

LCAT	16-67977851-C>T	p.Val52Met	2.76E-05	rs763018899	.	.	8.5	PD	1
LDLR	19-11215918-C>CG	p.Glu113fs	4.07E-06	rs752191968	.	.	10	PD	1
LDLR	19-11216232-ATGG>A	p.Gly219del	2.85E-05	rs758036807	Pathogenic/Likely pathogenic	Familial hypercholesterolemia	10	Pathogenic	1
LDLR	19-11216263-C>G	p.Asp227Glu	8.18E-06	rs121908028	other/Pathogenic/Pathogenic/Likely pathogenic	Familial hypercholesterolemia	10	PD	1
LDLR	19-11218100-T>C	p.Cys284Arg	0.00E+00	.	Uncertain significance,Likely pathogenic,Likely pathogenic	Familial hypercholesterolemia,Familial hypercholesterolemia,Familial hypercholesterolemia	10	Pathogenic	1
LDLR	19-11218162-C>G	p.Asp304Glu	0.00E+00	.	other	Familial hypercholesterolemia	10	PD	1
LDLR	19-11218182-A>C	p.Lys311Thr	0.00E+00	.	Likely pathogenic,Likely benign	Familial hypercholesterolemia,Familial hypercholesterolemia	8	Pathogenic	1
LDLR	19-11221435-C>T	p.Arg350*	4.07E-06	rs769737896	Pathogenic	Familial hypercholesterolemia	10	Pathogenic	1
LDLR	19-11224005-C>T	p.Thr413Met	3.66E-05	rs368562025	Uncertain significance|other	Hypercholesterolaemia|Familial hypercholesterolemia	9	PD	1
LDLR	19-11224405-A>G	p.Lys518Arg	4.06E-06	.	Likely benign	Familial hypercholesterolemia	6.5	PD	1
LDLR	19-11224416-A>C	p.Ile522Leu	4.06E-06	.	.	.	7.5	PD	1
LDLR	19-11227645-G>T	p.Ala606Ser	8.12E-05	rs72658865	Uncertain significance,Likely pathogenic|not provided|other	Familial hypercholesterolemia,Hypercholesterolaemia|not provided|Familial hypercholesterolemia	6.5	Pathogenic	1
LDLR	19-11231114-C>T	p.Gln686Ter	0.00E+00	rs879255126	Pathogenic	Familial hypercholesterolemia	10	Pathogenic	1
LDLR	19-11233979-CT>C	p.Pro757fs	8.13E-06	.	Pathogenic	Familial hypercholesterolemia	10	Pathogenic	1
LDLR	19-11240278-G>A	p.Val827Ile	1.00E-03	rs137853964	other|not provided|Uncertain significance	Familial hypercholesterolemia|not provided|Hypercholesterolaemia	8.5	PD	2
LILRA3	19-54803483-AG>A (homozygote)	p.Leu131fs	5.90E-03	rs201804218	.	.	10	PD	1
LILRA3	19-54803979-C>G (homozygote)	splice variant	2.08E-02	rs11574607	.	.	10	PD	1
LIPE	19-42905973-CCCCCCGCAGCCCCCGTCTA>C	p.Val1068fs	8.00E-04	rs587777699	Pathogenic	Familial partial lipodystrophy 6	10	Pathogenic	1
LIPE	19-42906125-G>A	p.His1024Tyr	0.00E+00	.	.	.	8	PD	1
LIPE	19-42930751-G>T	p.Ser184*	2.00E-04	rs149927060	.	.	10	PD	1
LIPG	18-47093814-G>T	p.Met94Ile	0.00E+00	.	.	.	7	PD	1
LIPG	18-47101952-C>T	p.Ala262Val	0.00E+00	rs1347001906	.	.	8.5	PD	1
LMF1	16-919894-C>T	p.Ala469Thr	8.00E-04	rs181731943	.	.	7	PD	2

LMF1	16-929680-G>A	p.His263Tyr	3.02E-05	rs746165846	.	.	7	PD	1
LMF1	16-960947-C>G	p.Arg216Thr	0.00E+00	.	.	.	7	PD	1
LMF2	22-50943099-G>A	p.Pro499Ser	5.00E-04	rs150553931	.	.	7	PD	1
LMF2	22-50944625-C>T	p.Glu205Lys	3.83E-05	rs202067766	.	.	9	PD	1
LPA	6-160952816-T>C	p.Tyr2023Cys	1.00E-02	rs41267807	.	.	10	PD	6
LPA	6-160963771-C>A	p.Gly1823Val	8.00E-03	rs76062330	.	.	10	PD	3
LPA	6-160963774-C>G	p.Gly1822Ala	4.80E-03	rs41265936	.	.	7.2	PD	2
LPA	6-161006111-C>T	p.Trp1419*	2.84E-05	rs373159484	.	.	10	PD	1
LPA	6-161022107-C>T	p.Arg990Gln	7.60E-03	rs41259144	.	.	7.8	PD	2
LPCAT3	12-7090770-C>T	p.Gly162Arg	4.06E-06	rs782788225	.	.	8	PD	1
LPIN1	2-11919668-C>A	p.Thr334Lys	5.00E-04	rs141555457	.	.	9	PD	1
LPIN1	2-11927213-G>T	p.Glu570Asp	0.00E+00	.	.	.	7	PD	1
LPIN2	18-2926788-C>A	p.Glu576*	0.00E+00	.	.	.	10	PD	1
LPIN2	18-2939542-T>G	p.Glu253Ala	4.06E-06	.	.	.	7.5	PD	1
LPIN3	20-39974453-G>A	p.Val4Met	1.00E-04	rs146667115	.	.	9	PD	2
LPIN3	20-39980511-G>A	p.Asp385Asn	8.12E-06	rs775303757	.	.	9	PD	1
LPL	8-19805708-G>A (1 homozygote)	p.Asp36Asn	1.40E-02	rs1801177	Pathogenic|other|Likely benign	Hyperlipidemia, familial combined|Coronary heart disease|Hyperlipoproteinemia, type I	3.9	PD	1
LPL	8-19811733-G>A (1 homozygote)	p.Gly215Glu	2.00E-04	rs118204057	Pathogenic	Hyperlipoproteinemia, type I	7	Pathogenic	2
LRP1	12-57573249-G>A	p.Glu1626Lys	4.06E-06	rs761763756	.	.	9.5	PD	1
LRP1	12-57577912-G>A	p.Ala1992Thr	1.63E-05	rs769741036	.	.	8.5	PD	1
LRP1	12-57578158-C>T	p.Arg2037Trp	8.94E-05	rs193167795	.	.	8	PD	1
LRP1	12-57587039-G>A	p.Gly2546Ser	3.10E-03	rs113379328	.	.	8.5	PD	1
LRP1	12-57592090-G>A	p.Arg3145His	4.49E-05	rs370217380	.	.	7	PD	1
LRP1	12-57602881-A>T	p.Tyr4054Phe	1.90E-03	rs79435985	.	.	8.9	PD	1
LRP4	11-46880592-G>C	p.Ser1887Cys	1.00E-03	rs149082597	Uncertain significance	not specified	7.5	PD	1
LRP4	11-46911911-C>T	p.Arg611His	5.28E-05	rs140282454	.	.	9	PD	1
LRP8	1-53724007-C>T	p.Met731Ile	0.00E+00	.	.	.	7.5	PD	1
LRP8	1-53724026-A>C	p.Met725Arg	2.00E-04	rs139703435	.	.	9	PD	1
LTC4S	5-179222858-G>A	splice variant	2.20E-03	rs137887093	.	.	10	PD	3

MARC1	1-220960288-T>C	p.Met1?	2.80E-03	rs537057905	.	.	10	PD	1
MC4R	18-58038753-CAA>C	p.Tyr276fs	0.00E+00	rs1490148732	.	.	10	PD	1
MC4R	18-58038756-T>C	p.Tyr276Cys	6.50E-05	rs748627503	.	.	8.5	PD	1
MC4R	18-58038777-A>T	p.Ile269Asn	1.00E-03	rs79783591	Likely pathogenic	Obesity	6	Pathogenic	1
ME1	6-84108085-C>T	splice variant	5.30E-03	rs78734745	.	.	10	PD	4
MSR1	8-16012594-G>A	p.Arg311*	8.20E-03	rs41341748	Uncertain significance|Pathogenic	Malignant tumor of prostate|Hereditary cancer-predisposing syndrome	10	Pathogenic	3
MSR1	8-16035410-C>T	p.Ala48Thr	2.00E-04	rs143927568	.	.	7.5	PD	1
MTOR	1-11193166-C>T	p.Ala1779Thr	1.22E-05	rs755987187	.	.	6.5	PD	1
MUT	6-49412463-T>G	p.Lys522Thr	8.16E-06	rs144665233	.	.	9	PD	1
MUT	6-49419386-C>T	p.Met375Ile	1.40E-03	rs148091558	Uncertain significance|Uncertain significance	not specified|Methylmalonic acidemia	8.5	PD	2
MVD	16-88721821-C>T	p.Arg228Gln	2.27E-05	rs770939767	.	.	7	PD	1
MVD	16-88722123-T>G	p.Lys207Gln	6.23E-06	.	.	.	8	PD	1
MVD	16-88723906-G>A	p.Ser114Leu	5.36E-05	rs372683488	.	.	10	PD	1
MYL5	4-673778-T>C	p.Phe88Ser	6.70E-03	rs2228354	.	.	10	PD	5
MYL5	4-674357-G>A	p.Gly118Arg	8.13E-06	rs757460768	.	.	9	PD	1
NAT2	8-18257761-GT>G	p.Gln85fs	0.00E+00	.	.	.	10	PD	1
NCOR1	17-15976880-A>G	p.Ile1225Thr	4.16E-05	rs759806084	.	.	7	PD	1
NDST1	5-149901210-C>T	p.Arg132Cys	1.00E-04	rs200193567	.	.	7.5	PD	1
NDST1	5-149925028-C>T	p.Arg709Trp	0.00E+00	.	.	.	9	PD	1
NPC1	18-21118528-G>C	p.Pro1007Ala	1.00E-04	rs80358257	Pathogenic|Pathogenic|Pathogenic|Pathogenic|Pathogenic|Pathogenic|Pathogenic|Pathogenic|Pathogenic	Niemann-Pick disease type C1|not provided|Niemann-Pick disease, type C|Ataxia|Cataplexy|Cognitive impairment|Headache|Postural instability|Speech apraxia	9	Pathogenic	2
NPC1L1	7-44553153-G>A	p.Arg1325*	3.68E-05	rs764999826	.	.	10	PD	1
NPC1L1	7-44573442-C>T	p.Arg726Gln	7.59E-05	rs765299082	.	.	8	PD	1
NPC1L1	7-44578747-G>A	p.Arg417Trp	1.30E-03	rs139659653	.	.	7	PD	1
NR0B2	1-27240089-G>A	p.Pro115Ser	1.00E-04	rs140901243	.	.	6.5	PD	1
NR0B2	1-27240272-G>A	p.Arg54Cys	3.00E-04	rs113654931	.	.	6.5	PD	1

NR1H3	11-47282886-A>AC	p.Gln156fs	8.54E-05	rs779801455	.	.	10	PD	1
NR1H4	12-100904744-C>T	p.Arg100Cys	2.00E-04	rs150295715	.	.	6.5	PD	1
NR1H4	12-100904814-G>A	p.Arg123His	2.03E-05	rs565451641	.	.	9.5	PD	1
NR1H4	12-100926308-T>C	p.Met183Thr	3.70E-03	rs61755050	Likely benign	not specified	9	PD	1
NR2F2	15-96875542-A>AAGC	p.Gln75dup	3.00E-05	rs754770347	Pathogenic	Congenital heart defects, multiple types, 4	10	Pathogenic	1
NR5A2	1-200017834-G>C	p.Arg333Pro	4.10E-03	rs61755054	.	.	8.9	PD	2
OR4C46	11-51515412-TGGTCACCATCACTGCCAGCCCATCACTGG>T	p.Thr46fs	5.80E-03	rs372979385	.	.	10	PD	1
OR4C46	11-51515660-C>A	p.Pro127Thr	8.12E-06	rs143387889	.	.	7	PD	1
OSBPL1A	18-21752415-G>A	p.Thr709Ile	7.80E-03	rs45467295	.	.	6.7	PD	6
OSBPL1A	18-21883640-TG>T	p.Asn379fs	4.47E-05	rs764967609	.	.	10	PD	1
OSBPL1A	18-21912975-G>A	p.Arg186Trp	2.00E-04	rs201206540	.	.	9	PD	1
PBX4	19-19680385-C>T	p.Arg214Gln	3.26E-05	rs750891971	.	.	8.5	PD	1
PBX4	19-19710164-GA>G	p.Leu44fs	3.00E-04	rs754292125	.	.	10	PD	1
PCCA	13-100909911-G>C	p.Asp234His	0.00E+00	.	.	.	8	PD	1
PCCA	13-100962151-A>G	p.Tyr473Cys	0.00E+00	.	.	.	8.5	PD	1
PCCB	3-136012689-C>T	p.Thr280Ile	1.22E-05	rs779581071	.	.	7	PD	1
PCCB	3-136016845-G>A	p.Arg303Gln	2.00E-03	rs150555106	Uncertain significance	not specified	9.5	PD	3
PCCB	3-136019928-A>G	p.Tyr345Cys	1.00E-04	rs148578333	.	.	10	PD	1
PCCB	3-136045708-A>G	p.Asn416Ser	1.00E-04	rs151078515	Uncertain significance	Propionic acidemia	7.5	PD	1
PCCB	3-136047691-C>T	p.Ala528Val	2.00E-03	rs142403318	Benign	Propionic acidemia	8.5	PD	1
PCSK9	1-55518374-C>T	p.Arg237Trp	7.00E-04	rs148195424	Likely pathogenic(1);Uncertain significance(4)	Familial hypercholesterolemia	9.5	PD	2
PCTP	17-53828504-TGGGAG>T	p.Trp14fs	2.00E-04	rs759493316	.	.	10	PD	1
PCTP	17-53848473-A>G	p.Tyr89Cys	8.13E-06	.	.	.	7.5	PD	1
PDIA2	16-334393-C>T	p.Pro69Leu	4.26E-05	rs372266436	.	.	8.5	PD	1
PDIA2	16-334694-C>T	p.Arg148*	8.75E-05	rs370453080	.	.	10	PD	1
PDIA2	16-334920-C>T	p.Gln195*	8.80E-03	rs45619835	.	.	10	PD	5
PDIA2	16-336700-ACT>A	p.Leu464fs	7.00E-03	rs201624048	.	.	10	PD	2
PDIA2	16-336888-C>CA	p.Asn493fs	9.10E-03	rs199887121	.	.	10	PD	2

PEPD	19-33882259-G>A	p.Pro365Leu	3.00E-04	rs200183031	Uncertain significance	Prolidase deficiency	8.5	PD	1
PEPD	19-33904511-C>T	p.Arg237His	2.00E-04	rs577079343	Uncertain significance	Prolidase deficiency	8.5	PD	1
PGS1	17-76394420-C>T	p.Arg167Trp	2.03E-05	rs369680559	.	.	7	PD	1
PHC1	12-9085218-C>T	p.Gln389*	6.10E-03	rs201644380	.	.	10	PD	3
PHYH	10-13330437-G>C	p.Arg201Gly	1.30E-03	rs143957922	Uncertain significance	Phytanic acid storage disease	8.5	PD	1
PHYH	10-13336486-G>A	p.Thr119Met	1.30E-03	rs34571629	Uncertain significance	Phytanic acid storage disease	7.5	PD	1
PKD1L3	16-72013812-C>T	splice variant	4.00E-04	rs200969421	.	.	10	PD	1
PLA2G2C	1-20501497-CACCT>C	p.Ser63fs	9.00E-04	rs370008327	.	.	10	PD	1
PLA2G2D	1-20440621-T>TC	p.Thr142fs	8.15E-06	rs755262572	.	.	10	PD	1
PLA2G2D	1-20442878-C>T	p.Gly45Ser	4.00E-03	rs139125782	.	.	8	PD	3
PLA2G3	22-31533855-G>A	p.Arg303*	1.20E-03	rs151265075	.	.	10	PD	1
PLA2G3	22-31536134-C>CT	p.Ser70fs	4.30E-03	rs573436695	.	.	10	PD	1
PLA2G4B	15-42139532-CA>C	splice variant	6.00E-04	rs545674028	.	.	10	PD	1
PLA2G4E	15-42292482-T>C	splice variant	6.04E-05	rs770379595	.	.	10	PD	1
PLA2G6	22-38508551-C>A	p.Ala746Ser	6.73E-05	rs563222818	.	.	7	PD	1
PLB1	2-28805279-G>A	p.Arg547Gln	3.00E-04	rs148210058	.	.	7	PD	1
PLIN1	15-90210405-G>GC	p.Ala324fs	1.64E-05	rs760551223	.	.	10	PD	1
PLIN2	9-19116418-A>AT	p.Met381fs	0.00E+00	.	.	.	10	PD	1
PNLIP	10-118315002-T>G	p.Ile265Arg	4.47E-05	rs377358755	.	.	9	PD	1
PNLIPRP1	10-118354278-G>A	p.Val123Met	1.00E-03	rs62641720	.	.	9.5	PD	1
PNLIPRP1	10-118359557-AG>A	p.Gly272fs	9.35E-05	rs782651844	.	.	10	PD	1
PNLIPRP2	10-118386390-G>C	p.Trp115Cys	8.72E-06	.	.	.	8.5	PD	2
PNLIPRP2	10-118389458-G>A	p.Trp194*	2.00E-04	rs185596380	.	.	10	PD	1
PNPLA2	11-821810-G>A	p.Gly124Ser	6.50E-05	rs377534825	.	.	9.5	PD	1
PNPLA2	11-824088-C>T	p.Pro337Leu	5.69E-05	rs370723127	.	.	8.5	PD	1
PNPLA3	22-44323048-G>A	splice variant	3.25E-05	rs372217781	.	.	10	PD	1
PON3	7-95024007-G>A	p.Arg32*	1.50E-03	rs147006695	.	.	10	PD	1
PPAP2A	5-54721149-G>A	p.Ser248Leu	9.38E-05	rs146179969	.	.	8	PD	1
PPAP2A	5-54763925-G>C	p.Ser89Cys	5.30E-05	rs199530852	.	.	7	PD	1
PPARA	22-46627932-G>T	p.Ala319Ser	2.44E-05	.	.	.	7.5	PD	1
PPARA	22-46627952-G>T	p.Met325Ile	3.00E-04	rs201836521	.	.	7	PD	1

PRKACA	19-14208646-T>C	p.His159Arg	0.00E+00	.	.	.	10	PD	1
PRKACG	9-71628548-GC>G	p.Ala154fs	0.00E+00	.	.	.	10	PD	1
PRKD1	14-30194877-G>A	p.Pro90Ser	3.00E-04	rs45458201	.	.	8	PD	1
PXN	12-120652762-A>G	p.Phe396Leu	1.63E-05	.	.	.	9.5	PD	1
RARA	17-38508695-G>A	p.Gly264Asp	2.00E-04	rs200827868	.	.	10	PD	1
RDH11	14-68156941-G>A	p.Arg48Trp	2.00E-04	rs139691140	.	.	9	PD	1
RORC	1-151789251-C>T	p.Ala117Thr	2.42E-05	rs201373806	.	.	7	PD	1
RXRG	1-165378892-G>A	p.Arg317Cys	1.47E-05	rs749399300	.	.	8.5	PD	1
SBNO1	12-123815909-A>C	p.Phe308Cys	8.14E-06	rs140243682	.	.	8.5	PD	1
SCAP	3-47459258-G>A	p.Arg836*	0.00E+00	.	.	.	10	PD	1
SCAP	3-47461185-C>T	p.Val525Ile	1.70E-05	rs778268038	.	.	8	PD	1
SLC10A2	13-103701770-C>T	p.Gly263Glu	0.00E+00	.	.	.	10	PD	1
SLC10A2	13-103710685-G>A	p.Pro142Leu	9.00E-04	rs117447044	.	.	7	PD	1
SLC25A1	22-19164440-G>A	p.Leu184Phe	4.47E-05	rs199588991	.	.	8.5	PD	1
SLC2A4	17-7187812-C>T	p.Arg246Cys	1.22E-05	.	.	.	8.5	PD	1
SLC7A10	19-33703503-G>A	p.Thr184Met	1.10E-03	rs148500073	.	.	9	PD	1
SLCO1B1	12-21375289-C>T	p.Arg580*	1.60E-03	rs71581941	Pathogenic	Rotor syndrome	10	Pathogenic	1
SLCO1B3	12-21008077-G>T	p.Gly67Val	0.00E+00	.	.	.	9	PD	1
SLCO1B7	12-21229413-G>A	p.Gly653Glu	7.50E-03	rs188817665	.	.	8.9	PD	2
SOX17	8-55370796-C>A	p.Ala33Asp	3.60E-03	rs189384157	.	.	9	PD	2
SPHK2	19-49129134-CT>C	p.Phe73fs	1.00E-03	rs767156752	.	.	10	PD	1
SPTLC2	14-77978652-G>A	p.Thr555Met	5.00E-03	rs138652708	Likely benign	Hereditary sensory and autonomic neuropathy type IC	6.7	PD	1
SRD5A3	4-56236247-TTTTTG>T	p.Phe318fs	5.00E-04	rs565935886	.	.	10	PD	1
STAB1	3-52544160-G>A	p.Cys846Tyr	6.50E-05	rs370482937	.	.	9	PD	1
STAB1	3-52546910-C>T	p.Arg1032Cys	5.00E-04	rs141381639	.	.	8	PD	1
STAB1	3-52553372-G>GC	p.Glu1712fs	1.00E-04	rs748877370	.	.	10	PD	1
STAB1	3-52554447-G>T	p.Arg1844Leu	1.63E-05	rs143901118	.	.	7	PD	1
STAB1	3-52554531-G>A	p.Arg1872His	1.30E-03	rs147953260	.	.	8	PD	4
STAB1	3-52556168-C>G	p.Ser2129Arg	9.00E-04	rs566837633	.	.	9	PD	1
STAR	8-38003911-G>A	p.Arg121Trp	1.70E-03	rs34908868	Uncertain significance	Congenital adrenal hyperplasia	9.5	PD	1

STARD4	5-110835762-C>T	p.Arg147Gln	6.00E-04	rs146729717	.	.	9.5	PD	1	
TCF7L2	10-114799880-A>G	p.Ile183Val	1.00E-04	rs143077353	.	.	8	PD	1	
TECR	19-14675760-G>C	splice variant	4.06E-06	.	.	.	10	PD	1	
TIAM2	6-155578137-CTGAAAATGCCACCATCGACCTAA>C	p.Glu1693fs	0.00E+00	.	.	.	10	PD	1	
TM7SF2	11-64880061-C>T	p.Arg43Cys	7.00E-04	rs371186990	.	.	7.8	PD	1	
TM7SF2	11-64882506-C>T	p.Pro282Leu	1.10E-03	rs187240326	.	.	10	PD	1	
TM7SF2	11-64883437-A>G (homozygote)	p.Asp390Gly	3.00E-04	rs200277720	.	.	10	PD	1	
TNFRSF21	6-47251796-G>C	p.Ser374Trp	8.12E-06	.	.	.	7	PD	1	
TPMT	6-18143955-C>G	p.Ala80Pro	1.70E-03	rs1800462	drug response	Thiopurine methyltransferase deficiency	10	PD	2	
TRPS1	8-116599311-C>T	p.Gly873Arg	0.00E+00	rs1296670556	.	.	7	PD	1	
TRPS1	8-116632173-G>A	p.Ser51Phe	2.03E-05	rs375022768	.	.	7	PD	1	
TTC39B	9-15172038-C>T	p.Trp676*	0.00E+00	.	.	.	10	PD	1	
TXNRD1	12-104725415-T>C	p.Ile549Thr	8.29E-06	rs778938164	.	.	9.5	PD	1	
TYW1B	7-72159743-G>A	p.Arg481*	2.03E-05	rs782150621	.	.	10	PD	1	
UCP1	4-141489053-C>T	p.Glu69Lys	3.00E-04	rs375694859	.	.	10	PD	1	
UCP1	4-141489766-G>A	p.Arg40Trp	3.60E-03	rs150067245	.	.	10	PD	1	
UGT1A10	2-234680951-C>T	p.Arg447Cys	4.50E-05	rs201427749	.	.	9	PD	1	
UGT1A10	2-234681014-G>A	p.Ala468Thr	6.10E-05	rs775532505	.	.	8.5	PD	1	
USP3	15-63852166-A>AT	p.Val216fs	0.00E+00	.	.	.	10	PD	1	
VEGFA	6-43748593-T>C	splice variant	1.00E-04	rs149528656	.	.	10	PD	2	
WWOX	16-78133763-G>C	p.Gly30Arg	5.61E-06	rs1268902626	.	.	10	PD	1	
WWOX	16-78149000-C>T	p.Arg120Trp	7.50E-03	rs141361080	other|Benign|Benign	not specified|Epileptic encephalopathy, early infantile, 1|Spinocerebellar ataxia, autosomal recessive 12	7.2	PD	2	
WWOX	16-78466512-C>G	p.Leu307Val	5.28E-05	rs200320711	.	.	9.5	PD	1	
ZNF259	11-116657232-G>A	p.Arg164*	1.22E-05	rs773265443	.	.	10	PD	1	

*Damaging prediction: number of damaging predictions out of 10 separate variant impact prediction tools. PD: probably damaging.

Supplemental Table S34. Rare heterozygous damaging variants among HDL candidate genes

Gene	Chr:Pos:Ref:Alt	AA change	gnomAD	ID	ClinVar Significance	ClinVar Disease	DP *	Prediction	Number of Patients
ABCA1	9-107556776-T>G	p.Asn1800His	3.00E-04	rs146292819	Pathogenic	ABCA1-Related Disorders	9	Pathogenic	1
ABCA1	9-107560784-C>T	p.Arg1680Gln	3.00E-04	rs150125857	Likely benign|Likely benign	Tangier disease|Familial High Density Lipoprotein Deficiency	9.5	PD	1
ABCA1	9-107566964-G>A	p.Thr1501Ile	0.00E+00	.	.	.	9.5	PD	1
ABCA1	9-107568536dupC	p.Leu1484fs	0.00E+00	.	.	.	10	PD	1
ABCA1	9-107571799-G>A	p.Leu1408Phe	4.00E-04	rs201879964	Likely benign|Likely benign	Tangier disease|Familial High Density Lipoprotein Deficiency	6.5	PD	1
ABCA1	9-107574868-C>T	p.Gly1346Glu	1.00E-04	rs762770081	Likely pathogenic	Inborn genetic diseases	9.5	PD	1
ABCA1	9-107574881-G>A	p.Arg1342Trp	1.62E-05	rs760786920	Uncertain significance|Uncertain significance	Tangier disease|Familial High Density Lipoprotein Deficiency	9.5	PD	1
ABCA1	9-107578437-G>A	p.Thr1242Met	2.03E-05	rs144923927	.	.	10	PD	1
ABCA1	9-107578515-C>A	p.Gly1216Val	4.47E-05	rs562403512	.	.	9.5	PD	1
ABCA1	9-107583758-G>A	p.Thr953Ile	0.00E+00	.	.	.	9.5	PD	1
ABCA1	9-107584879insGGTA	p.Arg909fs	0.00E+00	.	.	.	10	PD	1
ABCA1	9-107587972-A>G	p.Val845Ala	3.25E-05	rs541344598	.	.	8	PD	1
ABCA1	9-107593272-G>A	p.Thr609Met	3.25E-05	rs755276277	.	.	9	PD	1
ABCA1	9-107593329-C>T	p.Trp590*	0.00E+00	.	.	.	10	PD	1
ABCA1	9-107594878-G>A	p.Arg496Trp	6.00E-04	rs147675550	Likely benign|Likely benign	Familial High Density Lipoprotein Deficiency|Tangier disease	7	PD	1
ABCA1	9-107599263-G>A	p.Arg437Trp	4.87E-05	rs150448790	.	.	9.5	PD	1
ABCA1	9-107599797-C>T	p.Arg369His	2.44E-05	rs370223805	.	.	9.5	PD	1
ABCA1	9-107602623-T>C	p.Lys331Glu	0.00E+00	.	.	.	7.5	PD	1
ABCA1	9-107646756-G>A	p.Pro85Leu	1.40E-03	rs145183203	Likely benign|Likely benign	Tangier disease|Familial High Density Lipoprotein Deficiency	8.5	PD	1
ABCA1	9-107665929-A>G	p.Leu11Pro	4.84E-06	rs777372679	.	.	10	PD	1
ABCA8	17-66872640-G>A	p.Pro1444Leu	5.69E-05	rs372083205	.	.	7	PD	1
ABCA8	17-66878099-C>T	p.Cys1284Tyr	8.70E-03	rs34987539	.	.	9.4	PD	3
ABCA8	17-66902302-G>T	p.Leu761Ile	1.80E-03	rs140010342	.	.	9	PD	1
ABCA8	17-66915549-C>T	p.Gly561Arg	5.00E-04	rs552291569	.	.	7.5	PD	1
ACAA2	18-47317912-G>A	p.His271Tyr	8.00E-04	rs148304029	.	.	7	PD	1
ACAA2	18-47323888-G>T	p.Thr87Lys	3.25E-05	rs531271159	.	.	10	PD	1

ACAA2	18-47329071-C>G	p.Gly57Arg	9.35E-05	rs141307346	.	.	10	PD	1
ACAD11	3-132280031-G>A	p.Arg678Cys	1.63E-05	rs143271405	.	.	10	PD	1
ACAD11	3-132294680-G>C	p.Ala646Gly	6.00E-04	rs36121581	.	.	8	PD	1
ACAD11	3-132322103-C>A	p.Val531Leu	2.00E-04	rs767280144	.	.	7.5	PD	1
AMPD3	11-10508903-G>T	p.Val320Leu	6.80E-03	rs117706710	Benign	not specified	7.8	PD	1
AMPD3	11-10515022-C>T	p.Arg365Trp	3.00E-04	rs149809940	.	.	8.5	PD	1
AMPD3	11-10515088-G>A	p.Ala387Thr	4.11E-05	rs767255627	.	.	9.5	PD	1
AMPD3	11-10517141-A>G	p.Ser440Gly	1.22E-05	rs763919044	.	.	7.5	PD	1
AMPD3	11-10517252-C>T	p.Arg477Cys	6.09E-05	rs147091692	.	.	9.5	PD	1
APOA1	11-116706768-A>C	p.Leu187Arg	0.00E+00	.	.	.	9.5	PD	1
APOA1	11-116707739-A>C	p.Ser60Ala	3.00E-04	rs199759119	.	.	7	PD	1
APOA1	11-116707838dupG	p.Gln29fs	1.63E-05	rs753348565	likely pathogenic	.	10	PD	1
APOC3	11-116701354-G>A	((splice variant)	1.40E-03	rs138326449	other|Pathogenic	Coronary heart disease|Hyperalphalipoproteinemia 2	10	Pathogenic	1
APOE	19-45412358-C>G	p.Arg269Gly	4.00E-04	rs267606661	Pathogenic	Familial type 3 hyperlipoproteinemia	5.5	Uncertain	1
ATG7	3-11389502-C>T	p.Pro426Leu	1.20E-03	rs143545741	.	.	9	PD	2
CD36	7-80285989-T>G	p.Val85Gly	0.00E+00	.	.	.	8.5	PD	1
CD36	7-80286003-C>T	p.Pro90Ser	1.10E-03	rs75326924	Pathogenic	Platelet glycoprotein IV deficiency	10	Pathogenic	1
CD36	7-80300449-T>G	p.Tyr325*	6.00E-03	rs3211938	Benign	Platelet glycoprotein IV deficiency	10	PD	3
CD36	7-80302113-delAACGGCTGCAG	p.Lys385fs	8.17E-06	.	.	.	10	PD	1
CD36	7-80302673-delTATT	p.Val401fs	1.00E-04	rs769354931	.	.	10	PD	1
CD93	20-23065998-C>T	p.Gly278Arg	2.00E-04	rs150125306	.	.	8	PD	1
CD93	20-23066727-C>A	p.Ala35Ser	1.33E-05	rs765091675	.	.	7	PD	1
CELSR2	1-109808789-G>A	p.Gly1992Arg	7.00E-04	rs12567377	.	.	8.5	PD	1
COBLL1	2-165578958-G>T	p.Arg312Ser	5.45E-06	rs553945564	.	.	7.5	PD	1
CPS1	2-211421462-C>T	p.Thr8Met	1.00E-04	rs150314086	Uncertain significance	Congenital hyperammonemia, type I	7	PD	1
CPS1	2-211442212-G>A	p.Gly156Glu	6.40E-03	rs114819130	Uncertain significance	Congenital hyperammonemia, type I	8.3	PD	4
CPS1	2-211454831-G>A	p.Arg244Gln	3.00E-04	rs147294932	.	.	8	PD	1
CPS1	2-211466932-G>A	p.Asp578Asn	1.10E-03	rs142916171	.	.	8.5	PD	1
CPS1	2-211473128-C>A	p.Pro752Thr	3.26E-05	rs139740322	.	.	8	PD	1
CYP27A1	2-219679419-G>C	p.Gly472Ala	9.35E-05	rs200883871	Pathogenic	Cholestanol storage disease	10	Pathogenic	1
DAGLB	7-6449535delC	p.Val651fs	0.00E+00	.	.	.	10	PD	1
FAM13A	4-89660218-T>A	p.Lys842Ile	0.00E+00	.	.	.	9.5	PD	1
FTO	16-53859930-C>G	p.Pro93Arg	1.00E-04	rs151263395	.	.	7.5	PD	2

GOT2	16-58742107-G>C	p.Leu421Val	0.00E+00	.	.	.	7	PD	1
GOT2	16-58743431-T>G	p.Met354Leu	0.00E+00	.	.	.	7.5	PD	1
GOT2	16-58743442-C>T	p.Arg350His	2.44E-05	rs148828634	.	.	9	PD	1
GOT2	16-58752439-C>T	p.Asp197Asn	8.00E-04	rs149988435	.	.	9	PD	1
GOT2	16-58753152-G>A	p.Thr128Ile	1.62E-05	rs746079905	.	.	7.5	PD	1
HAS1	19-52216738-C>T	p.Trp567*	2.00E-04	rs200444967	.	.	10	PD	1
HNF4A	20-43042354-C>T	p.Arg136Trp	7.73E-05	rs137853336	Pathogenic	Maturity-onset diabetes of the young, type 1	9.5	Pathogenic	1
HNF4A	20-43043289-C>T	p.Pro212Leu	2.00E-04	rs201749293	.	.	7.5	PD	1
HNF4A	20-43052763-G>A	p.Arg333His	4.10E-06	rs1375557127	.	.	9	PD	1
IRS1	2-227660169-G>A	p.Arg1096Trp	0.00E+00	rs199554914	.	.	9.5	PD	1
IRS1	2-227660781-T>C	p.Ser892Gly	2.70E-03	rs1801277	.	.	7.2	PD	3
LCAT	16-67976824-G>A	p.Arg123Cys	8.13E-06	rs140068549	.	.	10	PD	1
LCAT	16-67977851-C>T	p.Val52Met	2.76E-05	rs763018899	.	.	8.5	PD	1
LILRA3	19-54803488delG	p.Leu131fs	5.90E-03	rs201804218	.	.	10	PD	1
LILRA3	19-54803979-C>G	(splice variant)	2.08E-02	rs11574607	.	.	10	PD	1
LIPE	19-42905992 42906010dup	p.Val1068fs	8.00E-04	rs587777699	Pathogenic	Familial partial lipodystrophy 6	10	Pathogenic	1
LIPE	19-42906125-G>A	p.His1024Tyr	0.00E+00	.	.	.	8	PD	1
LIPE	19-42930751-G>T	p.Ser184*	2.00E-04	rs149927060	.	.	10	PD	1
LIPG	18-47093814-G>T	p.Met94Ile	0.00E+00	.	.	.	7	PD	1
LIPG	18-47101952-C>T	p.Ala262Val	0.00E+00	rs1347001906	.	.	8.5	PD	1
LPL	8-19805708-G>A	p.Asp36Asn (homozygous)	1.40E-02	rs1801177	Pathogenic|other|Likely benign	Hyperlipidemia, familial combined|Coronary heart disease|Hyperlipoproteinemia, type I	3.9	PD	1
LPL	8-19811733-G>A	p.Gly215Glu	2.00E-04	rs118204057	Pathogenic	Hyperlipoproteinemia, type I	7	Pathogenic	3
LRP1	12-57573249-G>A	p.Glu1626Lys	4.06E-06	rs761763756	.	.	9.5	PD	1
LRP1	12-57577912-G>A	p.Ala1992Thr	1.63E-05	rs769741036	.	.	8.5	PD	1
LRP1	12-57578158-C>T	p.Arg2037Trp	8.94E-05	rs193167795	.	.	8	PD	1
LRP1	12-57587039-G>A	p.Gly2546Ser	3.10E-03	rs113379328	.	.	8.5	PD	1
LRP1	12-57592090-G>A	p.Arg3145His	4.49E-05	rs370217380	.	.	7	PD	1
LRP1	12-57602881-A>T	p.Tyr4054Phe	1.90E-03	rs79435985	.	.	8.9	PD	1
LRP4	11-46880592-G>C	p.Ser1887Cys	1.00E-03	rs149082597	Uncertain significance	not specified	7.5	PD	1
LRP4	11-46911911-C>T	p.Arg611His	5.28E-05	rs140282454	.	.	9	PD	1
MC4R	18-58038753delAA	p.Tyr276fs	0.00E+00	rs1490148732	.	.	10	PD	1
MC4R	18-58038756-T>C	p.Tyr276Cys	6.50E-05	rs748627503	Uncertain significance	Obesity	8.5	PD	1

MC4R	18-58038777-A>T	p.Ile269Asn	1.00E-03	rs79783591	Likely pathogenic	Obesity	6	Pathogenic	1
NPC1	18-21118528-G>C	p.Pro1007Ala	1.00E-04	rs80358257	Pathogenic|Pathogenic|Pathogenic|Pathogenic|Pathogenic|Pathogenic|Pathogenic|Pathogenic	Niemann Pick disease type C1| not provided|Niemann-Pick disease, type C|Ataxia|Cataplexy|Cognitive impairment|Headache|Postural instability|Speech apraxia	9	Pathogenic	2
NR0B2	1-27240089-G>A	p.Pro115Ser	1.00E-04	rs140901243	.	.	6.5	PD	1
NR0B2	1-27240272-G>A	p.Arg54Cys	3.00E-04	rs113654931	Uncertain significance	.	6.5	PD	1
NR1H3	11-47282893dupC	p.Gln156fs	8.54E-05	rs779801455	.	.	10	PD	1
OR4C46	11-51515417 51515445del	p.Thr46fs	5.80E-03	rs372979385	.	.	10	PD	1
OR4C46	11-51515660-C>A	p.Pro127Thr	8.12E-06	rs143387889	.	.	7	PD	1
OSBPL1A	18-21752415-G>A	p.Thr709Ile	7.80E-03	rs45467295	.	.	6.7	PD	6
OSBPL1A	18-21883642delG	p.Asn379fs	4.47E-05	rs764967609	.	.	10	PD	1
OSBPL1A	18-21912975-G>A	p.Arg186Trp	2.00E-04	rs201206540	.	.	9	PD	1
PEPD	19-33882259-G>A	p.Pro365Leu	3.00E-04	rs200183031	Uncertain significance	Prolidase deficiency	8.5	PD	1
PEPD	19-33904511-C>T	p.Arg237His	2.00E-04	rs577079343	Uncertain significance	Prolidase deficiency	8.5	PD	1
PGS1	17-76394420-C>T	p.Arg167Trp	2.03E-05	rs369680559	.	.	7	PD	1
PON3	7-95024007-G>A	p.Arg32*	1.50E-03	rs147006695	Uncertain significance	.	10	PD	1
SBNO1	12-123815909-A>C	p.Phe308Cys	8.14E-06	rs140243682	.	.	8.5	PD	1
STAB1	3-52544160-G>A	p.Cys846Tyr	6.50E-05	rs370482937	.	.	9	PD	1
STAB1	3-52546910-C>T	p.Arg1032Cys	5.00E-04	rs141381639	.	.	8	PD	1
STAB1	3-52553378dupC	p.Glu1712fs	1.00E-04	rs748877370	.	.	10	PD	1
STAB1	3-52554447-G>T	p.Arg1844Leu	1.63E-05	rs143901118	.	.	7	PD	1
STAB1	3-52554531-G>A	p.Arg1872His	1.30E-03	rs147953260	.	.	8	PD	4
STAB1	3-52556168-C>G	p.Ser2129Arg	9.00E-04	rs566837633	.	.	9	PD	1
TRPS1	8-116599311-C>T	p.Gly873Arg	0.00E+00	rs1296670556	.	.	7	PD	1
TRPS1	8-116632173-G>A	p.Ser51Phe	2.03E-05	rs375022768	.	.	7	PD	1
TTC39B	9-15172038-C>T	p.Trp676*	0.00E+00	.	.	.	10	PD	1
VEGFA	6-43748593-T>C	(splice variant)	1.00E-04	rs149528656	.	.	10	PD	2
WWOX	16-78133763-G>C	p.Gly30Arg	5.61E-06	rs1268902626	.	.	10	PD	1
WWOX	16-78149000-C>T	p.Arg120Trp	7.50E-03	rs141361080	other|Benign|Benign	not specified|Epileptic encephalopathy, early infantile, 1|Spinocerebellar ataxia, autosomal recessive 12	7.2	PD	2
WWOX	16-78466512-C>G	p.Leu307Val	5.28E-05	rs200320711	.	.	9.5	PD	1
ZNF259	11-116657232-G>A	p.Arg164*	1.22E-05	rs773265443	.	.	10	PD	1

*Damaging prediction: number of damaging predictions out of 10 separate variant impact prediction tools. PD: probably damaging

Supplemental Table S5. CNV Analysis: Rare Copy Gains Identified Using XHMM.

Type	Chr	Start	End	# Genes	All genes	1000G MAF	Decipher MAF
DUP	chr20	29845466	30115385	9	*DEFB115,DEFB116,DEFB118,DEFB119,DEFB121,DEFB123,DEFB124,HM13,REM1*	7.99E-04	6.76E-04
DUP	chr16	28128638	28192352	1	*XPO6*	NA	NA
DUP	chr19	54327139	54401882	3	*MYADM,NLRP12,PRKCG*	NA	8.45E-04
DUP	chr22	36863850	36962540	4	*CACNG2,EIF3D,FOXRED2,TXN2*	NA	NA
DUP	chr12	52238456	52285124	1	*ANKRD33*	NA	NA
DUP	chr17	57643033	57728677	2	*CLTC,DHX40*	NA	1.69E-04
DUP	chr19	47761815	47922338	6	*C5AR1,C5AR2,CCDC9,DHX34,INAFM1,MEIS3*	NA	1.69E-04
DUP	chr11	47587174	47869972	9	*AGBL2,C1QTNF4,FAM180B,FNBP4,KBTBD4,MTCH2,NDUFS3,NUP160,PTPMT1*	NA	NA
DUP	chr19	56200235	56223956	2	*EPN1,NLRP9*	NA	NA
DUP	chr1	245180543	245319985	2	*EFCAB2,KIF26B*	NA	NA
DUP	chr2	231175868	231264957	2	*SP140,SP140L*	NA	3.38E-04
DUP	chr16	560659	583998	1	*RAB11FIP3*	NA	NA
DUP	chr17	39670274	39684499	2	*KRT15,KRT19*	7.99E-04	NA
DUP	chr19	7976134	7982744	2	*MAP2K7,TGFBR3L*	NA	NA
DUP	chr1	107866903	108328246	2	*NTNG1,VAV3*	NA	NA
DUP	chr14	45430089	45542764	1	*FAM179B,KLHL28*	NA	NA
DUP	chr3	126135158	126261421	4	*CFAP100,CHST13,UROC1,ZXDC*	NA	NA
DUP	chr3	128532169	128880154	9	*ACAD9,EFCC1,GP9,ISY1,ISY1-RAB43,KIAA1257,RAB43,RAB7A*	2.00E-04	1.69E-04
DUP	chr2	27304950	27317802	2	*EMILIN1,KHK*	NA	NA
DUP	chr19	4219587	4229614	2	*ANKRD24,EBI3*	NA	NA
DUP	chr2	105977739	106055113	1	*FHL2*	NA	NA
DUP	chr7	21582863	21678683	1	*DNAH11*	NA	NA
DUP	chr7	88423494	89887478	5	*C7orf62,CFAP69,STEAP1,STEAP2,ZNF804B*	5.99E-04	6.76E-04
DUP	chr10	99991268	100503813	5	*HPS1,HPSE2,LOXL4,PYROXD2,R3HCC1L*	NA	NA
DUP	chr11	60215121	60296933	4	*MS4A1,MS4A12,MS4A13,MS4A5*	NA	NA
DUP	chr18	60969533	61654512	14	*BCL2,HMSD,KDSR,SERPINB10,SERPINB11,SERPINB12,SERPINB13,SERPINB2,SERPINB3,SERPINB4,SERPINB5,SERPINB7,SERPINB8,VPS4B*	7.99E-04	NA
DUP	chr7	195553	330787	1	*FAM20C,WI2-2373I1.2*	7.99E-04	NA
DUP	chr6	129899657	129932755	1	*ARHGAP18*	NA	NA
DUP	chr6	393152	407598	1	*IRF4*	NA	NA
DUP	chr7	29234557	29535652	1	*CHN2*	NA	1.69E-04
DUP	chr14	102661274	102843277	5	*CINP,MOK,TECPR2,WDR20,ZNF839*	2.00E-04	NA
DUP	chr16	83520081	84014469	5	*CDH13,HSBP1,MLYCD,NECAB2,OSGIN1*	NA	NA
DUP	chr11	62600423	62607042	1	*WDR74*	NA	NA
DUP	chr3	184008344	184039891	3	*ECE2,EIF4G1,PSMD2*	3.99E-04	NA
DUP	chr3	151107766	151179272	2	*IGSF10,MED12L*	NA	NA
DUP	chr12	100422354	101018668	7	*ACTR6,DEPDC4,NR1H4,SCYL2,SLC17A8,UHRF1BP1L*	2.00E-04	NA
DUP	chr12	122825299	122848654	1	*CLIP1*	NA	NA
DUP	chr6	34730371	34855908	3	*SNRPC,TAF11,UHRF1BP1*	NA	3.38E-04
DUP	chr16	89804128	89866046	2	*FANCA,ZNF276*	NA	NA
DUP	chr11	95568453	95657118	1	*MTMR2*	NA	NA
DUP	chr22	23915452	23974210	2	*DRICH1,IGLL1*	9.98E-04	5.07E-04
DUP	chr11	48131609	48188914	1	*PTPRJ*	7.99E-04	1.69E-04
DUP	chr6	24454242	24523153	2	*ALDH5A1,GPLD1*	NA	NA
DUP	chr10	51069633	51087876	1	*PARG*	NA	0
DUP	chr11	32605433	32623945	1	*EIF3M*	NA	NA
DUP	chr16	818378	863861	5	*CHTF18,GNG13,MSLN,PRR25,RPUSD1*	7.99E-04	3.38E-04
DUP	chr8	144416909	144464821	2	*RHPN1,TOP1MT*	2.00E-04	NA

XHMM: eXome-Hidden Markov Model software.

- DEFB115,DEFB116,DEFB118,DEFB119,DEFB121,DEFB123,DEFB124: Defensins form a family of antimicrobial and cytotoxic peptides made by neutrophils
- HM13 (Histocompatibility Minor 13): Catalyzes intramembrane proteolysis of some signal peptides after they have been cleaved from a preprotein.
- REM1 (RRAD And GEM Like GTPase 1) is a GTPase and member of the RAS-like GTP-binding protein family and promotes reorganization of the actin cytoskeleton and morphological changes in the cells.
- XPO6 (Exportin 6) is a member of the importin-beta family which is regulated by the GTPase Ran to mediate transport of cargo across the nuclear

Recurrent Event: 2 patients

Supplemental Table S6. CNV Analysis: Rare Copy Losses Identified Using XHMM.

Type	Chr	Start	End	# Genes	All genes	1000G MAF	Decipher MAF
DEL	chr8	2000275	2017616	1	*MYOM2*	2.00E-04	6.76E-04
DEL	chr15	85461754	85488431	1	*SLC28A1*	2.00E-04	8.45E-04
DEL	chr19	15806745	15807895	1	*CYP4F12*	NA	0
DEL	chr4	100522763	100534297	1	***MTTP**	NA	NA
DEL	chr17	40762440	40811964	2	*TUBG1,TUBG2*	NA	1.69E-04
DEL	chr2	179974625	180041265	1	*SESTD1*	NA	NA
DEL	chr12	21007961	21392123	3	*SLCO1B1,SLCO1B3,SLCO1B7*	7.99E-04	3.38E-04
DEL	chr16	71967322	71988200	1	*PKD1L3*	2.00E-04	0
DEL	chr8	43152162	43173765	1	*POTEA*	NA	0
DEL	chr19	43857866	43859935	1	*CD177*	NA	0
DEL	chr2	108476177	108479488	1	*RGPD4*	NA	1.69E-04
DEL	chr7	121738503	121773780	0		7.99E-04	3.38E-04
DEL	chr1	216465516	216500996	1	*USH2A*	NA	NA
DEL	chr9	27217685	27294400	2	*EQTN,TEK*	3.99E-04	0
DEL	chr19	55652250	55658536	1	*TNNT1*	NA	NA
DEL	chr16	16330757	16367932	1	*NOMO3*	NA	6.76E-04
DEL	chr7	40314111	40535985	1	*SUGCT*	7.99E-04	1.69E-04
DEL	chr17	73654377	73667944	2	*RECQL5,SAP30BP*	NA	0

- **SLC28A1** (Solute Carrier Family 28 Member 1) exhibits the transport characteristics of the nucleoside transport system cit or N2 subtype (N2/cit).
- **MYOM2** (Myomesin 2) interconnects the major structure of sarcomeres, the M bands and Z discs.
- *Homozygous deletion in **MTTP** (microsomal triglyceride transfer protein) gene has been confirmed in this patient (HDL-C=22mg/dl, LDL-C=6mg/dl) as causal forAbetalipoproteinemia.
- No CNVs with HDL candidate genes were found. XHMM: eXome-Hidden Markov Model software. Recurrent Event: 2 patients
